# A systematic review of the effectiveness of counselling strategies for modern contraceptive methods: what works and what doesn’t?

**DOI:** 10.1136/bmjsrh-2019-200377

**Published:** 2019-12-11

**Authors:** Francesca L Cavallaro, Lenka Benova, Onikepe O Owolabi, Moazzam Ali

**Affiliations:** 1 University College London, London, London, UK; 2 Institute of Tropical Medicine, Antwerp, Belgium; 3 London School of Hygiene and Tropical Medicine, London, UK; 4 Guttmacher Institute, New York, New York, USA; 5 World Health Organization, Geneva, Switzerland

**Keywords:** modern contraceptives, counselling, contraceptive behaviour, method continuation, client satisfaction, family planning service provision

## Abstract

**Aim:**

The aim of this systematic review was to synthesise the evidence on the comparative effectiveness of different counselling strategies for modern contraception on contraceptive behaviour and satisfaction, and to examine their advantages and disadvantages.

**Methods:**

Six electronic databases (Medline, Embase, Global Health, Popline, CINAHL Plus, and Cochrane Library) were searched to identify publications comparing two or more contraceptive counselling strategies and reporting quantitative results on contraceptive use, uptake, continuation or switching, or client satisfaction. Studies of women or couples from any country, published in English since 1990 were considered.

**Results:**

A total of 63 publications corresponding to 61 studies met the inclusion criteria. There was substantial heterogeneity in study settings, interventions and outcome measures. Interventions targeting women initiating a method (including structured counselling on side effects) tended to show positive effects on contraceptive continuation. In contrast, the majority of studies of provider training and decision-making tools for method choice did not find evidence of an effect. Additional antenatal or postpartum counselling sessions were associated with increased postpartum contraceptive use, regardless of their timing in pregnancy or postpartum. Dedicated pre-abortion contraceptive counselling was associated with increased use only when accompanied by broader contraceptive method provision. Male partner or couples counselling was effective at increasing contraceptive use in two of five studies targeting non-users, women initiating implants or seeking abortion. High-quality evidence is lacking for the majority of intervention types.

**Conclusions:**

The evidence base and quality of studies are limited, and further research is needed to determine the effectiveness of many counselling interventions in different settings.

**Key messages**

- Detailed counselling on side effects for users initiating new methods may be effective at improving continuation (evidence of effect in three of four studies).
- Additional counselling sessions in pregnancy or postpartum may increase postpartum contraceptive uptake (evidence of effect in four of five studies).
- Caution is required in interpreting the evidence, due to a lack of high-quality evidence for most interventions, and substantial heterogeneity in study settings, interventions, and outcome measures.
- There is a need to improve reporting of studies, and to develop and evaluate novel interventions in different settings.

## Background

Ensuring access to contraception is fundamental to human rights and contributes to improved health outcomes, as recognised in Sustainable Development Goal 3.7 (universal access to sexual and reproductive healthcare services).^1^ Despite increases in contraceptive use in the last several decades, an estimated 214 million women of reproductive age have an unmet need for contraception in developing regions.^2^ Women in need of contraception or their male partners may not use a method for multiple reasons, including poor geographical or financial access, health concerns or side effects, and low decision-making power.^3–6^ Meeting the unmet need for contraception in developing regions would avert an estimated 67 million unintended pregnancies, 36 million induced abortions, and 76 000 maternal deaths each year.^2^ In addition, effective contraceptive coverage among users can be improved, as illustrated by suboptimal use^7 8^ and high discontinuation rates.^3^

Contraceptive counselling can help clients choose a method meeting their needs and preferences, manage side effects, and support method continuation or switching. High-quality counselling therefore has a high potential to strengthen efforts to reduce unmet need for contraception. The Bruce framework identified six dimensions for quality family planning (FP) services, including choice of methods, information given to clients, and interpersonal relations.^9^ Recent efforts have outlined key components for quality contraceptive counselling, including needs assessment, tailored communication, and shared decision-making.^10 11^ Attention has been called to the specific needs of adolescents, including for dual protection against pregnancy and sexually transmitted infections (STIs) and respect for adolescents’ autonomy.^12^

Despite these multiple frameworks, no clear consensus exists on how best to deliver contraceptive counselling to meet client needs and satisfaction. The WHO 2016 *Selected Practice Recommendations* include guidelines for counselling content for each method – primarily side effects and protection against STIs^13^ – while the WHO 2018 *Global Handbook for Family Planning Providers* further includes recommendations on interpersonal qualities (including respect and confidentiality).^14^ Guidance on the best mode for counselling delivery, such as face-to-face versus digital support, is limited.

Several reviews have examined counselling strategies to improve contraceptive use, finding mixed or limited effects on contraceptive behaviour and pregnancy outcomes.^15–18^ However, they focused on specific subgroups (such as adolescents) and included comparison groups with no counselling. The objective of this systematic review is to synthesise the evidence on the comparative effectiveness of contraceptive counselling strategies on contraceptive behaviour and satisfaction, and examine their advantages and disadvantages.

## Methods

The protocol for this systematic review is included in online supplementary appendix 1. We defined contraceptive counselling as the provision of contraceptive information and support for decision-making regarding contraceptive method selection (new or switching clients) or for continued use of contraceptive method (continuing clients).

10.1136/bmjsrh-2019-200377.supp1 Supplementary data

### Inclusion and exclusion criteria

We included studies comparing two or more counselling interventions and reporting quantitative findings on contraceptive behaviour (uptake, use, or continuation of a modern method, or switching modern methods at the time of counselling), or on client satisfaction with method or services. We used the WHO definition of modern methods;^19^ interventions or outcomes focused solely on barrier methods were excluded. Long-acting reversible contraception (LARC) includes contraceptive implants and intrauterine devices (IUDs).

Only studies where the comparison group received contraceptive counselling were included. We included studies of women or couples, including postabortion, postpartum and breastfeeding women, but excluded studies of women with medical conditions affecting contraceptive eligibility (such as breast cancer).^14^ Randomised controlled trials (RCTs) and non-randomised studies, as well as peer-reviewed and grey literature publications, were considered.

We considered evidence from all countries, published in English since the publication of the Bruce framework^9^ in 1990.

### Search strategy

We manually searched the key journal *Contraception* between January 2010 and October 2018 to identify keywords. Six databases (Medline, Embase, Global Health, Popline, CINAHL Plus, and Cochrane Library of Systematic Reviews) were searched for eligible studies published from 1 January 1990 to 31 October 2018, using keywords related to contraception, counselling and outcomes of interest (full search strategy in online supplementary appendix 1). Manual searches of reference lists of included studies and relevant systematic reviews identified in the search were additionally conducted.

### Article selection

**Figure 1:**
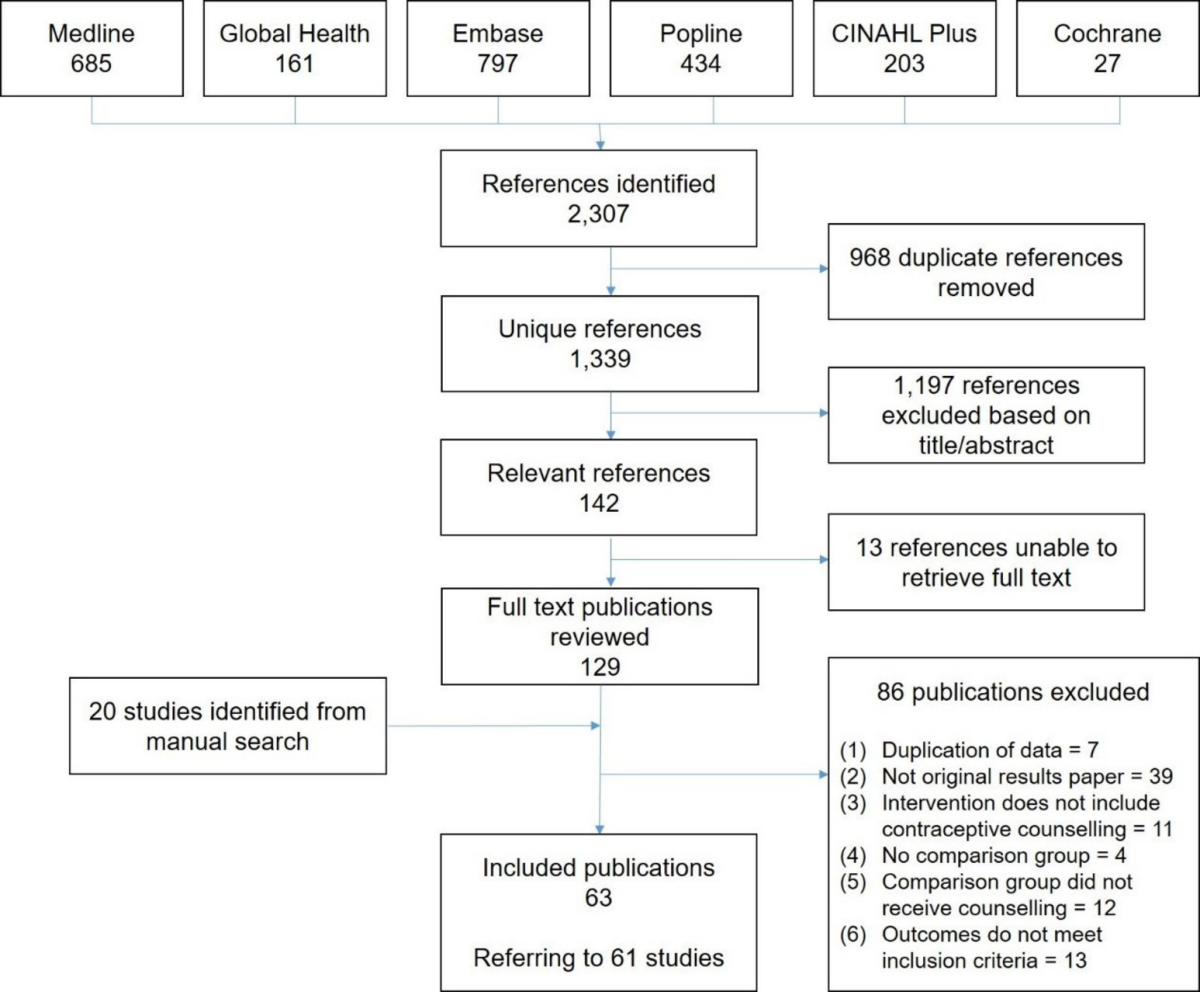
PRISMA flow diagram.

All unique identified publications were screened by one author (FC) based on title and abstract. A random 20% of excluded records were double-screened by a second reviewer (LB or OO) to ensure no relevant studies were missed; any differences in decisions between reviewers were resolved through discussion with a third reviewer (MA). All retrieved full texts were screened by one author (FC), and reason for exclusion was documented based on a hierarchical list (figure 1). Data were extracted from included studies by one author (FC) in an Excel data extraction sheet. Four included full-text articles (7%) were extracted in duplicate by a second reviewer (LB or OO), and any differences reconciled by consultation with MA.

### Data synthesis

We reported results according to intervention type and target population, with specific attention paid to adolescents and young women. The substantial variability in contraceptive counselling interventions and outcome measures prevented us from conducting a meta-analysis. We summarised findings on the effectiveness of contraceptive counselling narratively and in summary tables, and described the advantages and disadvantages of reviewed interventions based on information reported by study authors and subjective assessment of review authors.

In this review we followed PRISMA reporting guidelines, with the exception of risk of bias which was not assessed systematically.

## Results

### Systematic search results

In total, 2307 publication records were identified in the search and 968 duplicate records removed, leaving 1339 unique publications (figure 1). Based on title and abstract screening, 142 were retained for full-text review; double-screening of 20% (n=240) of excluded publications did not identify any missed publications. Forty-three reviewed publications met the inclusion criteria, with most studies excluded as duplicate publication of results (including literature reviews). Twenty additional publications were identified through reference lists of included articles and 29 literature reviews.

In total, 63 publications referring to 61 studies identified in the systematic and manual searches were included in this review. Two studies published initial^20 21^ and follow-up outcomes^22 23^ in separate articles; we report short- and long-term outcomes as a single study.

### Study characteristics and intervention typology

Characteristics of included studies are summarised in online supplementary appendix 2, table 1. Approximately half of the included studies were conducted in the WHO Americas region (n=29), including 22 in the USA. Around half of studies were RCTs (n=29 individual and n=4 cluster RCTs), followed by uncontrolled pre-post studies (n=14) and non-randomised observational studies (n=9). Four studies were described by their authors as having a quasi-experimental design.^24–27^

10.1136/bmjsrh-2019-200377.supp2 Supplementary data

Table 1 summarises the different types of counselling interventions in included studies according to target population. Fouteen of the 61 included studies targeted adolescents or young women (including three each postpartum and postabortion).

**Table 1:** Mapping of study interventions (reference numbers) across target populations in included studies (n=61)

Type of counselling intervention studies	Target population						
	Women choosing method	Women initiating method	All FP service users	Women undergoing abortion	Postpartum women	Non-FP service users	Community-based
Digital decision-making tool	21 23 30–32 81 A+YW^28 29^:						
Provider training and paper-based decision-making tool	25 33 34 36	26		60			
Structured counselling on side effects		39–41 92					
Tubal ligation scoring		42					
Provider training in counselling, clinical and/or logistical skills			25 27 44–49	57	71 A+YW^62^:		
Content of counselling					67 68 72		
Provider training + telephone counselling		A+YW^38^:					
Additional provider counselling (including motivational interviewing)				54 58 A+YW^51^:			
Additional video counselling				A+YW^53^:			
Telephone-based automated messages or counselling (additional or in lieu of face-to-face counselling)		A+YW^37^:		55 56			
Leaflet or video vs face-to-face counselling					69 70		
Individual vs group counselling (with/out alternative timing)				20 22 A+YW^52^:	66		
Number/timing of counselling sessions					63–65 A+YW^61 93^:		
Patient coaching			50				
Peer counselling							A+YW^80^:
Systematic contraceptive counselling						74–76 A+YW^73^:	
Husband or couples counselling		43		59			77 78 A+YW^79^:

Numbers refer to study references.

A+YW, adolescents and young women (author definitions, upper limit <30 years old); FP, family planning.

The most commonly reported outcomes were contraceptive use among all women (n=32), continuation among women who had initiated a method (n=23) and method uptake among women not using at the time of counselling (n=15). Only one study reported information on contraceptive switching at the time of counselling. Nineteen of the 61 studies reported satisfaction outcomes, including eight on method satisfaction and 13 on satisfaction with counselling and/or services. Only 25 of the 61 studies (41%) included more than one of the six outcomes assessed in this review.

### Association with outcomes

Online supplementary table 2 describes the 61 included studies; the intervention effects on outcomes of interest is summarised in table 2.

**Table 2:** Summary of intervention effects

Intervention		RCT	Adolescents/young women	Use	Uptake	Continuation	Switching	Satisfaction – method	Satisfaction – services
Interventions targeting women choosing a contraceptive method									
Digital decision-making tools	Chewning 1999^28^		✓	Two sites: 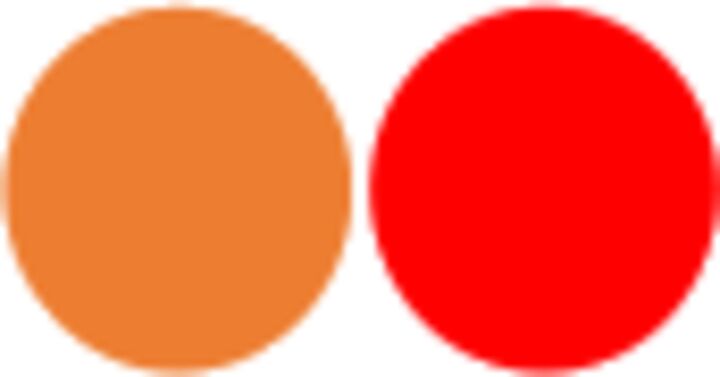		Two sites: 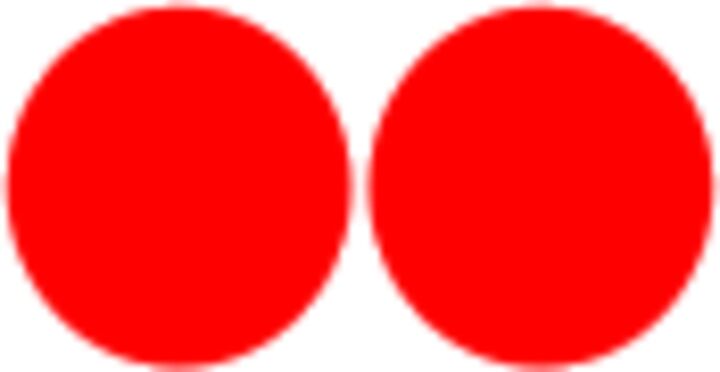			
	Dehlendorf 2017^81^							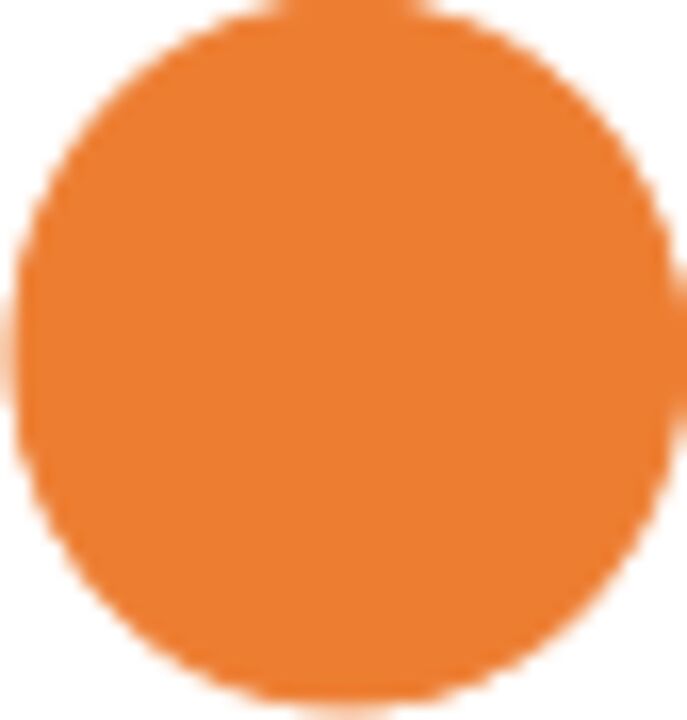	
	Garbers 2012^21^ 2012^23^	✓		Two trial arms: 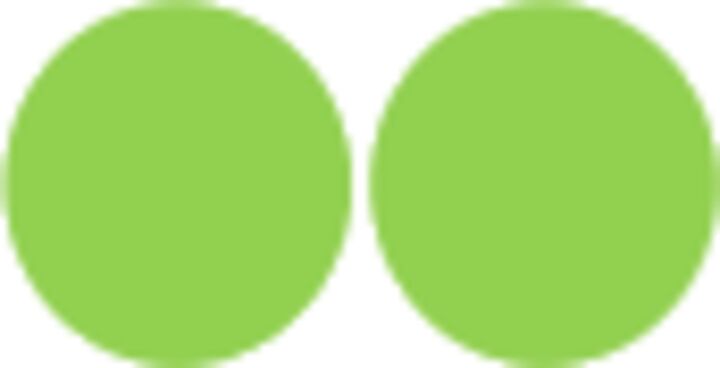		Two trial arms: 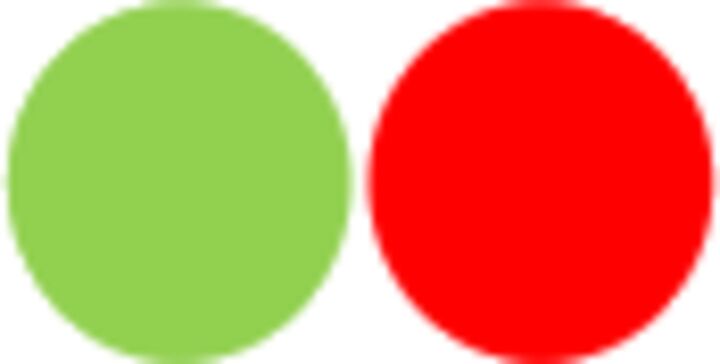			
	Hebert 2018 ^33^	✓	✓	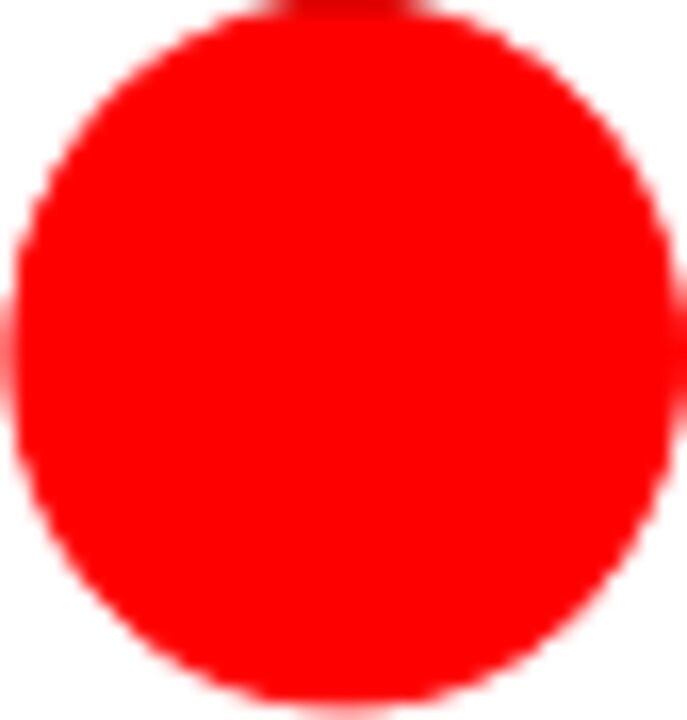	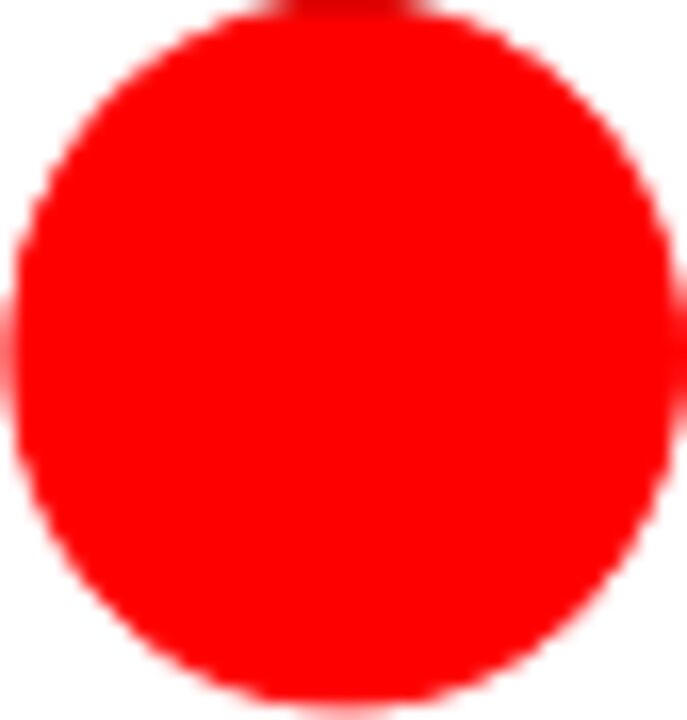				
	Kofinas 2014^29^	✓							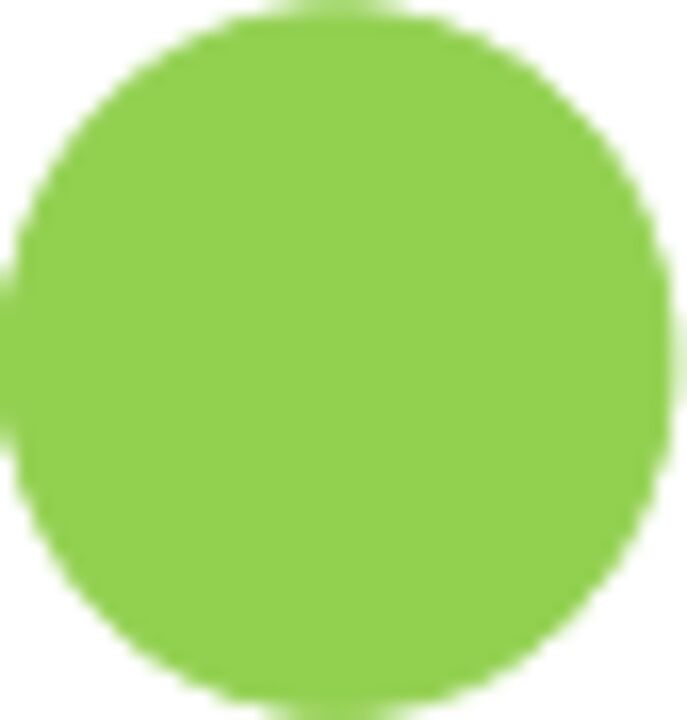
	Koo 2017^30^				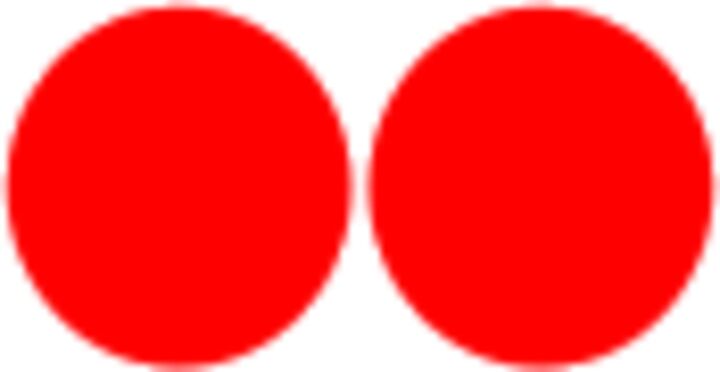				
	Sridhar 2015^31^	✓			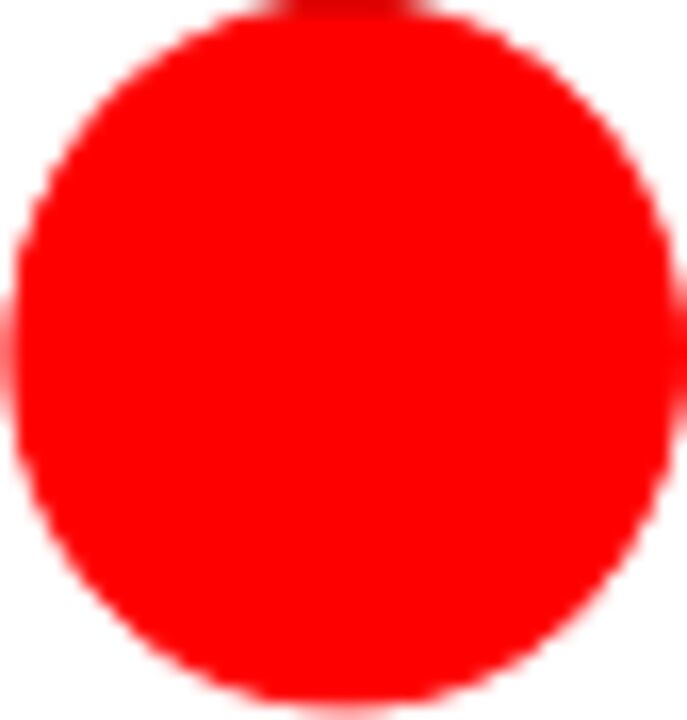				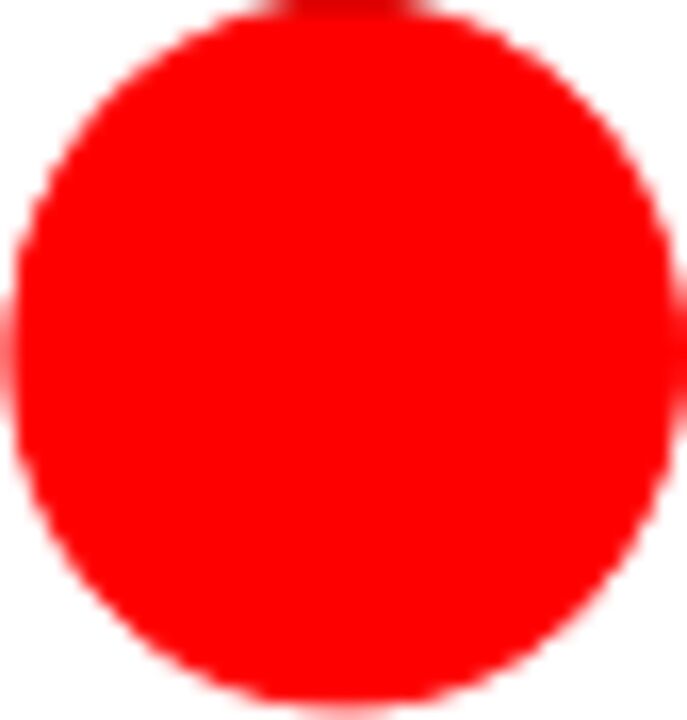
Provider training + paper-based decision-making tools	Farrokh-Eslamlou 2014^34^			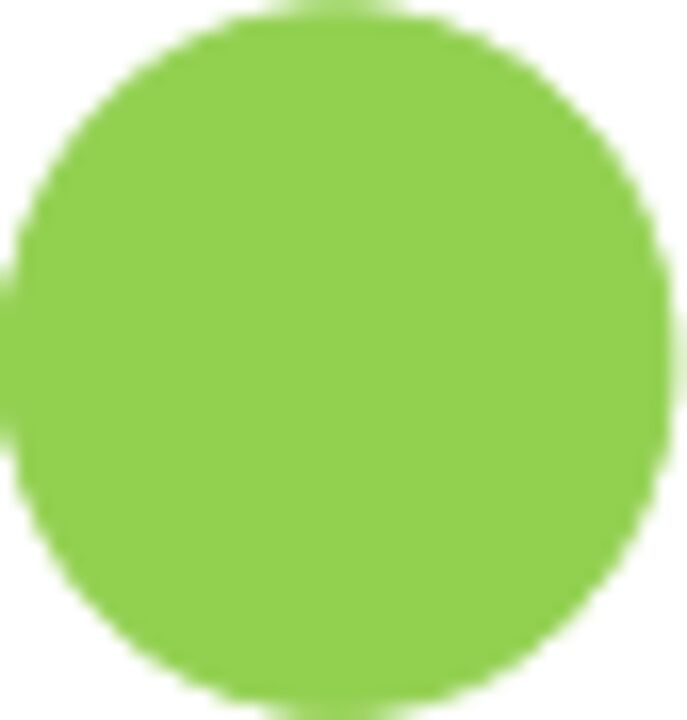					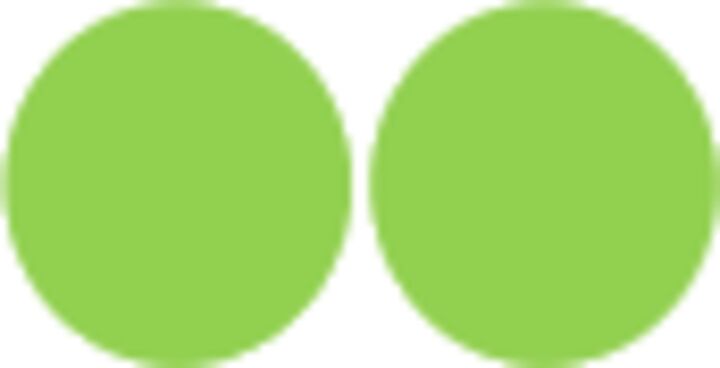
	George 2015^35^				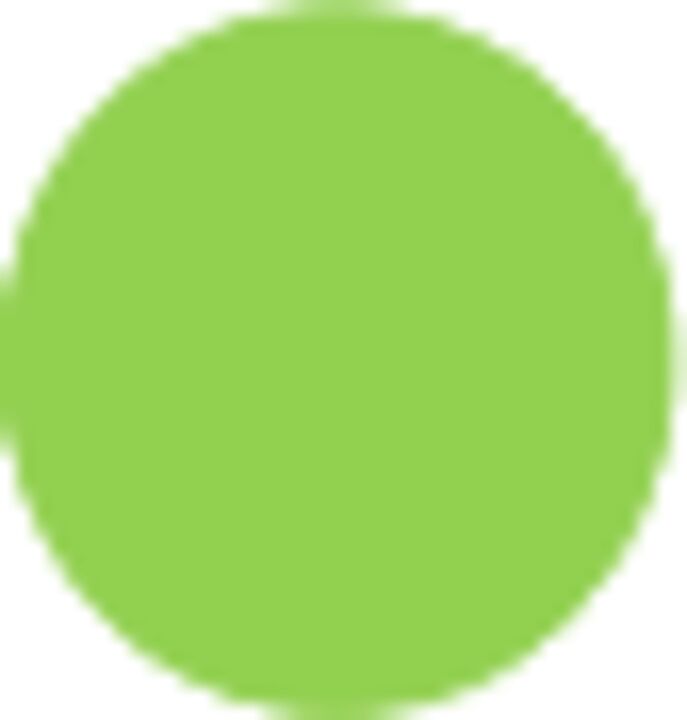				
	León 2003^36^	✓		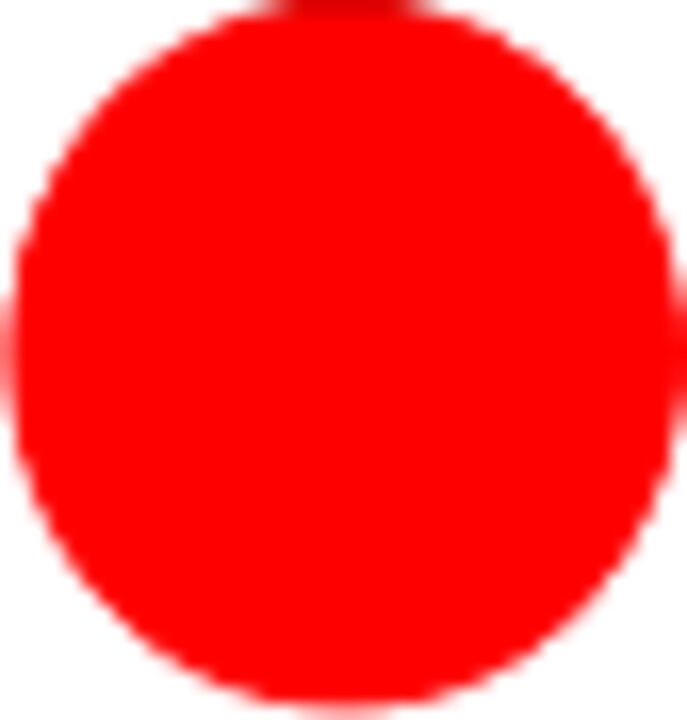		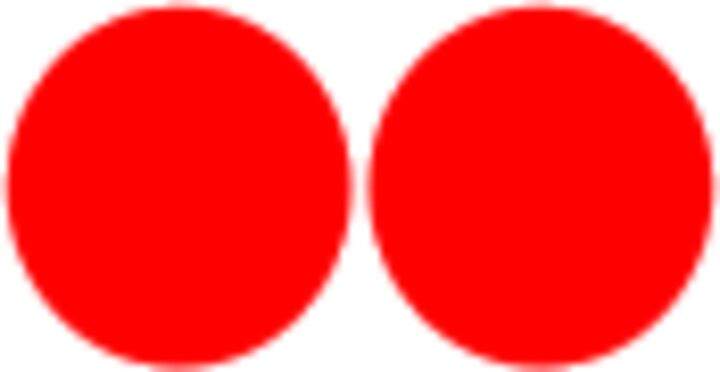			
	Nawar 2004^25^			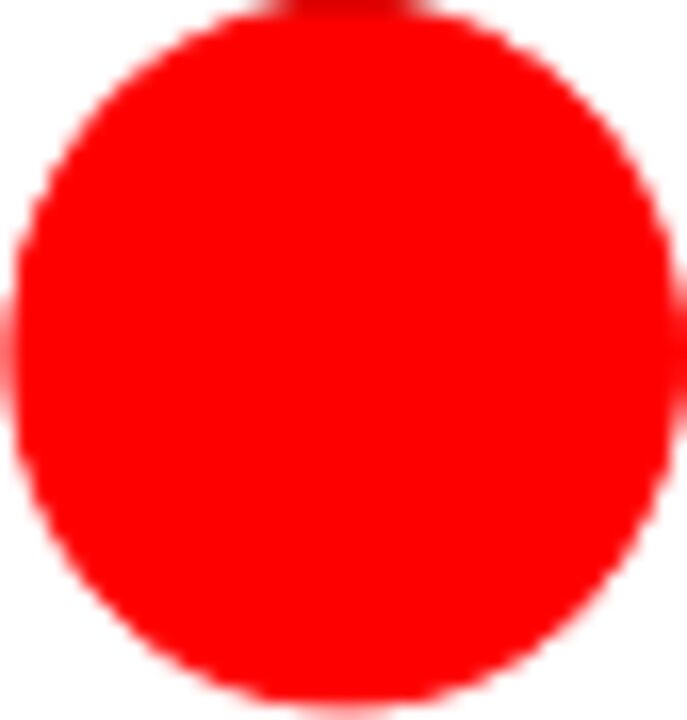		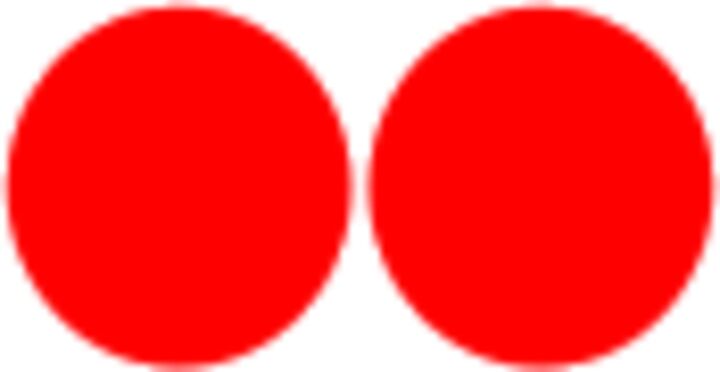			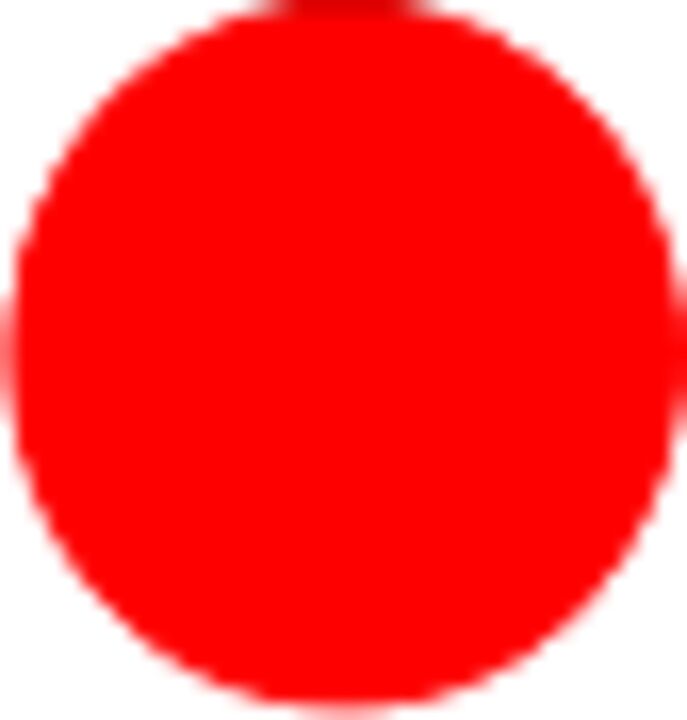
Interventions targeting women initiating or requesting a method									
Structured counselling on side effects	Canto de Cetina 2001^38^	✓				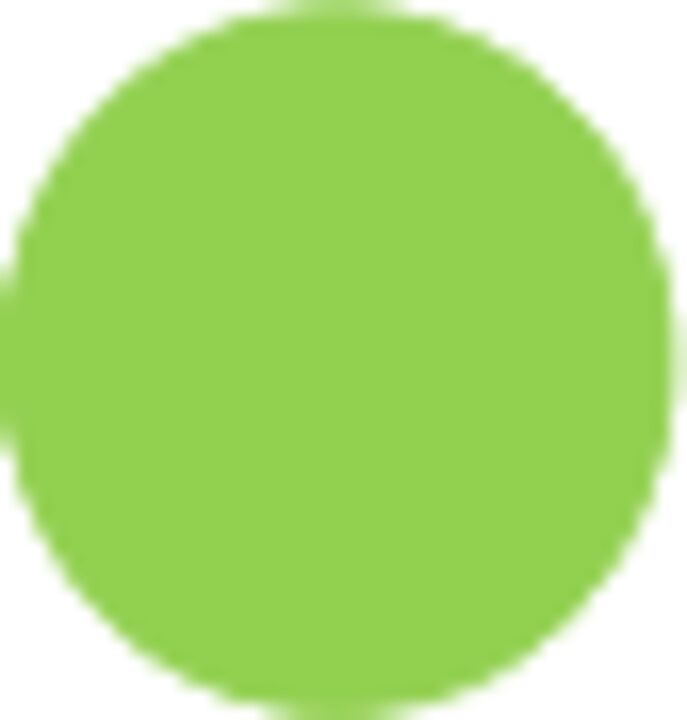			
	Lei 1996^92^					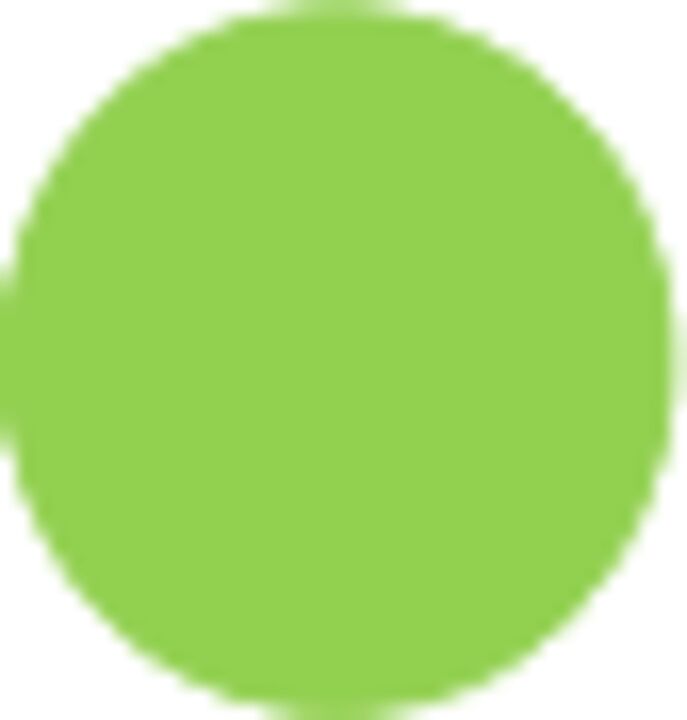			
	Modesto 2014^40^	✓				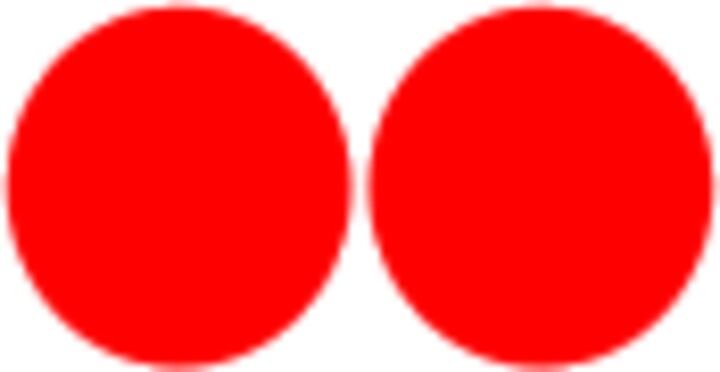			
	Patel 2003^41^					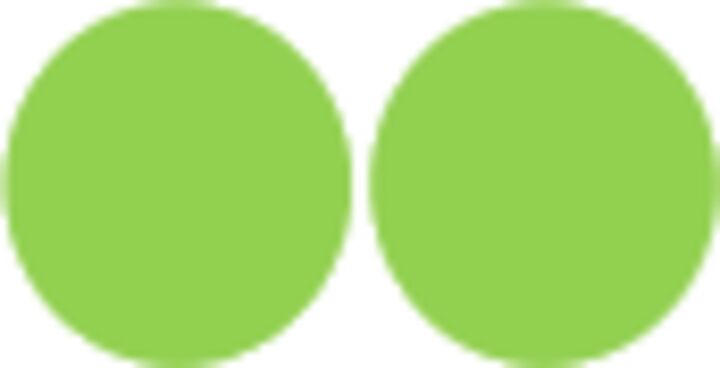			
Tubal ligation scoring	Demir 2006^42^					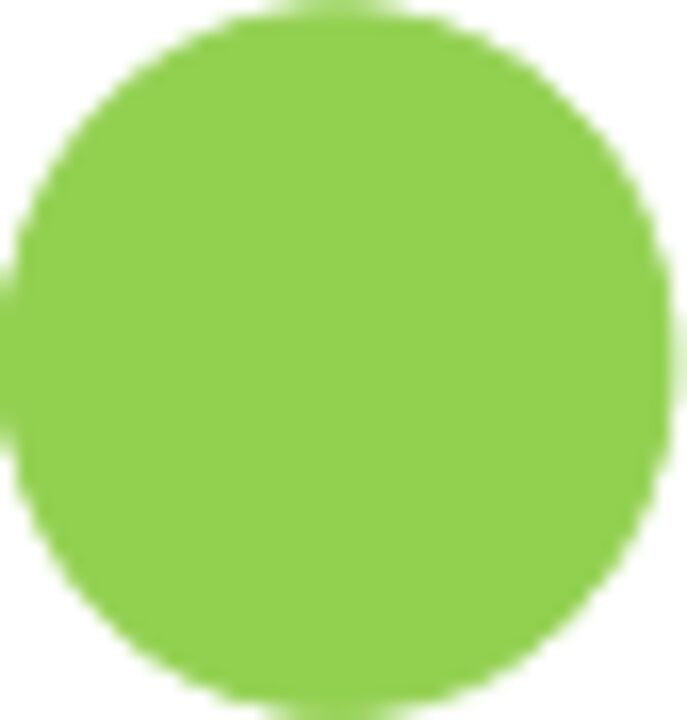			
Paper-based decision-making tool	Chin-Quee 2007^26^					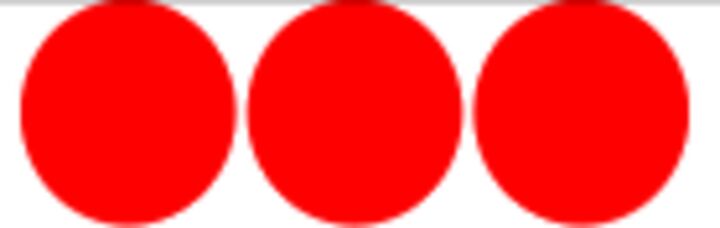			
Provider training + telephone counselling in one arm	Berenson 2012^56^	✓	✓			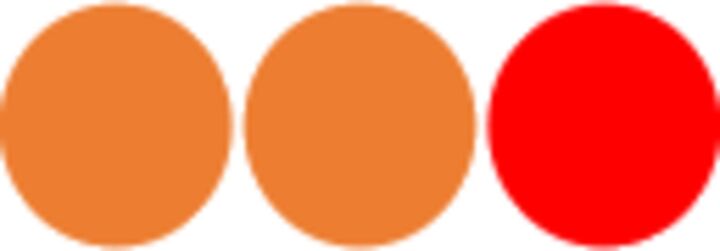		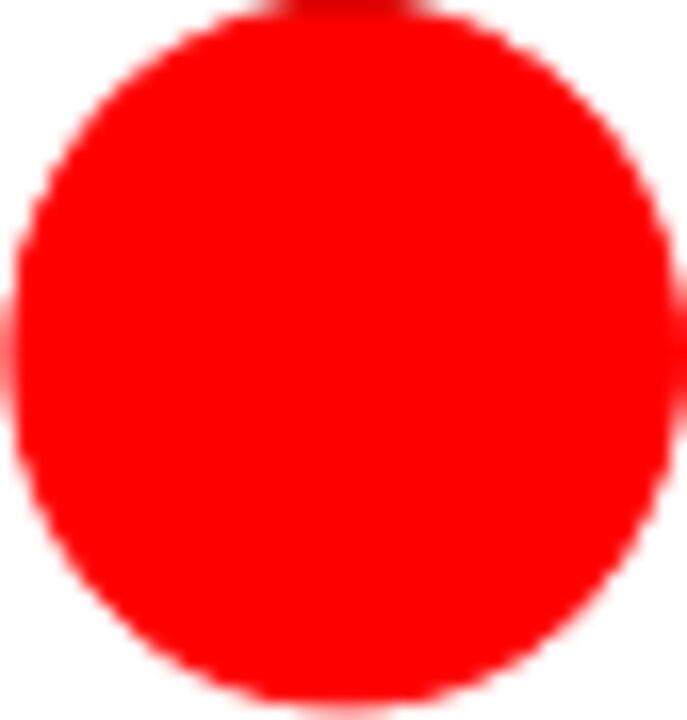	
Educational text messages	Castaño 2012 61	✓	✓			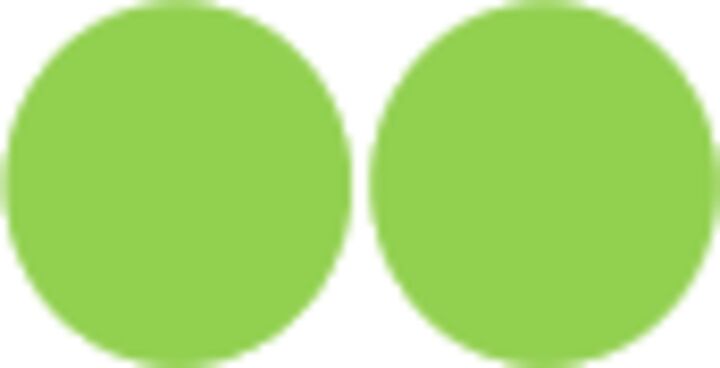			
Husband counselling	Amatya 1994^79^					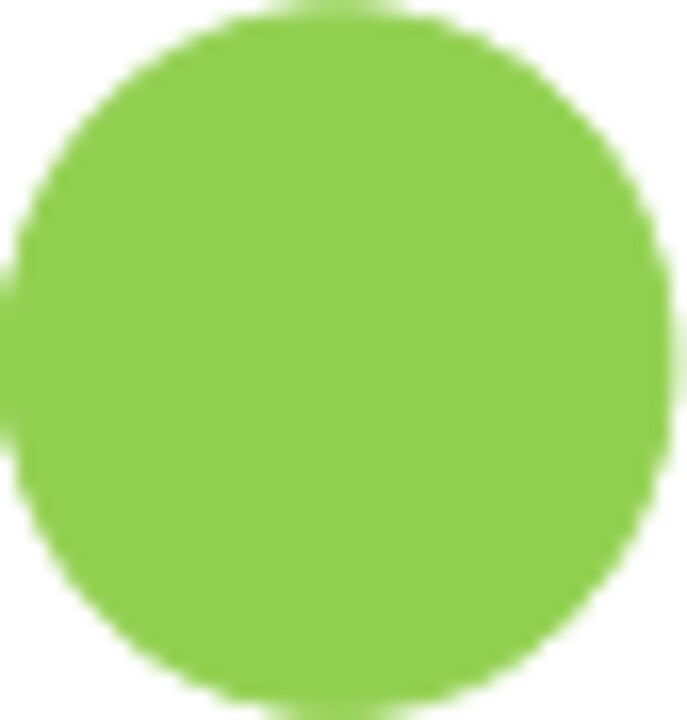		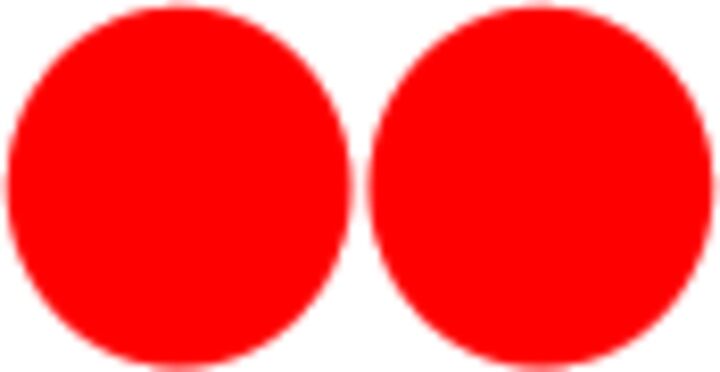	
Interventions to improve FP service quality for all service users									
Provider training in counselling, clinical and/or logistical skills	Gibbs 2016^43^	✓	✓		18–25 years old: 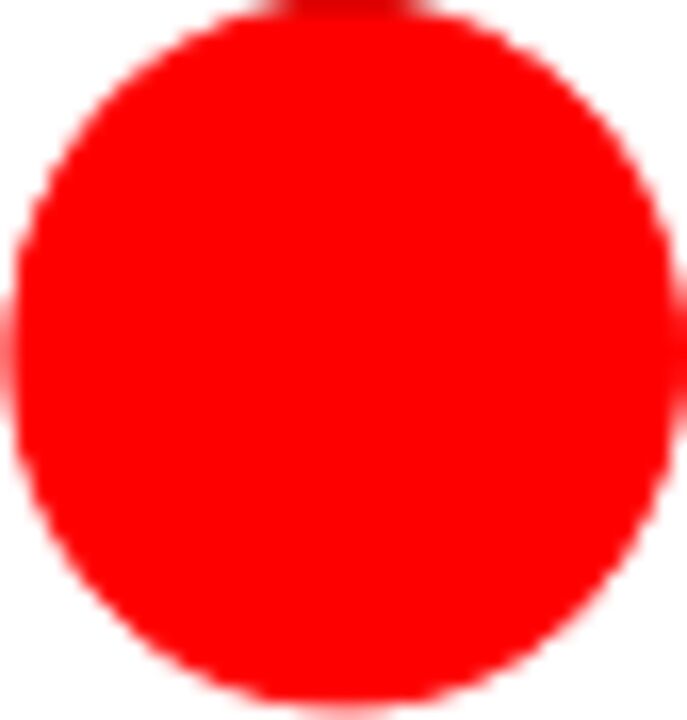 18–19 years old: 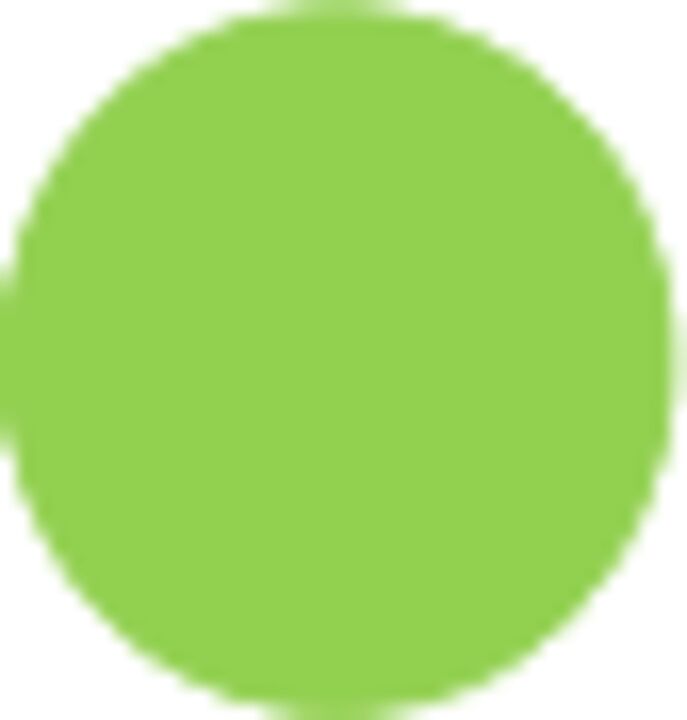				
	Jain 2012^24^			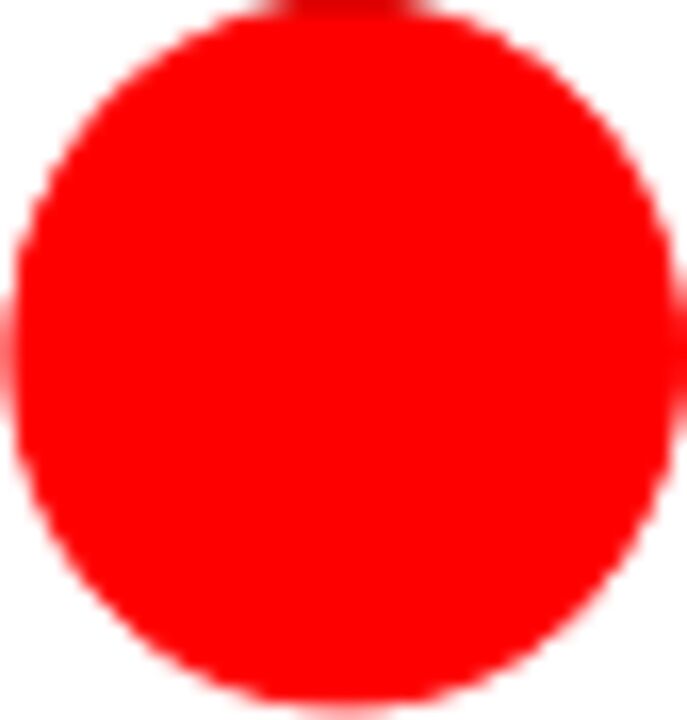					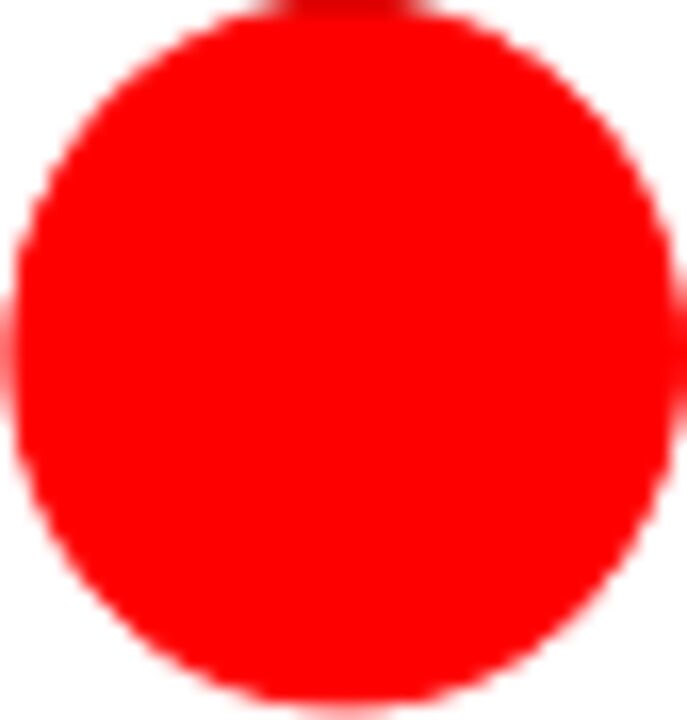
	Kim 1992^44^					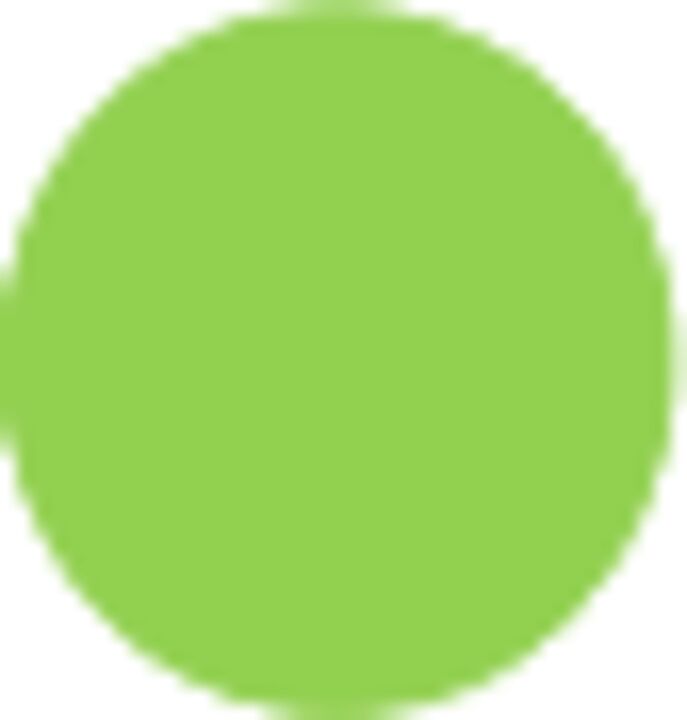			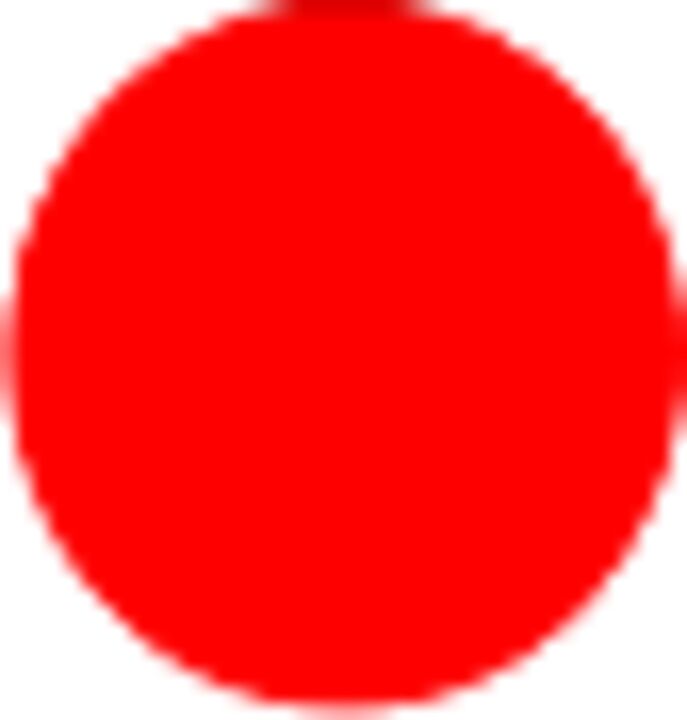
	León 2003^45^			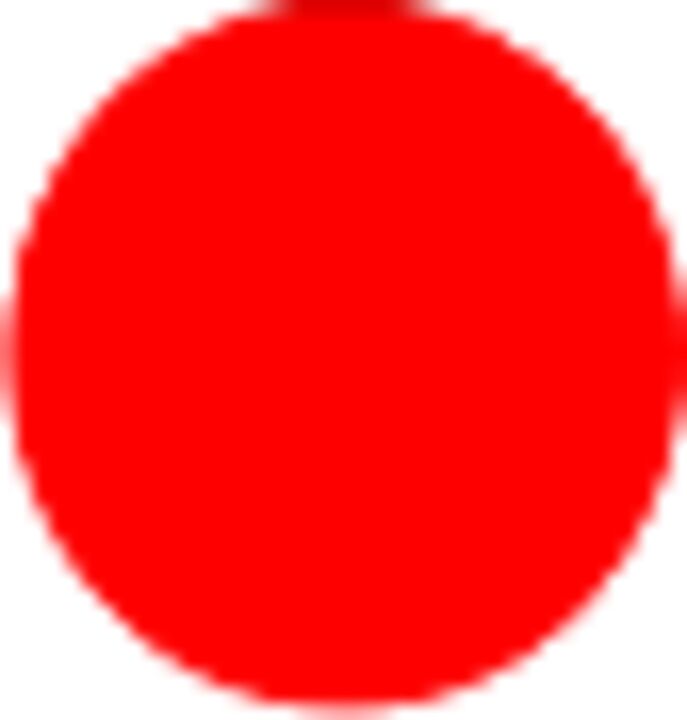		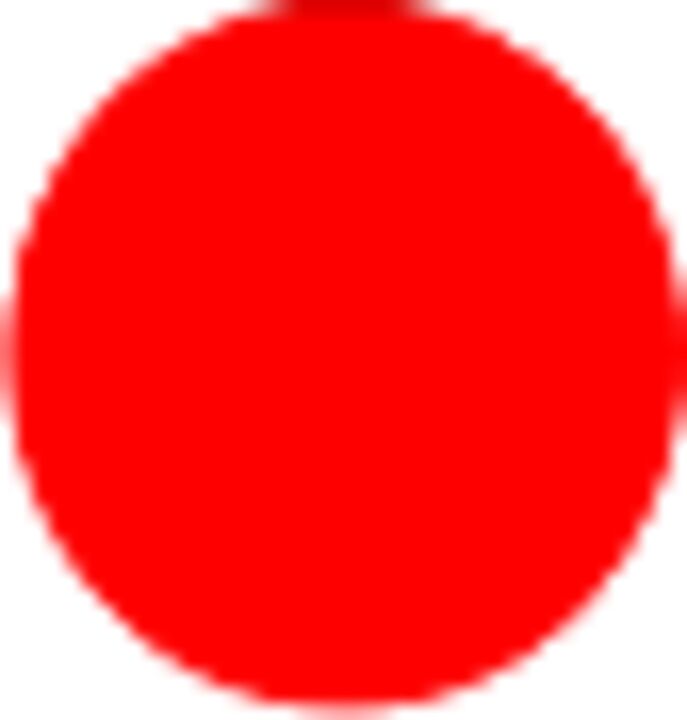			
	Madden 2013^46^				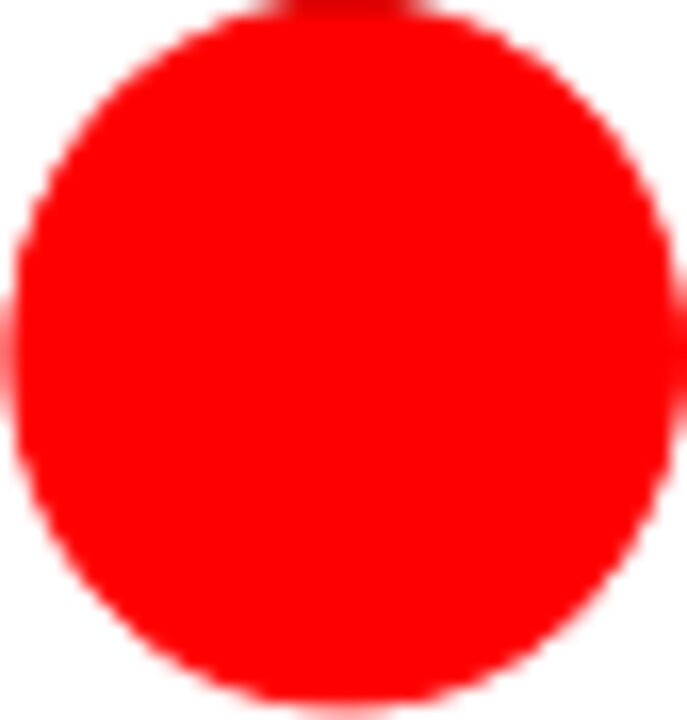				
	Sanogo 2003^47^			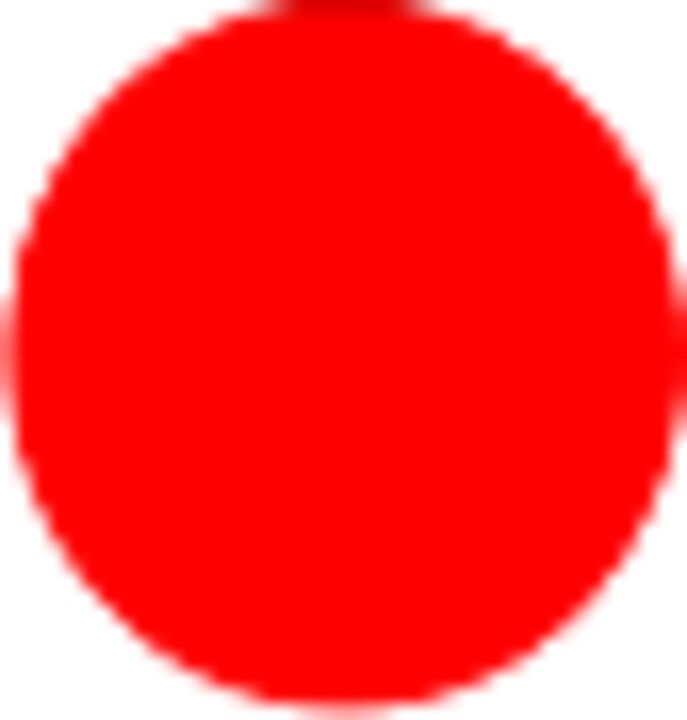					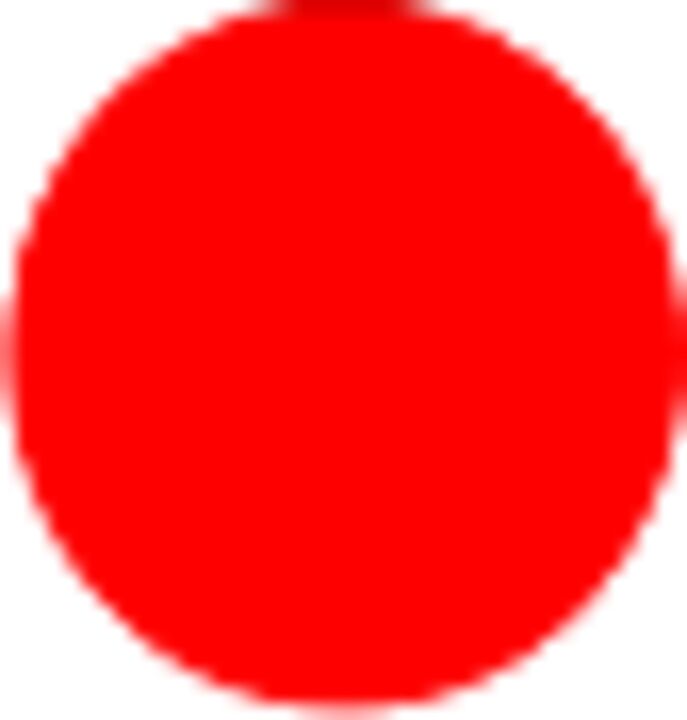
	Sapkota 2017^48^				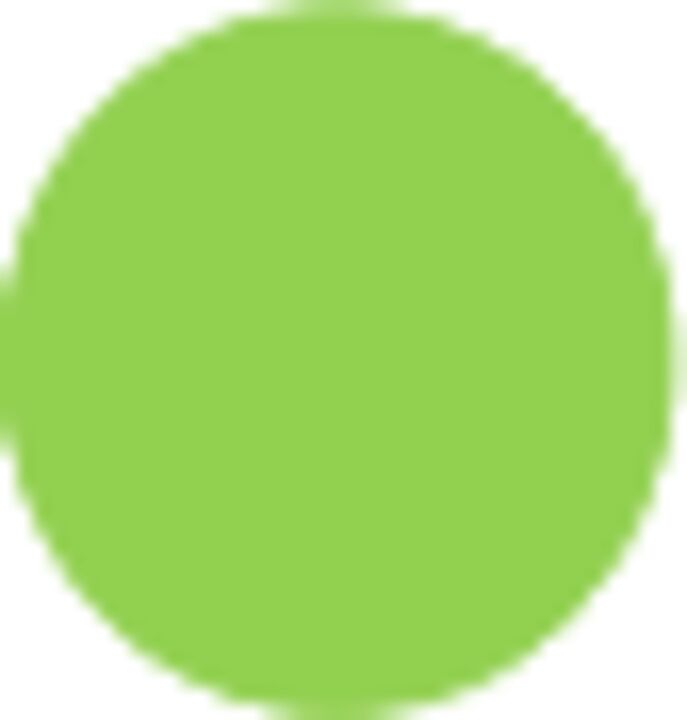				
	Wu 2003^49^								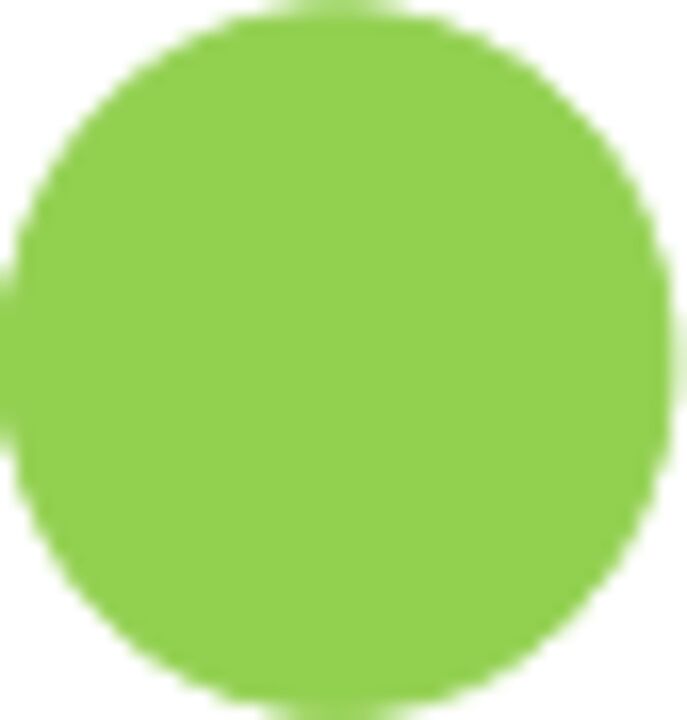
Patient coaching	Kim 2003^50^	✓				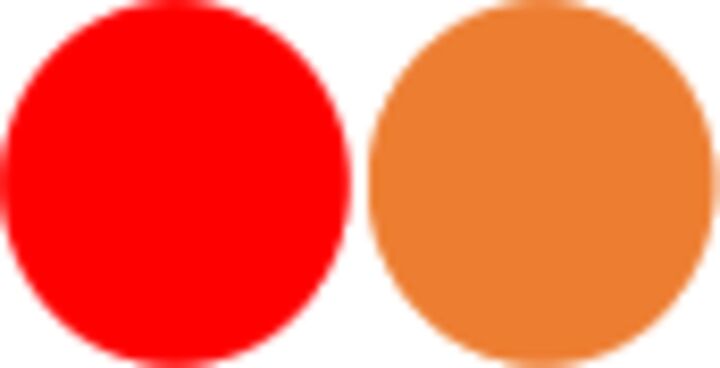			
Interventions targeting women undergoing abortion									
Additional counselling	Bender 2004^58^	✓		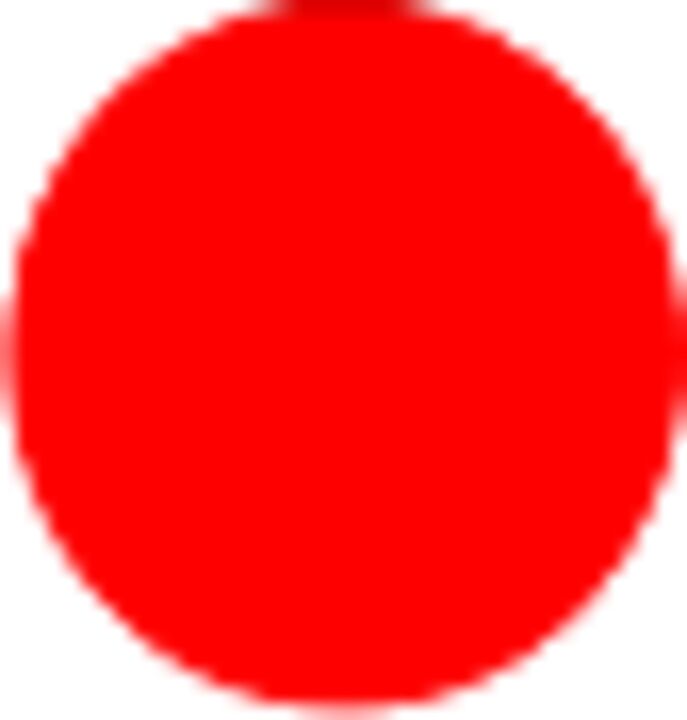					
	Davidson 2015^53^	✓	✓	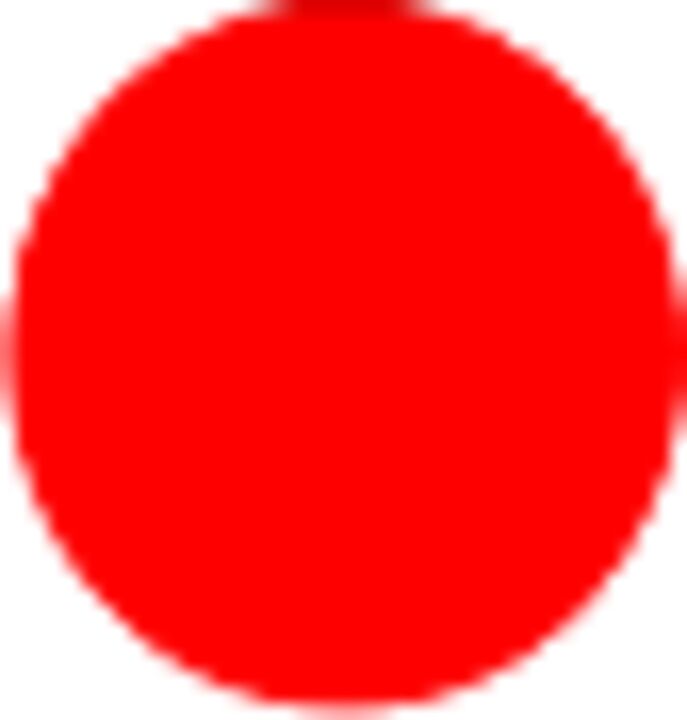	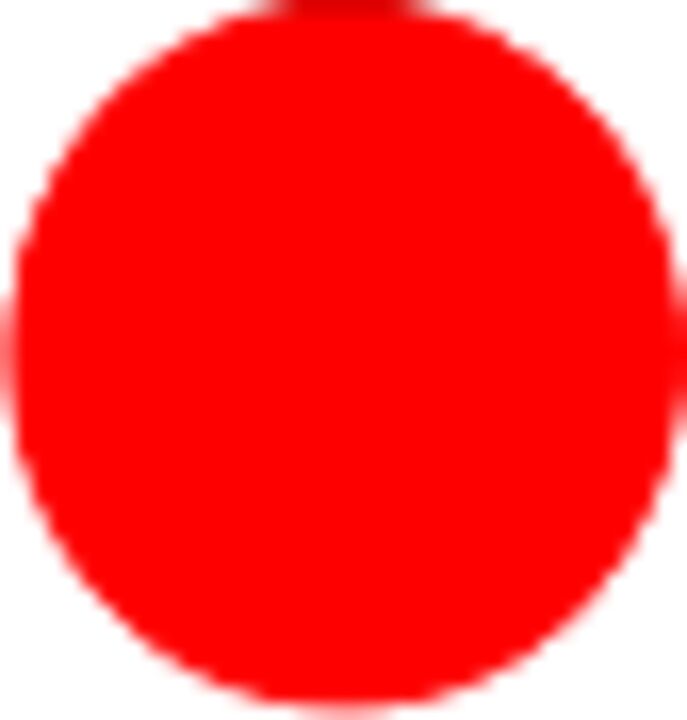			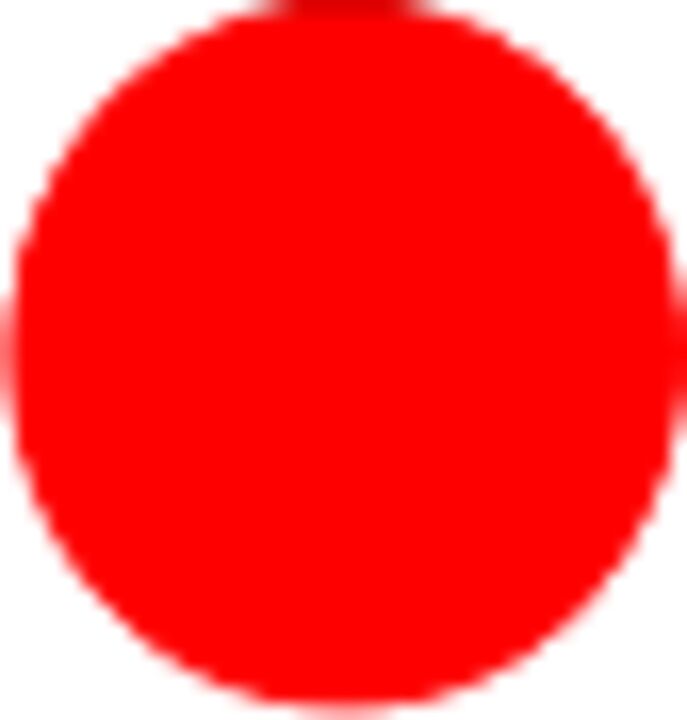	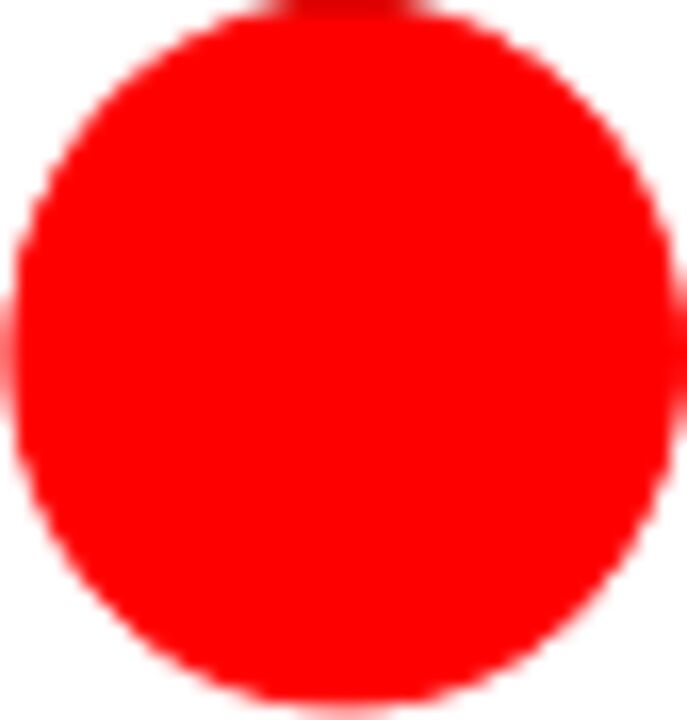
	Schunmann 2006^54^	✓		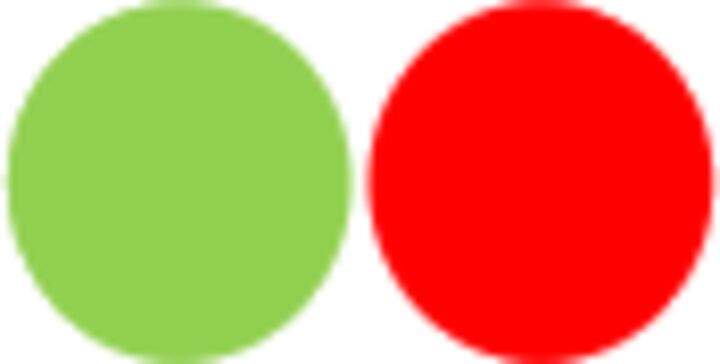	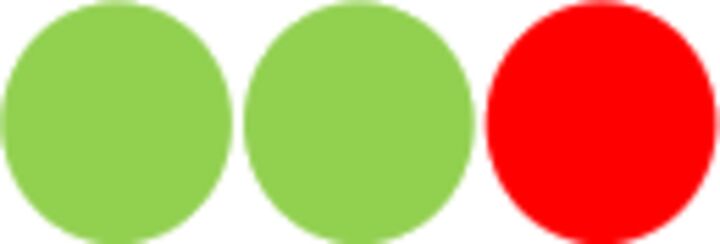	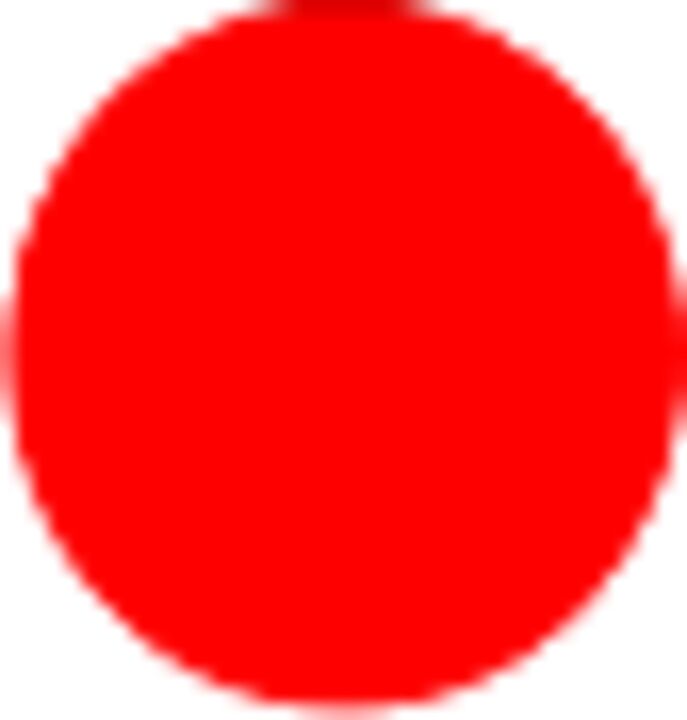			
	Smith 2015^55^	✓		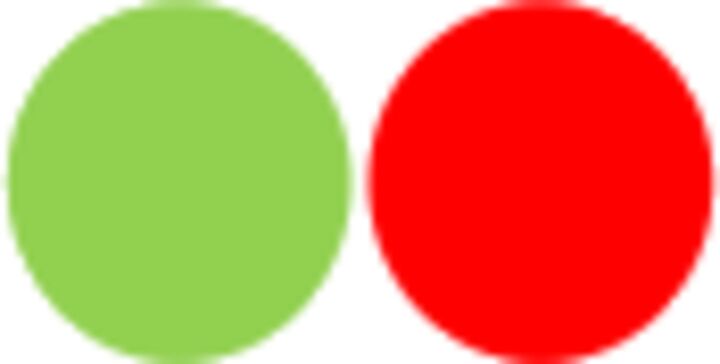		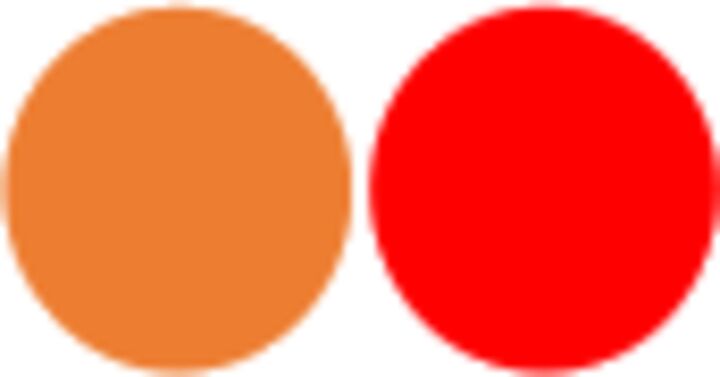			
	Whitaker 2016^51^	✓	✓	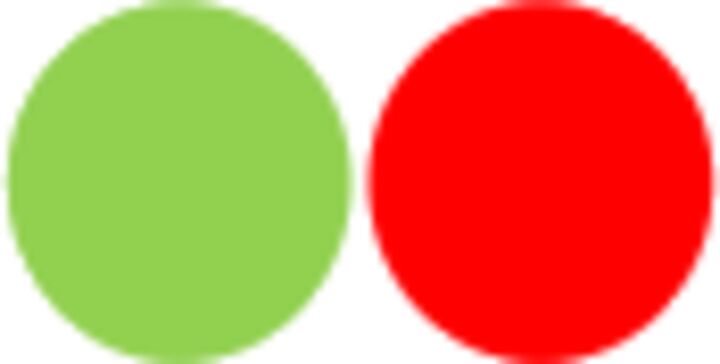	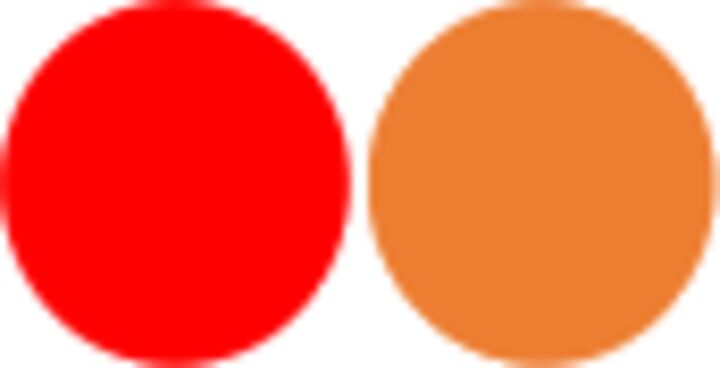			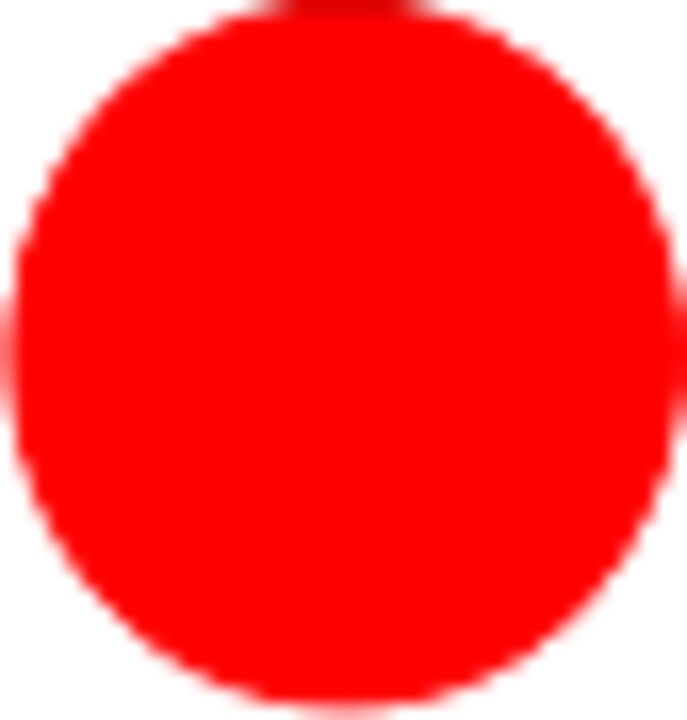	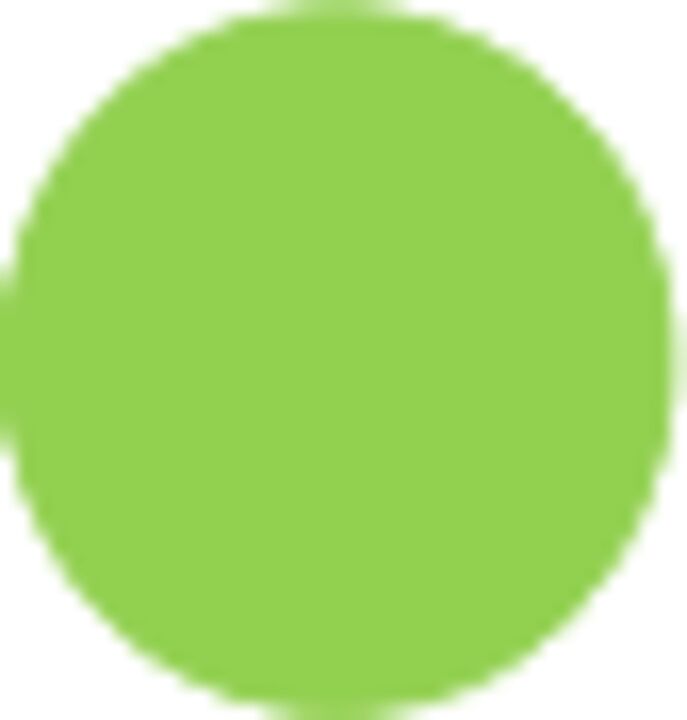
	Zhu 2009^52^	✓	✓	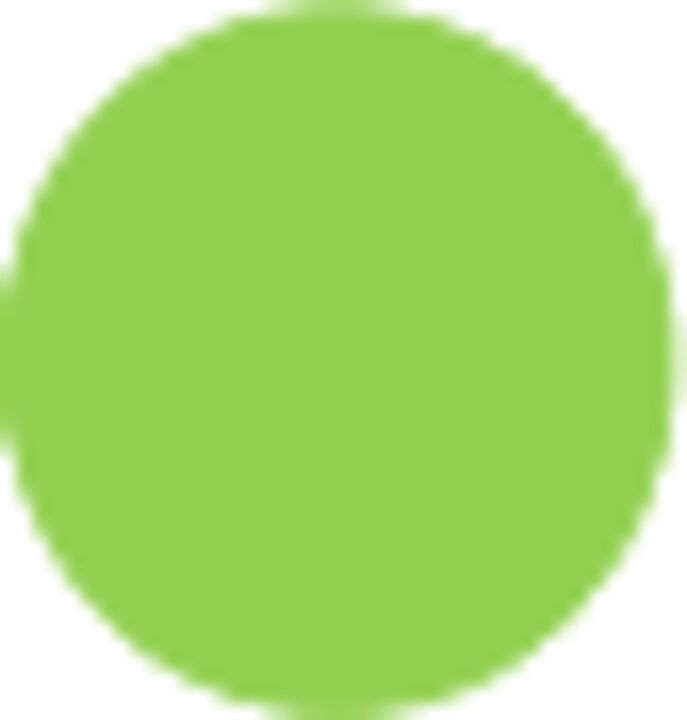					
Mode of counselling	Ferreira 2011^20^ 2015^22^	✓		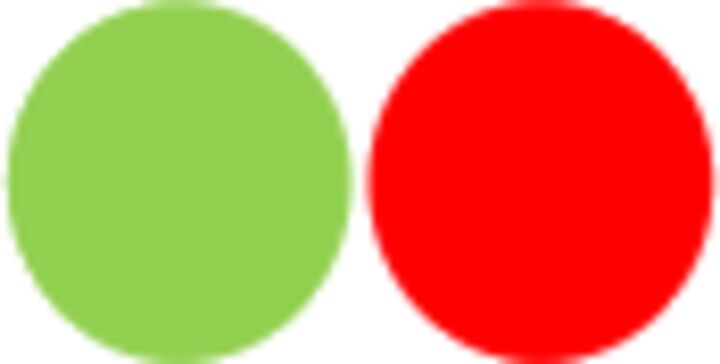				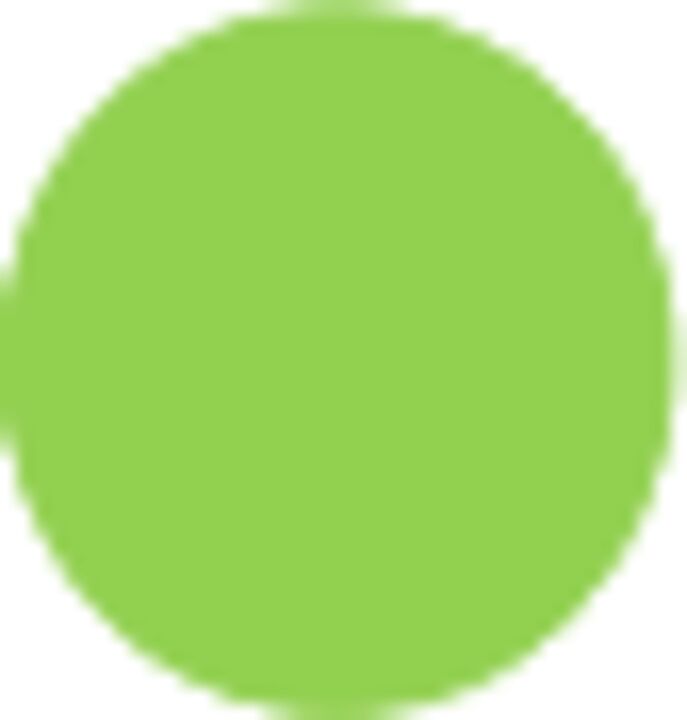	
	Lohr 2018^56^				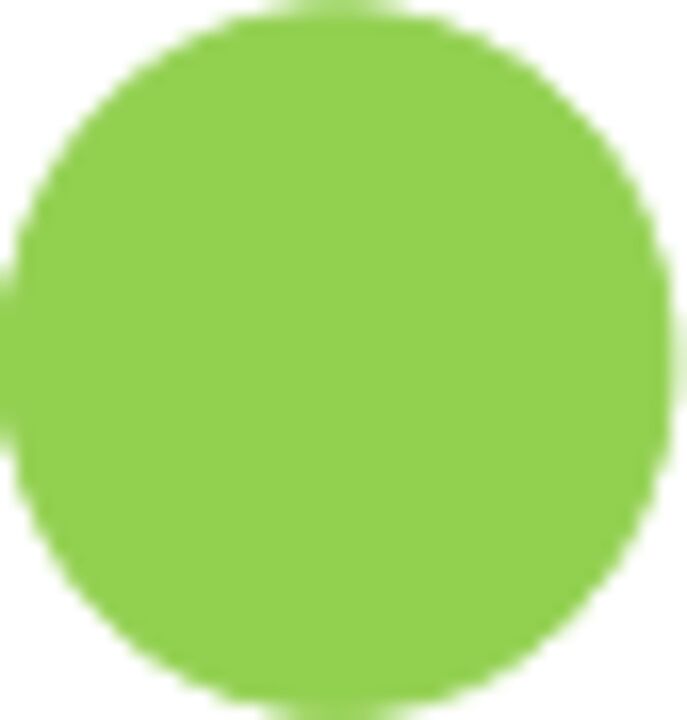				
Content	Langston 2010^60^	✓				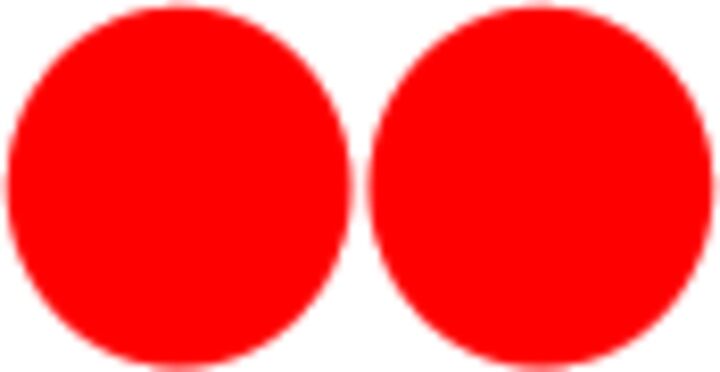			
	Savelieva 2003^57^			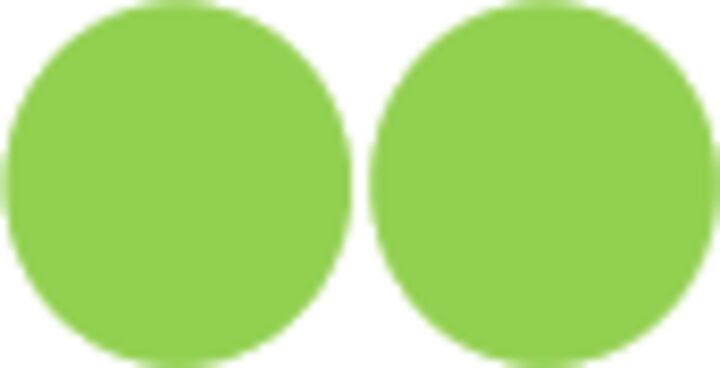					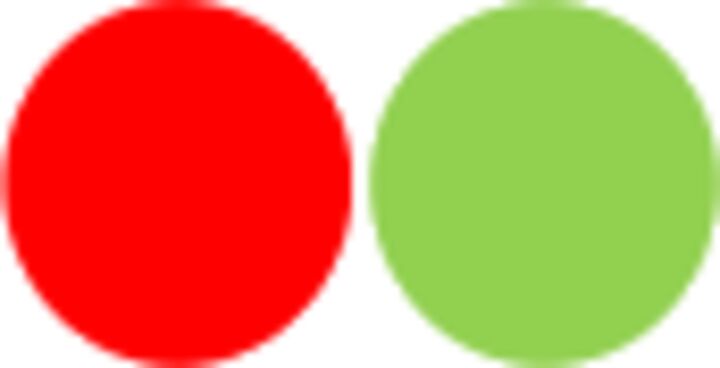
Husband counselling	Abdel-Tawab 1997^59^	✓		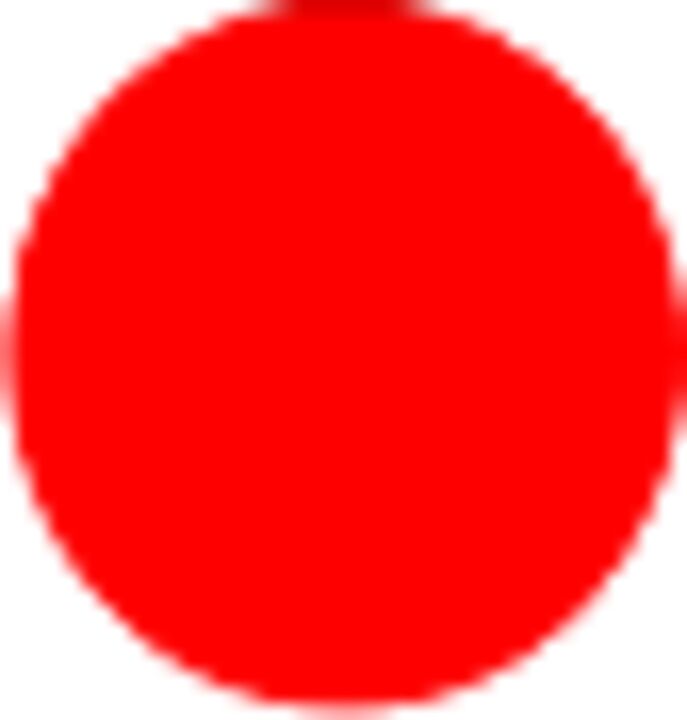					
Interventions targeting postpartum women									
Number/timing of sessions	Adanikin 2013^64^	✓		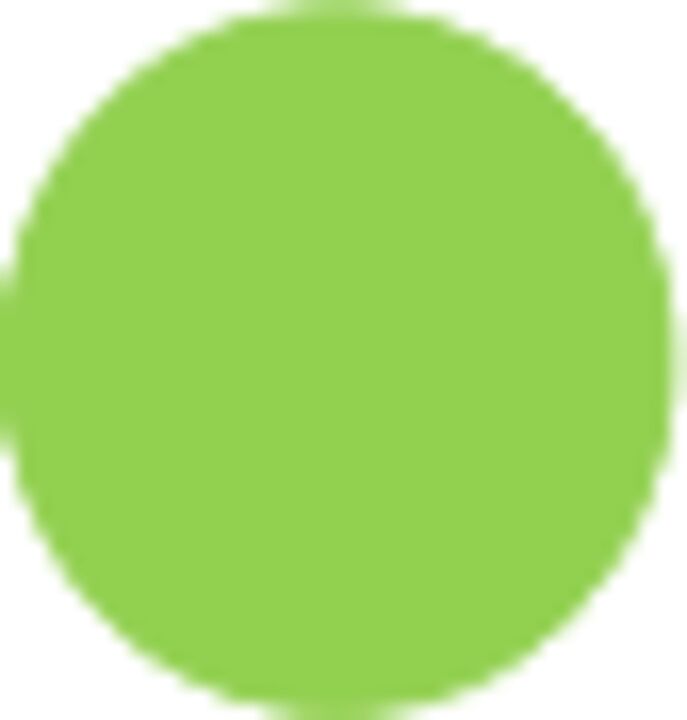					
	Bolam 1998^63^	✓		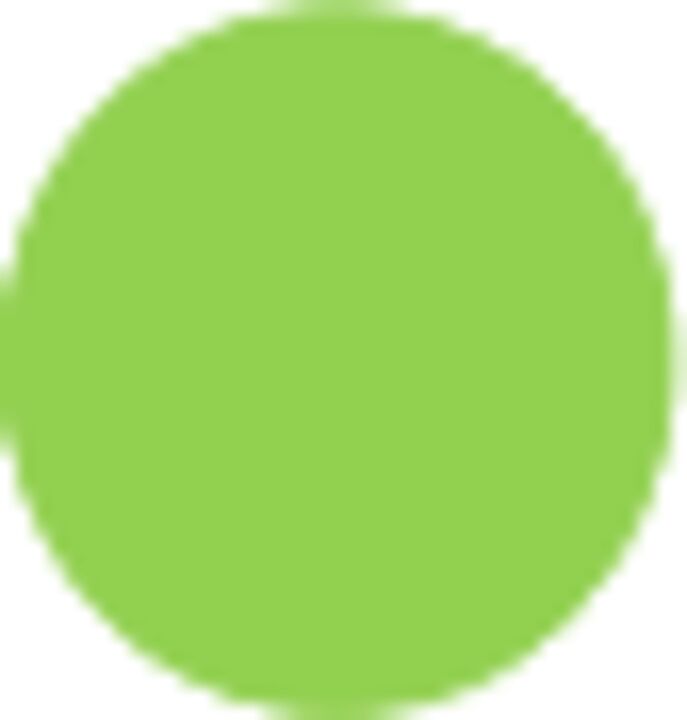					
	Gilliam 2004^93^	✓	✓			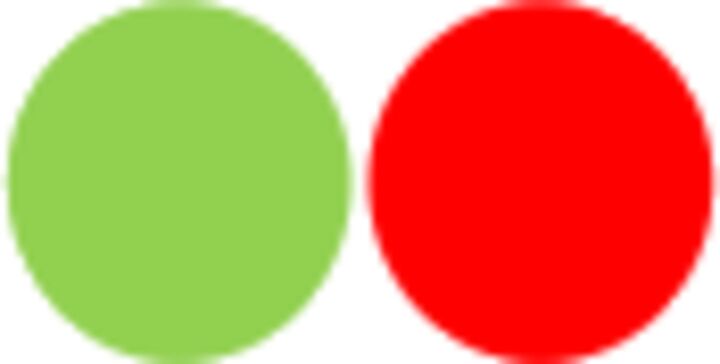			
	Kaewkiattikun 2017^61^	✓	✓	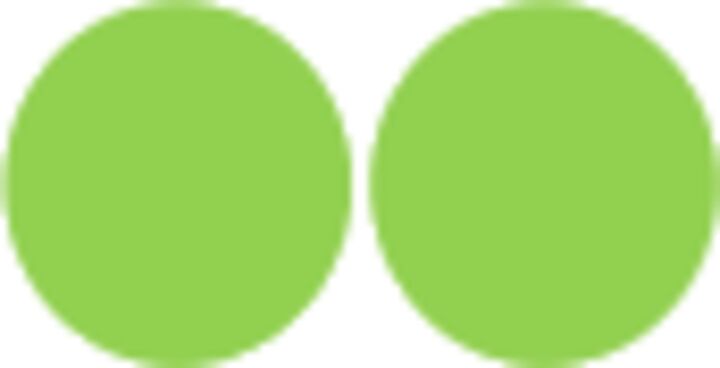					
	Vural 2016^65^			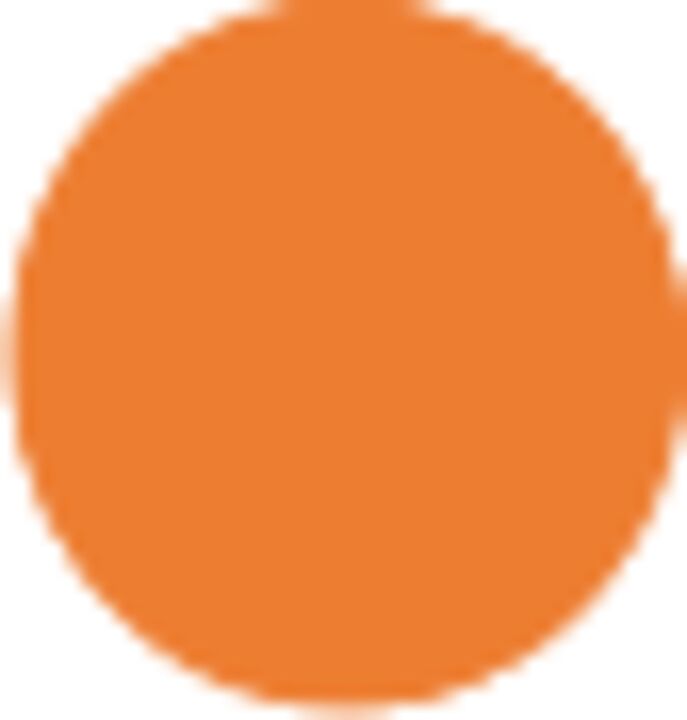					
Timing/mode of counselling	Smith 2002^66^	✓		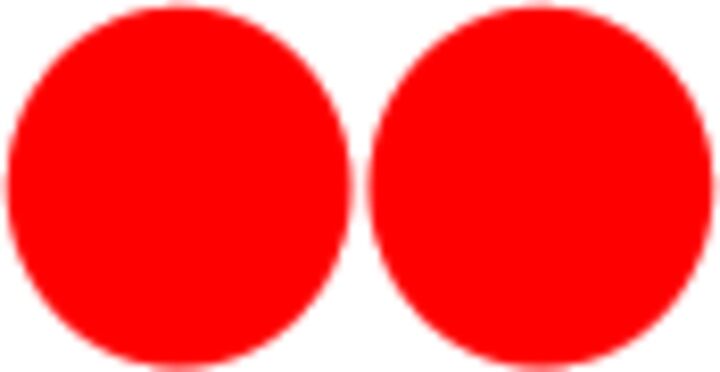					
Mode of counselling	Akman 2010^70^	✓		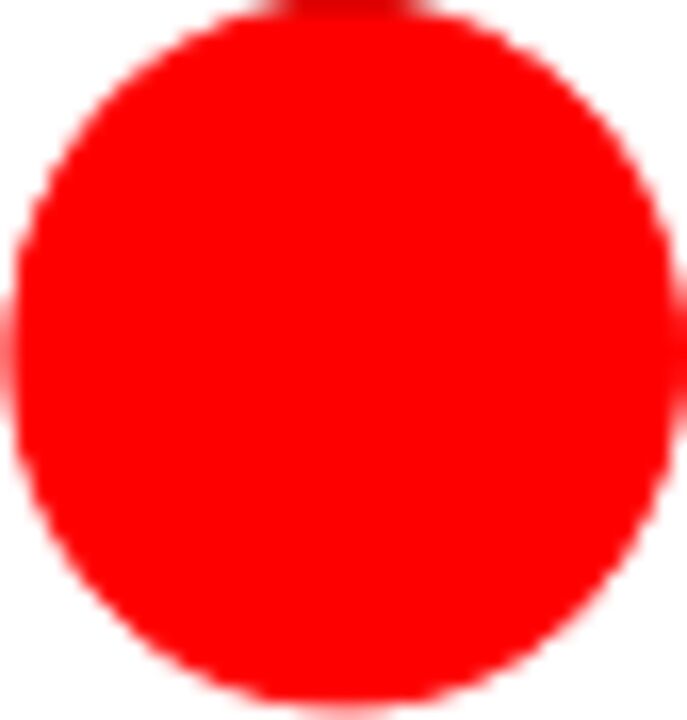					
	Proctor 2006^69^	✓							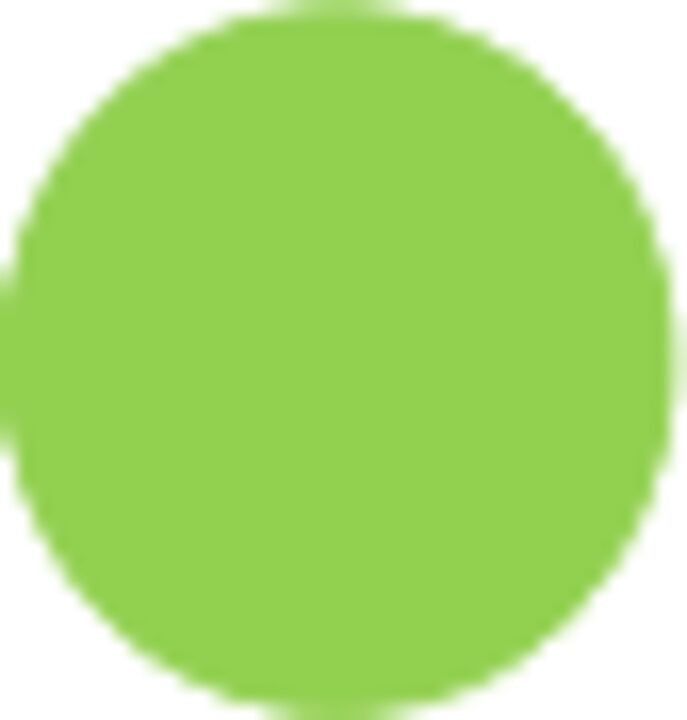
Content	Fatima 2018^71^				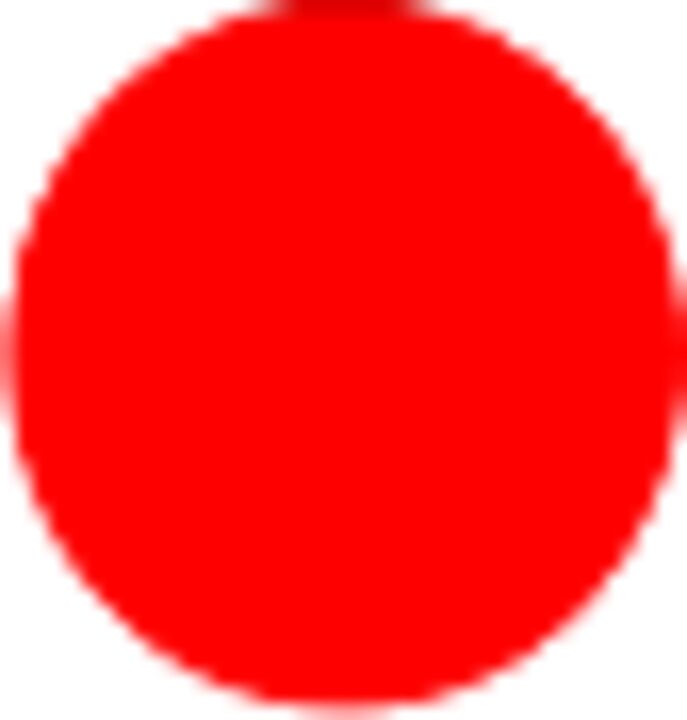	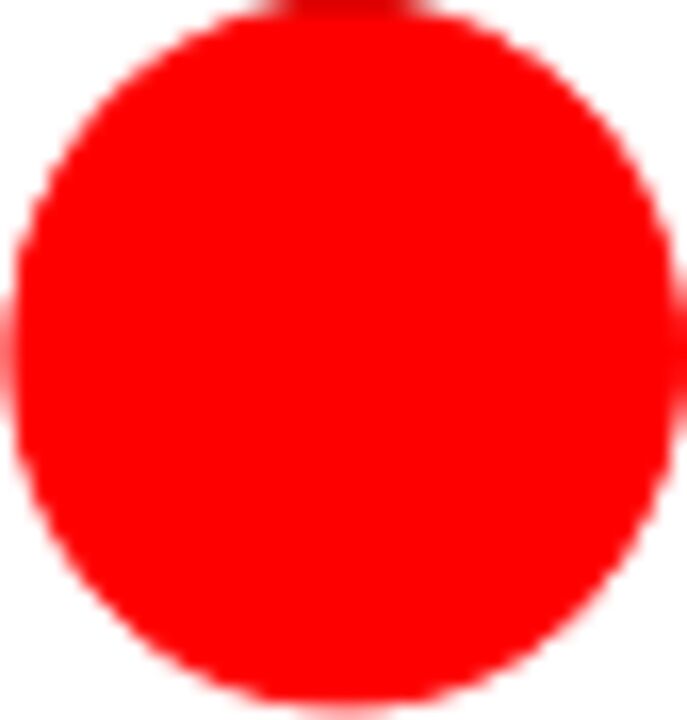			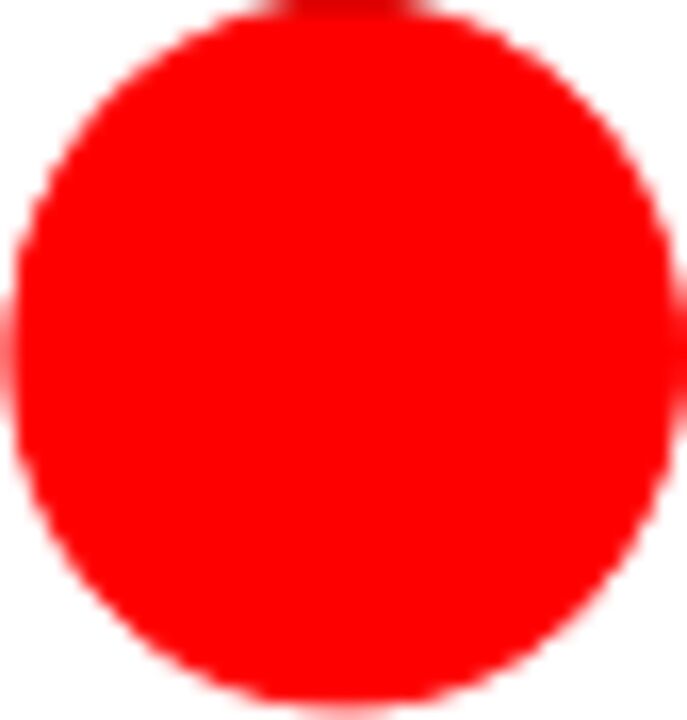
	Hardy 1998^68^			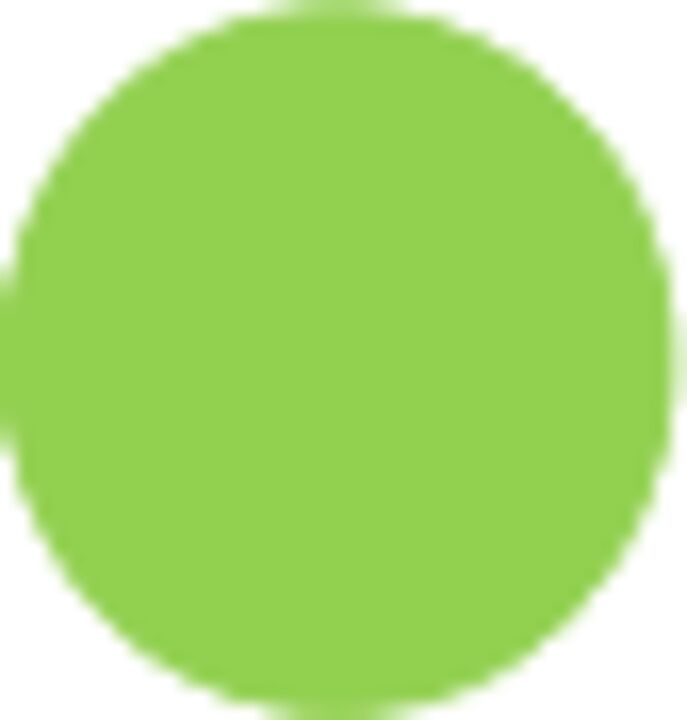					
	Ndegwa 2014^72^	✓			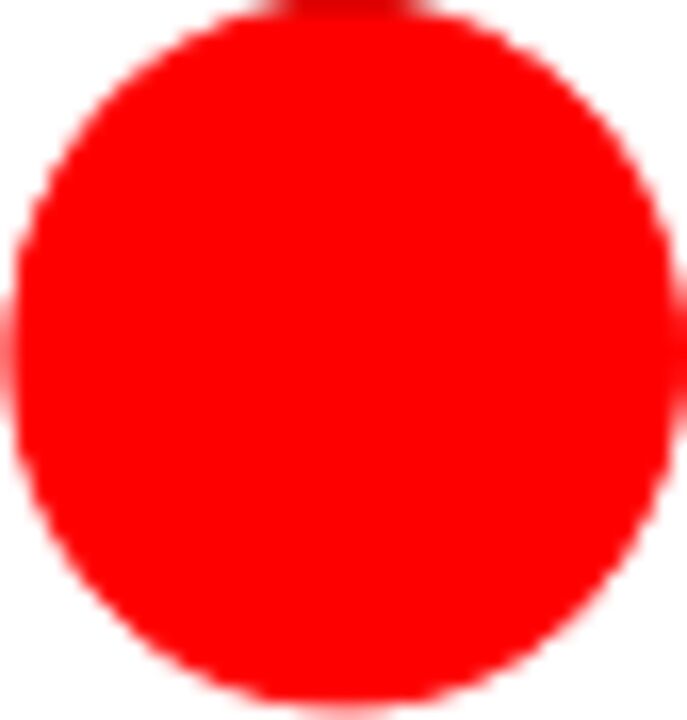	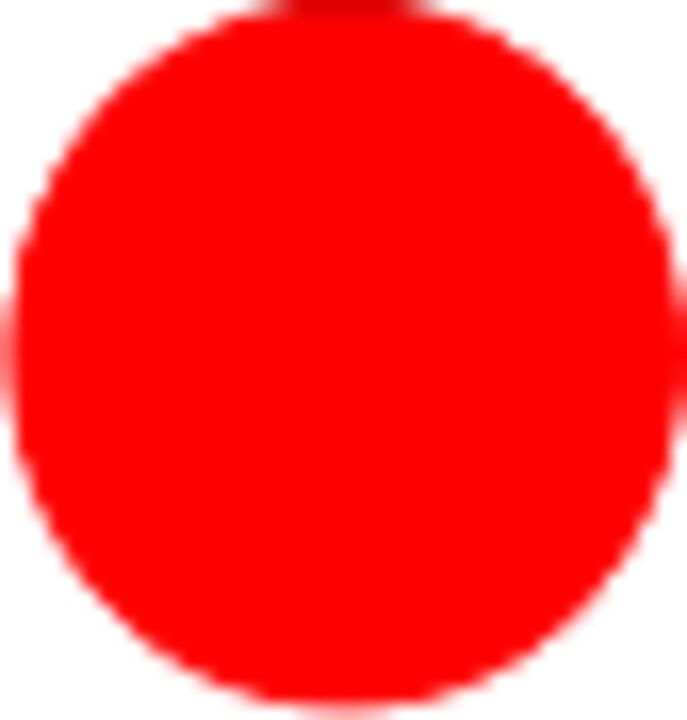			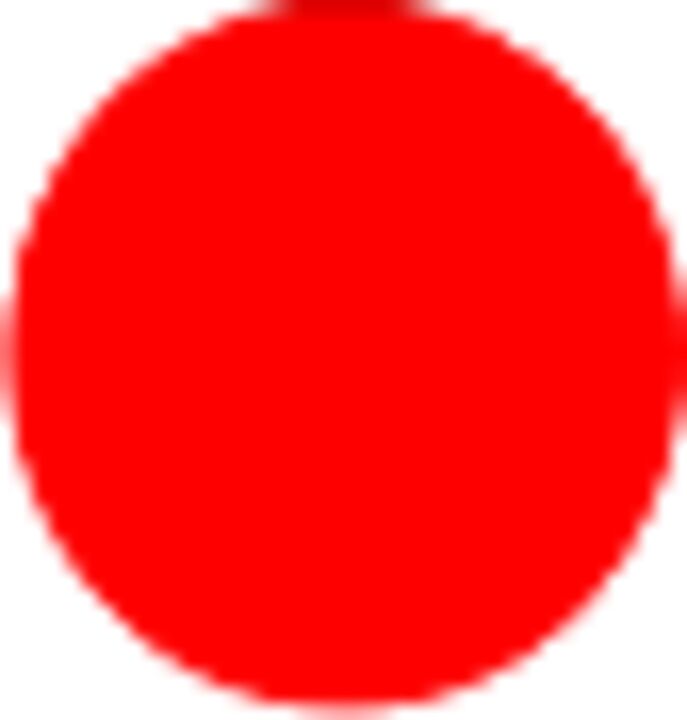
	Tomlin 2017^62^		✓	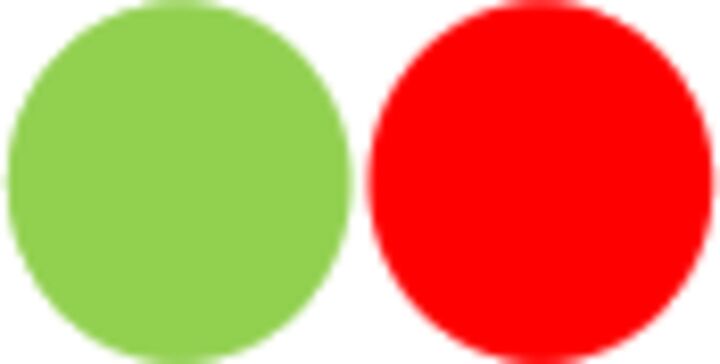					
	Torres 2018^67^	✓			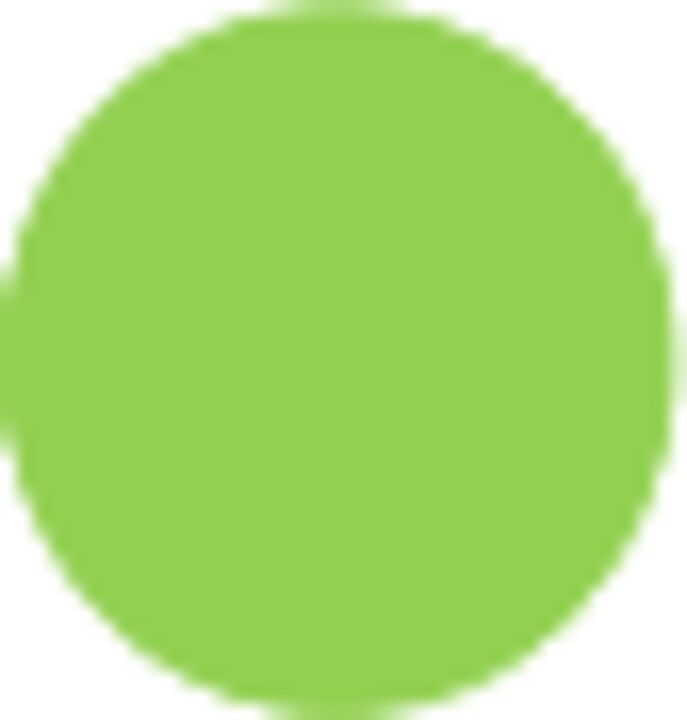			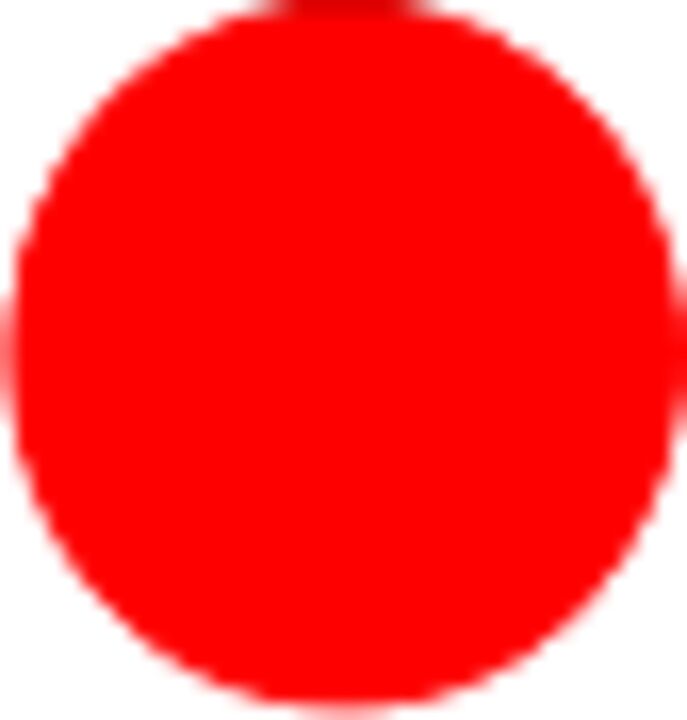	
Interventions targeting women attending services other than FP									
Systematic provision of contraceptive counselling	Gillespie 2009^74^			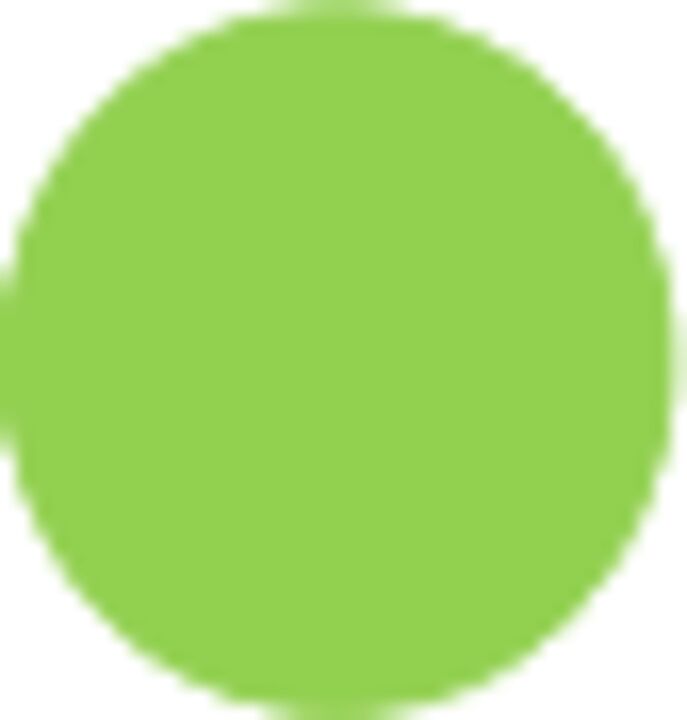					
	Grubb 2018^73^		✓	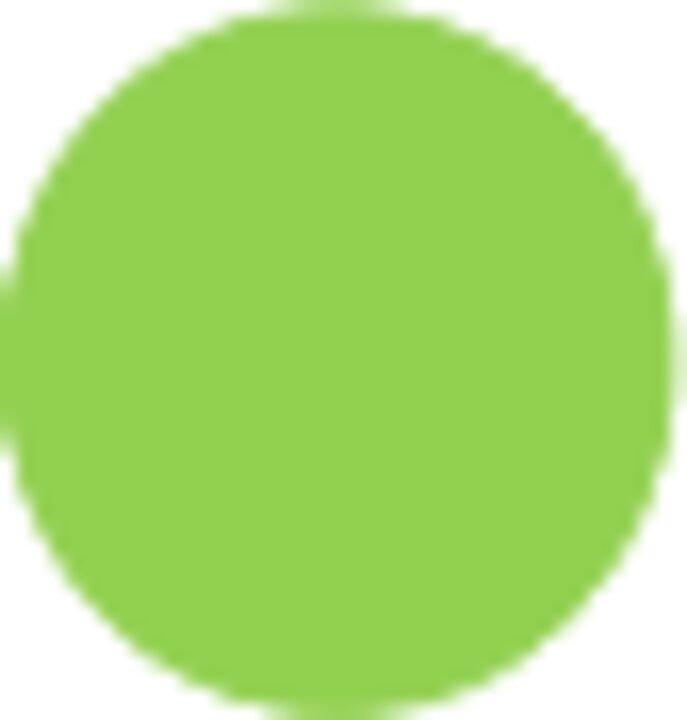					
	Lee 2015^75^			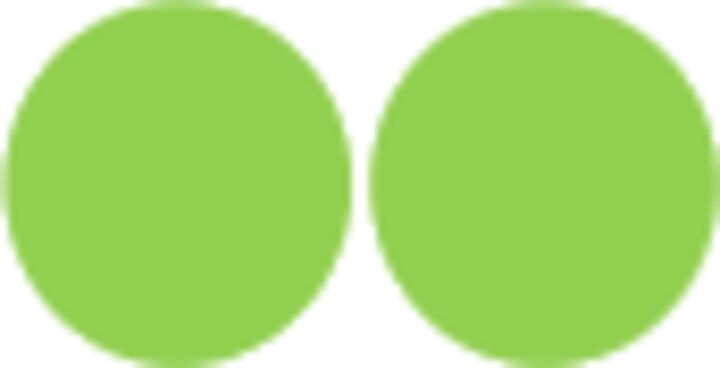 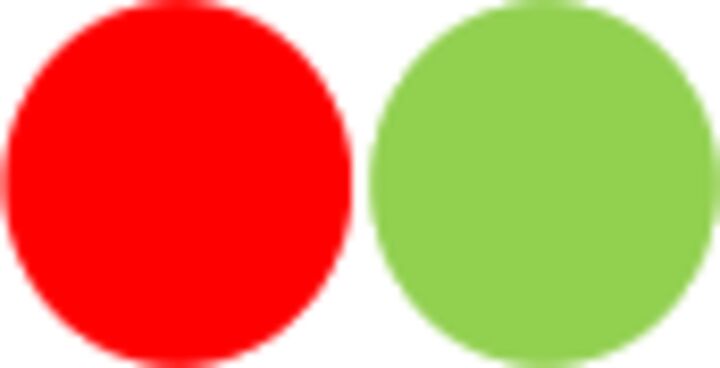	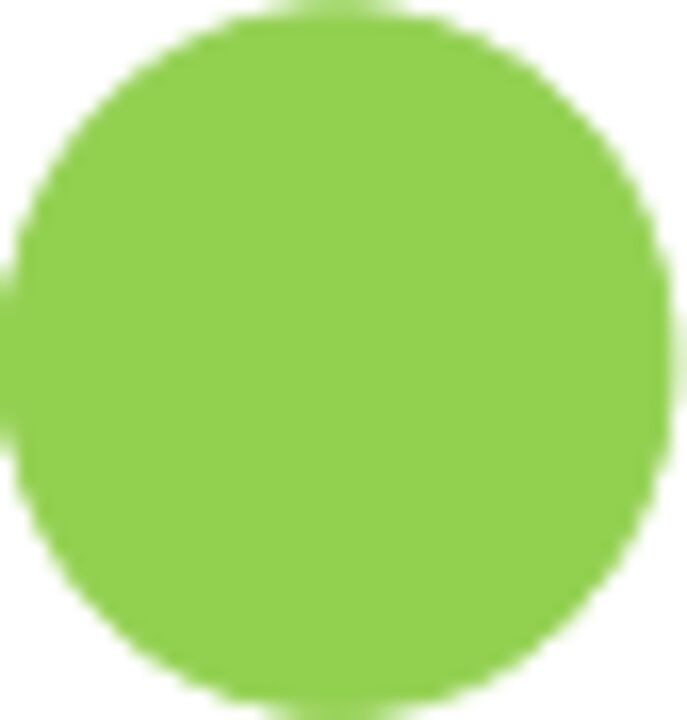		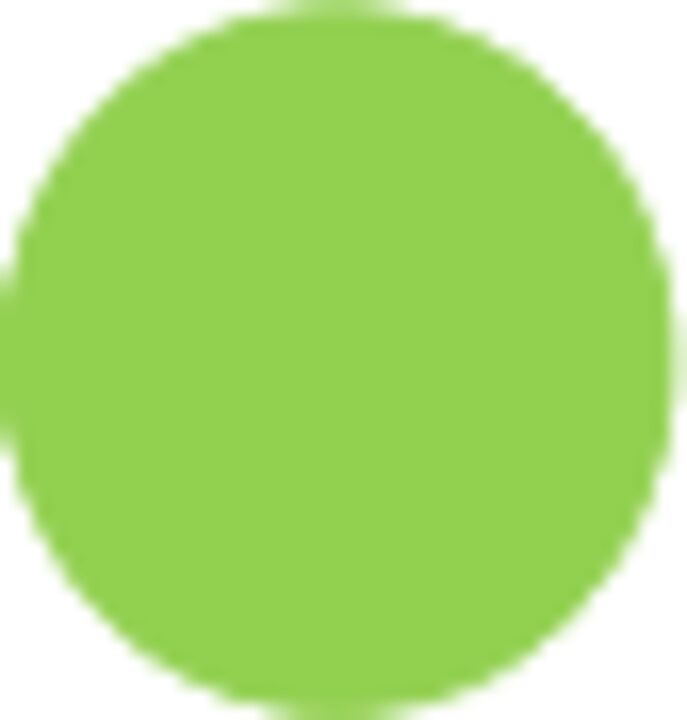		
	Yassin 2005^76^			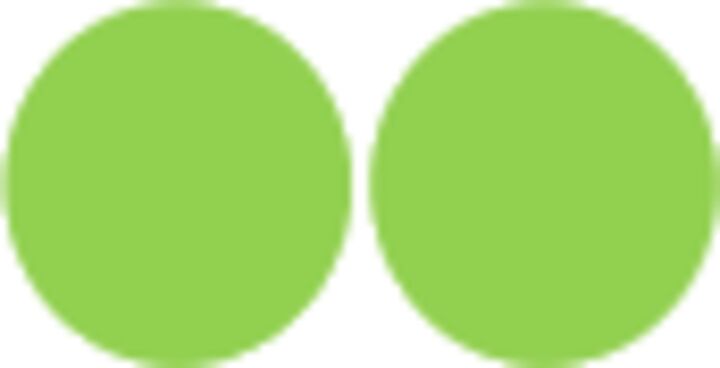					
Community-based interventions									
Peer counselling	Ferguson 1998^80^	✓	✓	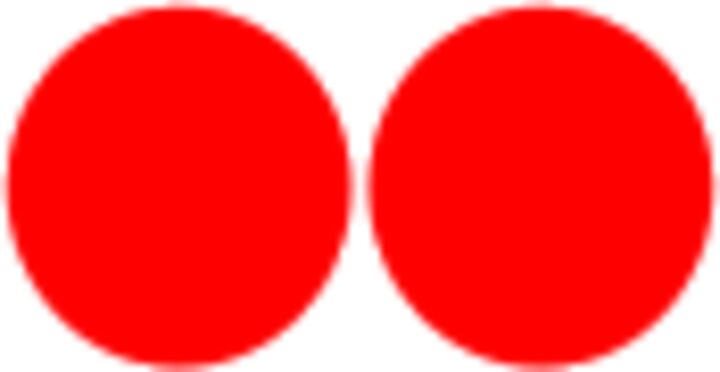					
Couples counselling	El-Khoury 2016^78^	✓		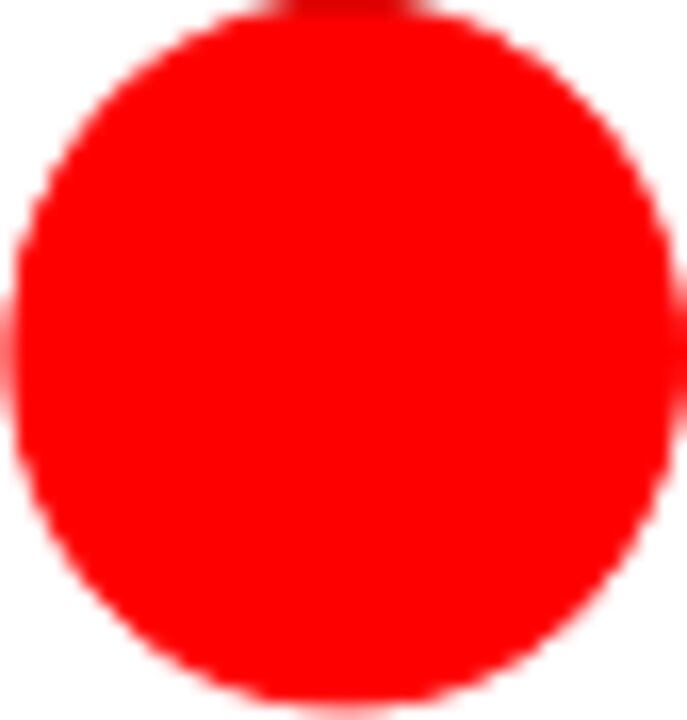					
	Lemani 2017^79^	✓	✓	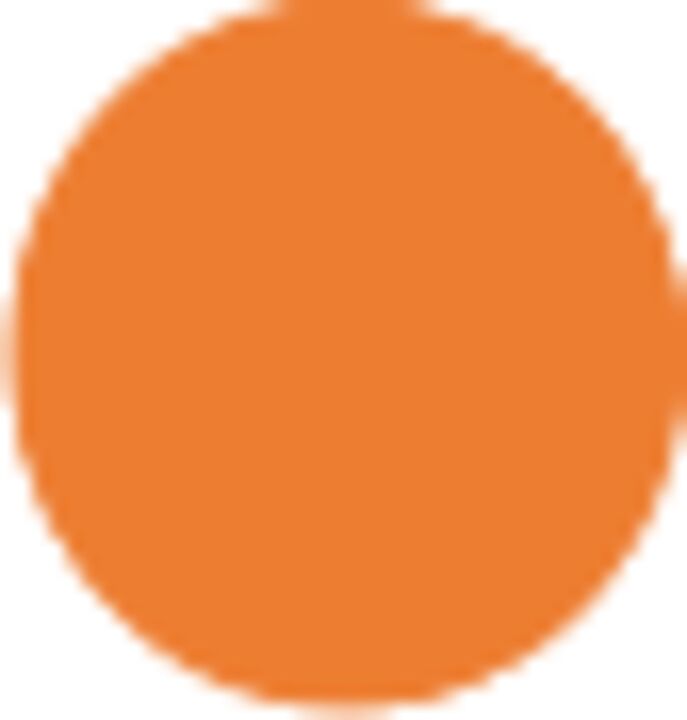	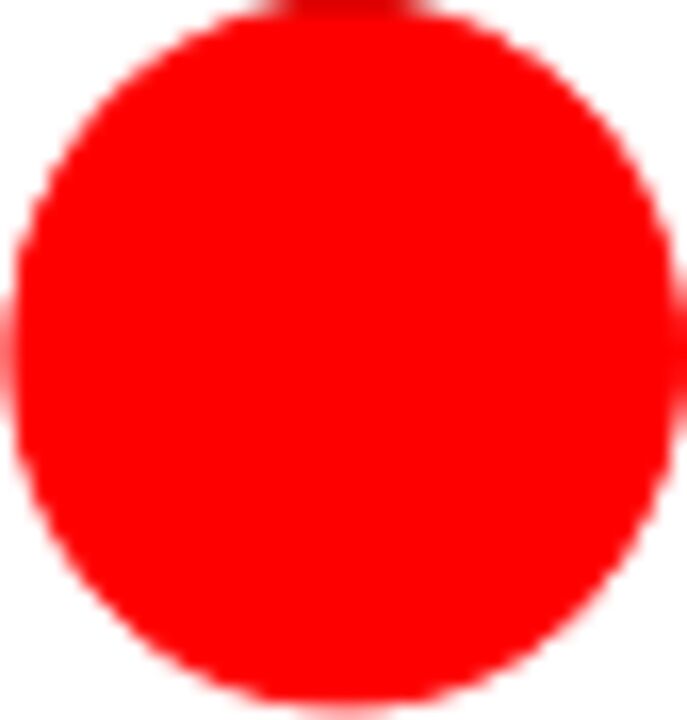				
	Terefe 1993^77^	✓		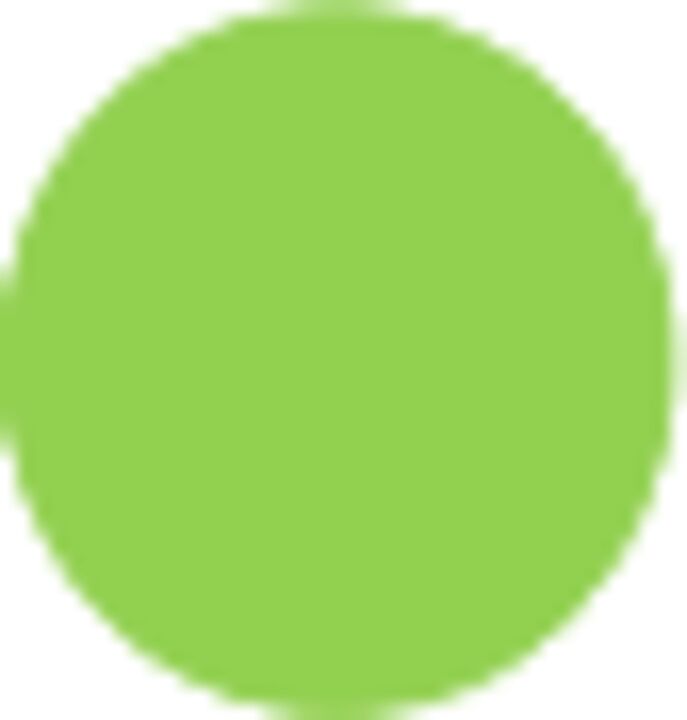	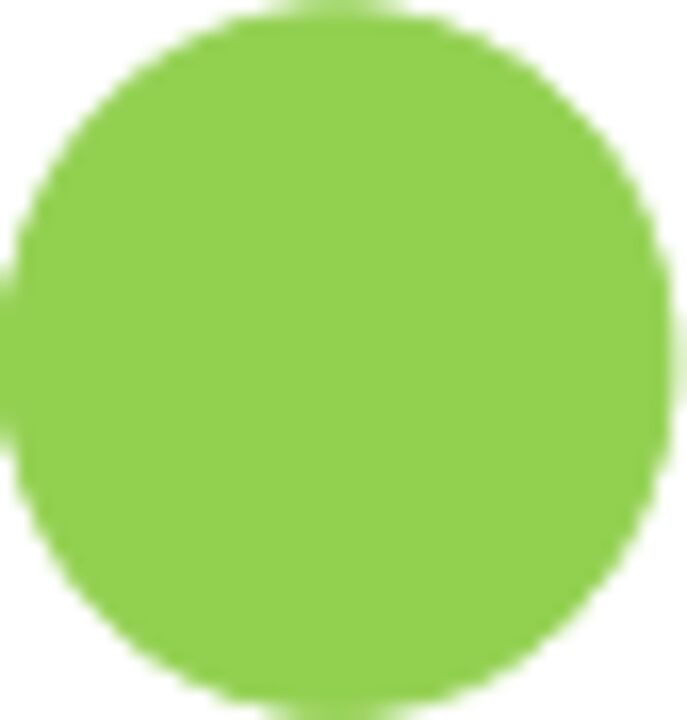				

Each dot represents one outcome measure unless otherwise specified. 
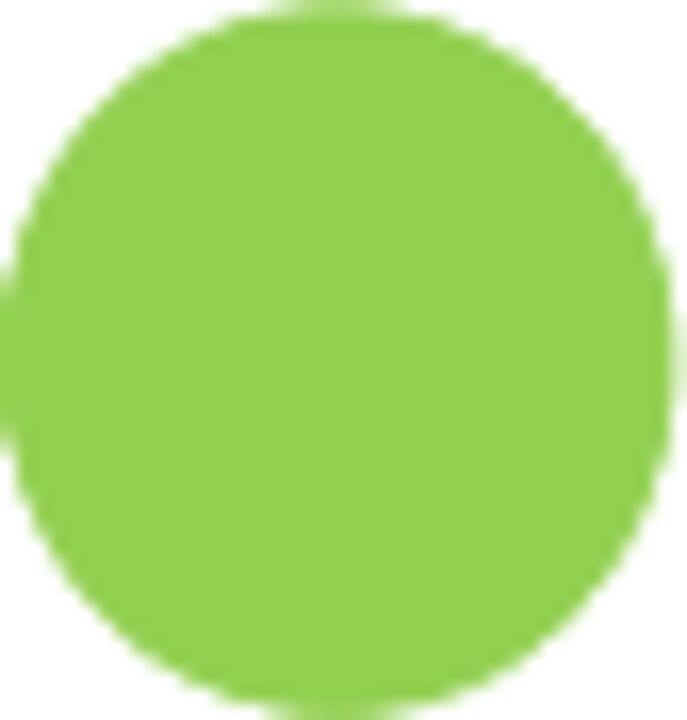
 Positive effect (p<0.05). 
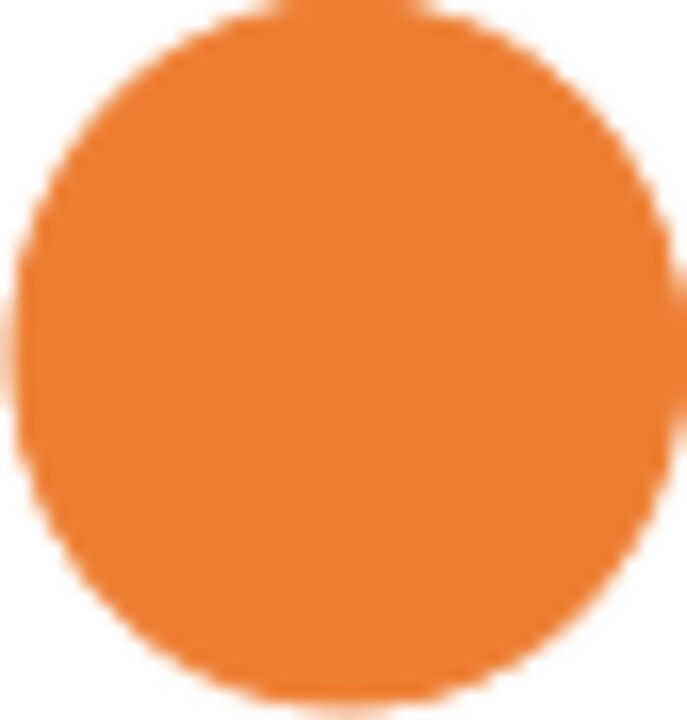
 Weak evidence of positive effect (0.05<p<0.1). 
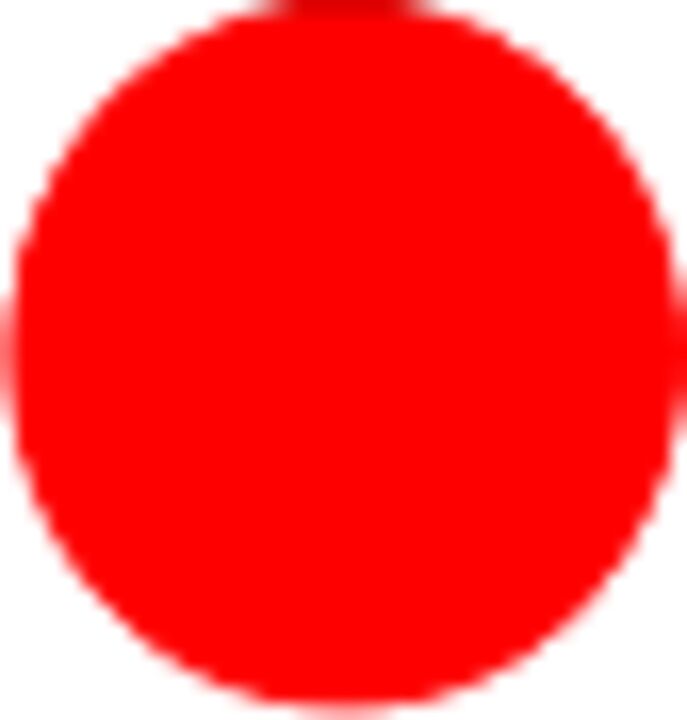
 No evidence of effect or negative effect.

A+YW, adolescents and young women; FP, family planning; RCT, randomised controlled trial.

#### Interventions targeting women choosing a contraceptive method

Eleven studies targeted women choosing a method, including seven studies of digital decision-making aids in the USA. Two targeted young women, with mixed results on immediate contraceptive uptake and no effect on longer-term use.^28 29^ Among women of all ages, computer-based algorithms with tailored printout were associated with increased choice of effective method and use at follow-up.^21 23^ Other studies found no or negative effect on contraceptive behaviour.^30 31^ Digital tools were associated with lower counselling satisfaction compared with health educators,^31^ but higher than pamphlets only.^32^

Studies of paper-based decision aids for face-to-face counselling found increased method selection and satisfaction with services after training on WHO Decision-Making Tool in Iran,^33^ and increased LARC uptake with shared decision-making brochures in the USA.^34^ However, Balanced Counselling Strategy training – using method-specific counselling cards^35^ – had no effect on contraceptive use, continuation or clinic satisfaction in Peru^36^ or Egypt.^25^

#### Interventions targeting women requesting or initiating a chosen method

Nine studies examined counselling strategies to improve continuation among women requesting a method. Among young women using oral contraceptives, daily text messages improved continuation,^37^ but evidence was inconclusive for health belief model-based counselling.^38^ Among women of all ages, continuation increased with detailed counselling on side effects in China^39^ and Mexico,^39^ but not Brazil.^40^ Counselling addressing IUD-related beliefs halved discontinuation in rural India,^41^ and tubal ligation scoring was associated with fewer requests for reversal in Turkey.^42^ Introducing the WHO Decision-Making Tool did not affect continuation among pill and injectable initiators in Nicaragua.^26^ Women with husband counselling had higher continuation in Bangladesh.^43^

The two studies reporting satisfaction outcomes found no or negative effects.^38 43^

#### Interventions targeting all FP service users to improve quality of care

Nine studies examined interventions to improve the quality of FP services among all clients. In the USA, training on LARC clinical skills and client-centred counselling produced an increase in LARC uptake among adolescents aged 18–19 years, but not among 18–25-year-olds as a whole.^44^ Broad-ranging clinical, counselling and logistical training in Senegal^45^ and the Philippines^24^ found no difference in short- or long-term contraceptive use. Training in the Balanced Counselling Strategy or GATHER approach was associated with increased contraceptive use in some settings but not others.^27 46–48^ No intervention had a positive effect on client satisfaction, except for one in China.^49^

Patient coaching prior to initial consultation did not affect continuation in Indonesia.^50^

#### Interventions targeting women undergoing abortion

Eleven studies focused on women undergoing abortion, including three RCTs among young women. In the USA, additional motivational interviewing increased LARC initiation and satisfaction with counselling, with no effect on overall method satisfaction or use.^51^ In China, individual counselling, male partner involvement, and free contraception increased modern contraceptive use at 6 months postabortion (adjusted OR 2.03, 95% CI 1.04 to 3.98) compared with group education only.^52^ However, a video on LARC information did not have any effect on contraceptive or satisfaction outcomes in the USA.^53^

Studies of women of all ages found additional physician counselling and expanded contraceptive provision in the UK,^54^ automated messages and access to telephone counselling in Cambodia,^55^ and personalised individual counselling in Brazil^20 22^ did not increase contraceptive use after the first few months postabortion. In the UK, women who opted for advance telephone counselling rather than face-to-face counselling during abortion consultation had 60% higher adjusted odds of receiving LARC or sterilisation.^56^ In Russia, provider training was followed by higher contraceptive use at 12 months; satisfaction results were mixed.^57^ Additional interview with a FP nurse in Iceland,^58^ husband counselling in Egypt,^59^ and WHO Decision-Making Tool in the USA^60^ had no effect on contraceptive behaviour.

#### Interventions targeting postpartum women

Thirteen studies examined interventions for postpartum initiation. Two studies among adolescents and young women found higher LARC uptake with additional pre-discharge counselling in Thailand^61^ and intensive motivational interviewing in a non-randomised study in the USA.^62^

Among women of all ages, additional antenatal or postpartum counselling was associated with increased postpartum use in two RCTs and one non-randomised study in Nepal,^63^ Nigeria^64^ and Turkey,^65^ but tailored antenatal counselling did not increase use in the UK, China or South Africa.^66^ Structured counselling increased LARC uptake after a preterm birth in the USA,^67^ and counselling on lactational amenorrhea increased modern method use by 10% 1 year postpartum in Brazil.^68^ Physician counselling was associated with higher satisfaction in a RCT in the USA,^69^ but did not increase postpartum use in Turkey,^70^ compared with leaflet/video counselling. Refresher provider trainings in Bangladesh^71^ and intensive counselling in Kenya^72^ were not associated with postpartum IUD uptake, continuation, or satisfaction.

#### Systematic contraceptive counselling for women attending services other than FP

Four pre-post studies found evidence of increases in contraceptive use after interventions systematising the provision of contraceptive counselling to all women attending non-FP outpatient services, including adolescents in juvenile detention in the USA,^73^ voluntary counselling and testing (VCT) clients in Ethiopia,^74^ women seeking pregnancy testing at a walk-in clinic in the USA,^75^ and women attending an abortion clinic in the UK.^76^ However, these interventions also included expanded method provision.

#### Community-based interventions

In four community-based counselling interventions, couples counselling for non-users was associated with higher uptake and continuation in Ethiopia,^77^ but not in Jordan^78^ or Malawi.^79^ A study of peer counselling had a small sample and high non-response, limiting interpretation.^80^

### Advantages and disadvantages of different types of counselling interventions

**Figure 2:**
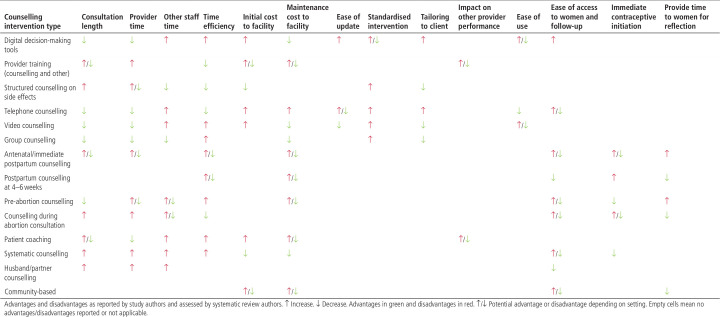
Summary of advantages and disadvantages of different counselling intervention types

Advantages and disadvantages of interventions included in this review are summarised in figure 2 and online supplementary table 3.

Only a few studies measured impact on consultation length, reporting increases^46 51^ or no change.^50^ Authors highlighted the implications of additional and longer consultations for patient volume and staffing, and associated costs of increases in staff, resources or contraceptive products. In contrast, interventions during waiting times prior to consultation (including digital tools) were described as potentially saving provider time.^31 50 81^

Development of custom digital tools was highlighted as expensive, but client population input was considered critical for success in several studies.^29 81^ Their potential for reaching digitally literate adolescents more widely, including in community contexts, was emphasised.^28^ Counselling satisfaction with digital tools alone was low, and these are best used in addition to, rather than instead of, face-to-face counselling.^31^ Telephone-based interventions have the advantage of providing access to many women at low cost, but they cannot reach women without phones,^55^ and can require many attempts to reach participants.^38^

Counselling up to the time of birth or abortion can reach women who may not access services at a later time and provide time for a fuller discussion of different methods,^56 64^ although some women may be reluctant to initiate contraception immediately, therefore effective follow-up mechanisms are needed.^55^ Conversely, routine postpartum counselling at 3–6 weeks may reach some women after they have resumed sexual activity.^64^ Involving male partners in counselling may target the main contraceptive decision-maker in some settings, although partner availability posed important logistical challenges.^52 59 78 79^

## Discussion

With the end of the FP2020 period in sight, this systematic review provides a timely synthesis of the effectiveness of different counselling interventions on contraceptive behaviour and client satisfaction. In our review, detailed counselling for women initiating contraception was associated with increased contraceptive continuation in non-randomised studies and one of two RCTs. Most studies of digital decision-making tools and provider training (including paper-based decision-making aids) did not find evidence of effect on contraceptive behaviour or satisfaction. Exceptions included training in the Population Council’s Balanced Counselling Strategy^35^ and WHO Decision-Making Tool,^82^ effective in some – but not other – settings. Interventions to systematically counsel women outside of FP services were associated with increased contraceptive use in contexts of expanded contraceptive provision; it is therefore not possible to attribute changes to counselling alone. Additional counselling sessions in pregnancy or postpartum appeared to increase postpartum contraceptive use, regardless of their timing. Non-randomised studies found evidence of effectiveness for several other postpartum and postabortion interventions. Male partner counselling was associated with increased contraceptive use in two of five studies.

Due to concerns with data quality and heterogeneity, caution is required in interpreting the evidence. Half (n=33) of 61 identified studies were RCTs, and although we did not assess study quality systematically, many non-randomised studies were prone to selection bias and few reported adjusted estimates. There was wide heterogeneity in intervention types, study designs, client populations, settings, and outcome measures across studies, and the evidence base is limited to a small number of studies for each of many interventions. Notable gaps include evidence on interventions targeting continuing users and women experiencing side effects, postpartum and postabortion decision-making tools, and community-based interventions other than couples counselling.

To our knowledge, this is the first systematic review to summarise the available evidence on comparative effectiveness of different counselling strategies across different client populations (including adolescents, postpartum and postabortion women). Our focus on comparing counselling strategies is critical to help identify successful interventions to improve contraceptive services. We examined outcomes directly affected by counselling (contraceptive behaviour and client satisfaction); however, preventing unmet need for contraception and unwanted pregnancies (influenced by multiple other factors) is the ultimate objective from a public health standpoint, and counselling process indicators such as client participation and knowledge are also important.

Our review has some limitations. Keywords for counselling interventions are not well defined; we maximised identification of relevant studies through a wide range of search synonyms and extensive manual searches. Advantages and disadvantages of different intervention types were assessed based on information reported by authors and a subjective assessment by reviewers, to facilitate assessment of intervention suitability to individual contexts.

There are also limitations to the evidence base. First, study quality was variable, particularly among non-randomised studies (almost half of the included studies). We did not assess risk of bias systematically, however, there was no or weak evidence for most intervention types in non-randomised studies. Selection bias is likely to overestimate the strength of association, with women opting into the intervention being more likely to use contraception, and a lack of effect is unlikely to hide a 'true' population effect. Non-randomised studies finding evidence of intervention effect – including structured counselling on side effects for women initiating a method – should be assessed in RCTs before recommendations can be made. We were unable to build forest plots because most studies reported percentages rather than effect estimates with confidence intervals, and we acknowledge the limitations of conclusions based on dichotomised p values.^83^

Second, substantial heterogeneity in study settings, interventions and outcomes limits the comparability of studies. Similar interventions were effective in some – but not all – settings, highlighting the importance of context as well as implementation intensity and fidelity. Effectiveness on contraceptive behaviour may be limited by contraceptive provision at the time of counselling (restricted in some abortion and postpartum clinics), cost of methods (particularly LARC as mentioned in the USA^30 34 44^ and Thailand^61^), availability of providers and resources, and quality of care. Findings are therefore unlikely to be generalisable to all settings, with a particularly limited evidence base outside of high-income countries (26 of 61 studies were conducted in the USA or UK).

Third, many included studies did not clearly state whether the intervention targeted women initiating, switching and/or continuing contraception, and women switching methods were often grouped with initiators. Furthermore, multiple studies referred to 'continuation' among all clients (not all of whom had initiated a method at the time of intervention), and most studies reported contraceptive use at follow-up among all women (including those no longer in need of contraception).

Several recommendations for programmes emerge from our findings. Where possible, repeated counselling throughout pregnancy and postpartum can contribute to maximising access to information and contraceptive uptake. Interventions seeking to improve contraceptive counselling need to be tailored to the patient flow, record flow and contraceptive methods available in each setting, and embedded within broader quality of care improvements (including clinical training, where necessary). Facilities implementing counselling interventions should monitor the impact on consultation duration, patient volume and method mix to ensure necessary staffing and resources.

Despite inconclusive evidence relating to contraceptive continuation, women initiating hormonal methods should receive detailed counselling on side effects and the possibility of changing methods if desired. Counselling on LARC should ensure respect for women’s informed choice.^84 85^ Contraceptive provision at abortion services is recommended as best practice in developing regions:^86 87^ where not possible, referrals should be optimised to ensure contraceptive needs are met. Male partner counselling should ensure strict consent procedures.^59^

There is a need for increased conceptual clarity in the literature (see recommendations in box 1). Appropriate denominators for contraceptive uptake (women not using a method at baseline) and continuation (women with a need for contraception at follow-up) should be used. Moreover, fertility intentions are fluid,^88–90^ and research is needed to identify reasonable length of follow-up for measuring counselling effect on contraceptive behaviour, including after abortion and childbirth.

Box 1 **Recommendations for reporting of studies of contraceptive counselling interventions**

**Intervention and participant characteristics**

- Clarify whether the intervention targets women initiating or switching contraception, continuing users, or a combination of these, and report results stratified according to these categories
- Specify whether women already using contraception and/or satisfied with their method are eligible for inclusion, and if so report the acceptance rate and percentage of participants in these groups

**Outcome measures**

- Report contraceptive use and continuation among women with a need for contraception (rather than all women) at each follow-up time point
- Report contraceptive behaviour outcomes within reasonable time frames during which fertility preferences may remain constant, including postpartum and postabortion (additional research will be needed to identify these time frames; shorter time frames may be more appropriate)
- Report switching outcomes for interventions targeting women undergoing abortion
- Where possible, report pregnancy outcomes (unwanted pregnancy and abortion) in addition to contraceptive behaviour outcomes
- Report measures of experience of contraceptive care; better measures of client satisfaction will need to be developed (including client-centredness and perceived quality of care)^91^

Future research should identify and evaluate interventions supporting contraceptive continuation, including identifying users with lower satisfaction over time and supporting method switching where required. Alternative interventions to counsel women before or after abortion, and mechanisms for referral and follow-up for those not wanting to initiate contraception at the time of abortion, are also needed. Lastly, improved measurement of satisfaction with counselling are needed; recent efforts to develop measures of perceived quality or client-centredness of care^91^ may represent promising avenues.

## Conclusions

Contraceptive counselling has a key potential to improve effective use of contraception and reduce unmet need. This review indicates that additional sessions during pregnancy or postpartum may increase uptake and detailed counselling on side effects for women initiating a method may be effective at improving continuation. However, there was at best limited evidence for effectiveness of other contraceptive counselling interventions, and the evidence should be interpreted with caution given low-quality evidence and substantial heterogeneity. Improved reporting of studies of contraceptive counselling and novel effective interventions are needed.

### Additional educational resources

- Brief educational strategies for improving contraception use in young people (Cochrane review)
- Education for contraceptive use by women after childbirth (Cochrane review)
- Effectiveness of contraceptive counselling of women following an abortion (systematic review and meta-analysis)
- Mobile phone-based interventions for improving contraception use (Cochrane review)
