# Supplementary material for: A systematic review of the effectiveness of counselling strategies for modern contraceptive methods: what works and what doesn’t?
Source: BMJ Sex Reprod Health. 2019 Dec 11;46(4):254–69. doi: 10.1136/bmjsrh-2019-200377 (PMC7569400; doi:10.1136/bmjsrh-2019-200377)
Supplement: Supplementary data [file bmjsrh-2019-200377supp001.pdf]

A systematic review of the effectiveness of counselling strategies for modern contraceptive methods: what works and what doesn't?

Systematic review protocol

Francesca Cavallaro, Lenka Beňová, Onikepe Owolabi, Moazzam Ali

BACKGROUND

Description of the condition

Ensuring access to contraception is a fundamental part of upholding human rights as well as contributing to improved health outcomes, with direct benefits for individuals, families, communities and wider society. The right of couples and individuals to decide freely and responsibly if, when and how many children to have requires that contraceptive services be available, accessible, acceptable and of good quality, and provided on the basis of non-discrimination. This has been recognised in Sustainable Development Goal 3.7, which aims to ensure universal access to sexual and reproductive healthcare services including family planning.[1]

Despite increases in contraceptive use in the last several decades, the unmet need for family planning – defined as women who do not want to become pregnant but are not currently using modern contraception – remains high in many low- and middle-income countries (LMIC). In 2017, about half (885 million) of 1.6 billion women of reproductive age (15–49) in developing regions wanted to avoid pregnancy and about three-quarters (671 million) of them were using modern contraceptives. However the unmet need for contraception remains high, fuelled by both a growing population, and weak family planning services. According to the latest 2017 estimates, 214 million women of reproductive age in developing regions who want to avoid pregnancy are not using a modern contraceptive method. These women are considered to have an unmet need for modern contraception.[2] Women in need or their male partners may not use contraception for a number of reasons, including concerns for health and side effects of methods, lack of geographical or financial access to services, and lack of decision-making power for contraceptive use.[3-6]

Meeting the unmet need for contraception in LMICs would avert an estimated 67 million unintended pregnancies per year, as well as 36 million induced abortions and 76,000 maternal deaths each year.[2]

Description of the intervention

Contraceptive counselling is the provision of information and assistance in choosing a contraceptive method that meets the needs and preferences of clients. Counselling can be provided by healthcare providers, pharmacists or trained lay persons (such as peer counsellors), and its content may differ according to visit order or client need: for example, first visits and those where women want to switch methods may entail more comprehensive counselling than re-supply visits. Effective counselling can help women choose an appropriate contraceptive method, manage any side effects, and continue using their preferred method or switch to an alternative method. It should also lead to high client satisfaction.

Cavallaro FL, *et al. BMJ Sex Reprod Health* 2020; 46:254–269. doi: 10.1136/bmjsrh-2019-200377

Counselling content, format and interactions between client and provider can be amenable to interventions. The majority of interventions have targeted women, although some have targeted couples or men. A wide range of contraceptive counselling strategies have been tried, including structured (method-specific) counselling, comprehensive counselling (using extensive clinical algorithms), group counselling, and patient-centred approaches. A multitude of tools have been used such as visual Decision-Making Tools and recent computer-based counselling aids. The evidence on the effectiveness of these different approaches has not been synthesised.

Key components of quality contraceptive counselling have been proposed. The Bruce framework, proposed in 1990, identifies six dimensions for quality family planning services, including three which relate to contraceptive counselling: choice of methods, information given to clients, and interpersonal relations.[7] It is important that clients are given a choice of methods, sufficient information to make an informed choice, and are treated with respect by providers. Holt and colleagues define quality contraceptive counselling as consisting in three dimensions: needs assessment, decision-making support, and method choice and follow-up.[8] They argue that a patient-centred approach should be used by assessing needs and tailoring communication based on these responses. Similarly, Dehlendorf *et al.* suggest that best practices for contraceptive counselling include building a trusting relationship with the client and using a shared decision-making approach, based on eliciting and responding to patient preferences.[9] They further suggest that counselling on side effects and using specific strategies to promote adherence can help improve contraceptive use. Attention has been called to the specific needs of and barriers faced by adolescents, including a need for dual protection (against pregnancy and STIs) and respect for adolescents' autonomy.[10]

Despite these diverse frameworks on quality of contraceptive services, no clear consensus exists on how to deliver contraceptive counselling in such a way as to meet contraceptive needs and patient satisfaction. The WHO 2016 *Selected practice recommendations for contraceptive use* highlights counselling content that should be provided to women, primarily concerning method-specific side effects and prevention and treatment of STIs.[11] The WHO 2018 *Global Handbook for Family Planning Providers* includes recommendations on content as well as interpersonal qualities (such as showing every client respect, encouraging clients to express concerns, and assuring them of confidentiality).[12] Guidance on the best format for delivering the information during counselling is limited.

The limited evidence available shows that the quality of contraceptive counselling is poor in low-resource settings:[13] in Senegal, only 18% of providers counselled their clients on all three examined items (how to use their methods, possible side effects, and when to return to the clinic).[14] High-quality contraceptive counselling has the potential to ensure that women and couples choose the method best suited to their needs and preferences, are aware of potential side effects and return to the provider as needed, thereby addressing concerns related to health and side effects and ensuring continuation among women who want to use contraception

Why it is important to do this review

Several reviews have examined counselling strategies to improve contraceptive use. A meta-analysis of three RCTs found no evidence that expert individualised contraceptive counselling was associated with contraceptive continuation after abortion.[15] Another systematic review found mixed evidence that brief educational strategies in clinic settings for adolescents and young people reduced pregnancy rates and increased contraceptive use.[16] Postpartum interventions for contraceptive use showed only limited evidence of a reduction in subsequent repeat pregnancies, compared to routine care or no intervention.[17] These

Cavallaro FL, *et al.* *BMJ Sex Reprod Health* 2020; 46:254–269. doi: 10.1136/bmjsrh-2019-200377

reviews have focused on subgroups of women, with often a limited description of the contraceptive counselling received by the control group. Another Cochrane review also showed limited evidence that mobile phone interventions increased uptake or adherence to contraception outside of clinic settings.[18]

In this review we examine the effectiveness of counselling strategies to increase contraceptive uptake and continuation among women of reproductive age, including post-partum and post-abortion women. The intervention should involve provision of contraceptive counselling to women (through the provision of information and assistance in decision-making), and the control group should include an alternative counselling strategy. Contraceptive counselling may occur within health facilities, or outside of health facilities (such as through outreach strategies or remotely using digital communication tools).

WHO is in the process of developing guidelines for the provision of quality contraceptive services, and this systematic review intends to provide relevant information on what works and what doesn't work in contraceptive counselling to support this process.

OBJECTIVES

The aim of this systematic review is to synthesise the evidence on the comparative effectiveness of different techniques for contraceptive counselling, and examine their advantages and disadvantages.

METHODS

Criteria for considering studies for this review

Types of studies

In this review, we will consider randomised controlled trials (RCTs) and non-randomised studies. RCTs can include both individual and cluster-randomised trials, such as for health facilities. Non-randomised studies will need to be prospective intervention studies and compare at least two contraceptive counselling techniques. There may be limited evidence from RCTs on techniques for contraceptive counselling, due to funding limitations and clinical logistics, and therefore it is important to consider the evidence base from non-randomised studies.

Only studies reporting quantitative findings will be considered.[a]

Types of participants

We will include studies with participants who are women of reproductive age or couples, who may be seeking to initiate contraceptive use, switching contraceptive methods or continuing to use the same method. Participants may include women who are post-abortion or post-partum (including breastfeeding women), but will exclude women with specific medical conditions (such as breast cancer, or heart disease). We will consider evidence from all countries.

[a] Relevant qualitative studies on contraception and counselling identified during the search will be compiled into a bibliographical appendix for reference

Types of interventions

Contraceptive counselling interventions which include provision of information and decision-making support for contraceptive methods will be included. We will consider counselling techniques for contraception in health facilities, in the community or remotely via digital communication, delivered by health providers, lay persons or electronically. Counselling methods may include direct oral communication or the use of digital technologies (such as computer-assisted algorithms), or a combination of both. The comparison intervention must include a type of contraceptive counselling, including usual care where it is clear that usual care includes contraceptive counselling (rather than no intervention).

Contraceptive counselling strategies will be considered for modern family planning methods, as defined by Festin et al.[19] However, counselling interventions focusing solely on male condoms will be excluded.

Example of types of interventions (some combine multiple facets)

- Content of counselling
  - Range of methods (comprehensive vs. reduced list of methods presented)
  - How to use method
  - Side effects and in what cases to return to provider
- Format of counselling
  - Group
  - Individual
  - Structured counselling (provider is given a list of information to discuss, usually for a single method)
  - Intensive counselling (e.g. follow-up sessions with same provider)
  - Individualised counselling (tailored to client)
  - Interactive counselling (related to patient-centred communication)
  - Balanced counselling
  - Decision-making tools
  - Video counselling
- Client-provider interactions
  - Client-centred/interpersonal skills
  - Decision-making tool for clients
  - Male partner involvement
  - Counsellor-guided motivational interviewing

Types of outcome measure

Primary outcomes

Included studies must include at least one of the quantitative outcome measures listed below:

- Contraceptive use
  - Uptake of a modern contraceptive method (at the time of the intervention)
  - Switching to a different modern method (at the time of the intervention)
  - Continued use of modern contraceptive method (at least three months after the intervention began)
- Client satisfaction
  - With contraceptive method used
  - With contraceptive services received

Cavallaro FL, *et al. BMJ Sex Reprod Health* 2020; 46:254–269. doi: 10.1136/bmjsrh-2019-200377

Measures of contraceptive continuation will exclude condom use at last sex, and consider only condom use when included as part of a range of modern contraceptive methods. Studies may not ask clients for their satisfaction with the counselling specifically, we will consider studies reporting general satisfaction with contraceptive services received.

Secondary outcomes

We will also synthesise any reported qualitative data on advantages and disadvantages of different counselling techniques in the included studies.

Advantages and disadvantages reported by contraceptive providers and users will be reported.

Table 1. Summary of inclusion and exclusion criteria

	**Inclusion**	**Exclusion**
**Study design**	Randomised controlled trials, prospective non-randomised quantitative studies	Qualitative studies
**Participants**	Women or couples of reproductive age, including post-abortion, postpartum and breastfeeding women seeking to initiate or continue contraceptive use	• Women with medical conditions affecting eligibility for contraceptive methods (including cardiovascular disease, cancer and HIV – see WHO family planning handbook for providers p.388-399 [12])<br>• Men only
**Intervention**	• Contraceptive counselling (information provision and decision-making support) in a health facility, in the community or remotely<br>• Addressing the format, content or client-provider interaction of counselling	• Interventions providing information on contraceptive method without decision-making support<br>• Counselling for male condoms or barrier methods only
**Control intervention**	Contraceptive counselling	No contraceptive counselling
**Contraceptive methods**	Modern methods (oral contraceptive pill, implant, injectable, copper intra-uterine device, hormonal intra-uterine system, patch, diaphragm, male condom, female condom, spermicides, emergency contraception, lactational amenorrhea, standard days method, male and female sterilisation)	Traditional methods
**Outcomes measures**	• Uptake of modern method<br>• Use of modern method<br>• Continuation of modern method<br>• Switching to different modern method<br>• Client satisfaction with method<br>• Client satisfaction with services	Protected sex, condom use only
**Geographic location**	All countries	
**Language**	English	Other languages
**Publication type**	Peer-reviewed article, review, report	Conference abstract, letter
**Publication date**	1st January 1990-31st October 2018	Before 1990

Search methods for identification of studies

Electronic searches
We will search for eligible studies in Medline, Embase, Global Health, Popline, CINAHL and Cochrane Database of Systematic Reviews electronic databases, using keywords related to contraception, counselling and outcomes of interest. Searches will focus after the publication of the Bruce framework,[7] from 1st January 1990 to present (end October 2018). We will restrict to studies published in English. We will review the reference lists of included studies to identify any publications not identified by the search strategy. The proposed search strategies are shown in Appendix 1.

Searching other resources
We will perform a preliminary manual search of a key journal (*Contraception*) to identify key words to capture relevant studies.

Data collection and analysis

Selection of studies
All unique studies retrieved by the search strategy will be assessed for inclusion based on title and abstract by one author. For studies which appear eligible for the review, we will obtain and assess full-text articles. A second reviewer will assess 10-20% of references excluded during title and abstract screen, and any differences will be reconciled by discussion between co-authors.

Data extraction and management
The extracted data will be entered into an Excel spreadsheet. Information extracted will include:

- **General information**: first author, title, year of publication, country
- **Study characteristics**: study design, aim of study, participant recruitment, sampling, method of allocation, inclusion/exclusion criteria
- **Participants**: description, geographic location, sample size
- **Intervention**: type of intervention (initiation, re-supply, switching, all/unspecified), description, aim of intervention, providers delivering intervention, medium/format of intervention, content of intervention, duration, frequency, co-interventions
- **Comparison**: description of control intervention
- **Outcomes**: outcomes evaluated (among outcomes included within the review scope), length of follow-up, methods of assessing outcomes, completeness of outcome data, results for each outcome, type of analysis (intention to treat or treatment received analysis)

10-20% of included full-text articles will be extracted in duplicate by two reviewers and any differences will be reconciled by discussion.

Assessment of quality of evidence

We will not systematically grade the quality of evidence for each study.

Assessment of heterogeneity

Due to the variability in contraceptive counselling techniques and outcomes reported, we anticipate there will be limited scope to conduct a meta-analysis. We will report any differences across studies by measure of contraceptive use (uptake, continuation, switching) and participant populations (such as adolescents and young people, post-partum or post-abortion women). If appropriate, we will synthesise results by the type of intervention (such

as in-person or digital counselling), and whether the contraceptives were provided to participants at no or reduced cost as part of the intervention.

Data synthesis

We will present a narrative overview of the findings together with tables summarising the extracted data. Summary and descriptive statistics will be presented.

ACKNOWLEDGMENTS

We based this protocol on the prior work of Lopez and colleagues[16] and Smith and colleagues.[18]

REFERENCES

1. United Nations. Sustainable Development Goal 3. Secondary Sustainable Development Goal 3. https://sustainabledevelopment.un.org/sdg3.
2. Guttmacher Institute. Adding it up: Investing in Contraception and Maternal and Newborn Health, 2017. Secondary Adding it up: Investing in Contraception and Maternal and Newborn Health, 2017 2017. https://www.guttmacher.org/sites/default/files/factsheet/adding-it-up-contraception-mnh-2017.pdf.
3. Ali M, Cleland J, Shah IH. Causes and consequences of contraceptive discontinuation: evidence from 60 demographic and health surveys. Egypt: World Health Organisation, 2012.
4. Haider TL, Sharma M. Barriers to family planning and contraception uptake in sub-Saharan Africa: a systematic review. International quarterly of community health education 2012;**33**(4):403-13.
5. Campbell M, Sahin-Hodoglugil NN, Potts M. Barriers to fertility regulation: a review of the literature. Stud Fam Plann 2006;**37**(2):87-98.
6. UNFPA. The Global Programme to Enhance Reproductive Health Commodity Security. Annual Report 2013. Secondary The Global Programme to Enhance Reproductive Health Commodity Security. Annual Report 2013 2014. http://www.unfpa.org/sites/default/files/pub-pdf/GPRHCS%20Annual%20Report%202013_web.pdf.
7. Bruce J. Fundamental elements of the quality of care: a simple framework. Stud Fam Plann 1990;**21**(2):61-91.
8. Holt K, Dehlendorf C, Langer A. Defining quality in contraceptive counseling to improve measurement of individuals' experiences and enable service delivery improvement. Contraception 2017;**96**(3):133-37.
9. Dehlendorf C, Krajewski C, Borrero S. Contraceptive counseling: best practices to ensure quality communication and enable effective contraceptive use. Clin Obstet Gynecol 2014;**57**(4):659-73.
10. Raidoo S, Kaneshiro B. Contraception counseling for adolescents. Current Opinion in Obstetrics and Gynecology 2017;**29**(5):310-15.
11. World Health Organisation. Selected practice recommendations for contraceptive use. Secondary Selected practice recommendations for contraceptive use 2016. http://apps.who.int/iris/bitstream/10665/252267/1/9789241565400-eng.pdf.
12. World Health Organization and Johns Hopkins Bloomberg School of Public Health. Family planning: a global handbook for providers. Secondary Family planning: a global handbook for providers 2018. http://apps.who.int/iris/bitstream/handle/10665/260156/9780999203705-eng.pdf?sequence=1.
13. Assaf S, Wang W, Mallick L. Provider Counseling and Knowledge Transfer in Health Facilities of Haiti, Malawi, and Senegal. DHS Analytical Studies No. 60. Secondary Provider Counseling and Knowledge Transfer in Health Facilities of Haiti, Malawi, and Senegal. DHS Analytical Studies No. 60 2016. http://dhsprogram.com/pubs/pdf/AS60/AS60.pdf.
14. Assaf S, Wang W, Mallick L. Quality of Care in Family Planning Services at Health Facilities in Senegal. DHS Analyticall Studies No. 55. Secondary Quality of Care in Family Planning Services at Health Facilities in Senegal. DHS Analyticall Studies No. 55 2015. http://dhsprogram.com/pubs/pdf/AS55/AS55.pdf.
15. Ferreira AL, Lemos A, Figueiroa JN, et al. Effectiveness of contraceptive counselling of women following an abortion: a systematic review and meta-analysis. Eur J Contracept Reprod Health Care 2009;**14**(1):1-9.
16. Lopez LM, Grey TW, Tolley EE, et al. Brief educational strategies for improving contraception use in young people. Cochrane Database Syst Rev 2016;**3**:Cd012025.
17. Lopez LM, Grey TW, Hiller JE, et al. Education for contraceptive use by women after childbirth. Cochrane Database Syst Rev 2015(7):Cd001863.
18. Smith C, Gold J, Ngo TD, et al. Mobile phone-based interventions for improving contraception use. Cochrane Database Syst Rev 2015(6):Cd011159.
19. Festin MP, Kiarie J, Solo J, et al. Moving towards the goals of FP2020 - classifying contraceptives. Contraception 2016;**94**(4):289-94.

APPENDICES

Appendix 1. Search strategies (05/11/2018)

Medline

1.	(contracepti* or family planning or birth control or depo?-medroxyprogesterone or depo? Medroxyprogesterone or Depo-Provera or Sayana Press or IUD or IUDS or IUS or intra?uterine device* or intra?uterine system* or cervical cap* or vaginal diaphragm* or vaginal ring* or implanon or jadelle or norplant* or sterili?ation or vasectomy).ti,ab. or *CONTRACEPTION/ or *CONTRACEPTION BEHAVIOR/ or *LONG-ACTING REVERSIBLE CONTRACEPTION/
2.	(counselling or counseling or education* strateg* or education* program* or education* intervention* or decision* tool or decision* aid or selection tool or (quality ADJ4 family planning) or (quality ADJ4 contracepti*) or patient-provider interaction* or client-provider interaction* or client-provider communication or patient-provider communication).ti or *COUNSELING/
3.	(continu* or discontinu* or uptake or initiat* or switch* or satisf* or “use” or “using” or “used”).ti,ab
4.	1 AND 2 AND 3
5.	4 NOT HIV.ti
6.	limit 5 to English
7.	limit 6 to ed=19900101-20181031

Embase

1.	(contracepti* or family planning or birth control or depo?-medroxyprogesterone or depo? Medroxyprogesterone or Depo-Provera or Sayana Press or IUD or IUDS or IUS or intra?uterine device* or intra?uterine system* or cervical cap* or vaginal diaphragm* or vaginal ring* or implanon or jadelle or norplant* or sterili?ation or vasectomy).ti,ab. or *contraception/ or *oral contraception/ or *hormonal contraception/ or *long-acting reversible contraception/
2.	(counselling or counseling or education* strateg* or education* program* or education* intervention* or decision* tool or decision* aid or selection tool or (quality ADJ4 family planning) or (quality ADJ4 contracepti*) or patient-provider interaction* or client-provider interaction* or client-provider communication or patient-provider communication).ti or *counseling/
3.	(continu* or discontinu* or uptake or initiat* or switch* or satisf* or ‘use’ or ‘using’ or ‘used’).ti,ab
4.	1 AND 2 AND 3
5.	4 NOT HIV.ti
6.	limit 5 to English
7.	limit 6 to yr=1990-2018

Global Health

1.	(contracepti* or family planning or birth control or depo?-medroxyprogesterone or depo? Medroxyprogesterone or Depo-Provera or Sayana Press or IUD or IUDS or IUS or intra?uterine device* or intra?uterine system* or cervical cap* or vaginal diaphragm* or vaginal ring* or implanon or jadelle or norplant* or sterili?ation or vasectomy).ti,ab. or *contraception/
2.	(counselling or counseling or education* strateg* or education* program* or education* intervention* or decision* tool or decision* aid or selection tool or (quality ADJ4 family planning) or (quality ADJ4 contracepti*) or patient-provider interaction* or client-provider interaction* or client-provider communication or patient-provider communication).ti or *individual counseling/ or *group counselling/
3.	(continu* or discontinu* or uptake or initiat* or switch* or satisf* or ‘use’ or ‘using’ or ‘used’).ti,ab
4.	1 AND 2 AND 3
5.	4 NOT HIV.ti
6.	limit 5 to English
7.	limit 6 to yr=”1990 –current”

Popline

contracepti* OR "family planning" [All fields]

AND

counselling OR counseling OR "decision tool" OR "decision-making tool" OR "decision making tool" OR "decision support" [Title]

AND

continuation OR continued OR discontinuation OR discontinued OR uptake OR initiation OR initiated OR switch OR switching OR switched OR satisfaction OR satisfied OR use OR using OR used [All fields]

NOT
HIV [Title]

[Limits: language = English language, publication year 1990-2018]

CINAHL Plus

contracepti* OR "family planning"

AND

TI (counselling OR counseling OR "decision tool" OR "decision-making tool" OR "decision making tool" OR "decision support") NOT HIV

AND

continuation OR continued OR discontinuation OR discontinued OR uptake OR initiation OR initiated OR switch OR switching OR switched OR satisfaction OR satisfied OR use OR using OR used

[Limits: English language, publication date Jan 1990-October 2018]

Cochrane library of systematic reviews

contracepti* or "family planning"

AND

counselling or counseling or "educational strategies" or "educational interventions" or tool* or "family planning quality" or "quality of family planning" or "patient-provider interaction" or "patient provider interaction" or "client-provider interaction" or "client provider interaction" or "client-provider communication" or "client provider communication" or "patient-provider communication" or "patient provider communication"

AND

continu* or discontinu* or uptake or initiat* or switch* or satisf* or ‘use’ or ‘using’ or ‘used’

Cavallaro FL, *et al. BMJ Sex Reprod Health* 2020; 46:254–269. doi: 10.1136/bmjsrh-2019-200377
