# Supplementary material for: A systematic review of the effectiveness of counselling strategies for modern contraceptive methods: what works and what doesn’t?
Source: BMJ Sex Reprod Health. 2019 Dec 11;46(4):254–69. doi: 10.1136/bmjsrh-2019-200377 (PMC7569400; doi:10.1136/bmjsrh-2019-200377)
Supplement: Supplementary data [file bmjsrh-2019-200377supp002.pdf]

Supplementary table 1. Characteristics of included studies (n=61)

**WHO Regions** [a]	
Africa	8
Americas	29
South-East Asia	7
Europe	9
Eastern Mediterranean	4
Western Pacific	6
**Study design**	
Individual randomised controlled trial	29
Cluster randomised controlled trial	4
Quasi-experimental study	4
Non-randomised controlled study	9
Uncontrolled pre-post study	14
Non-randomised controlled pre-post study	1
**Intervention target population**	
Women choosing a method	11
Women requesting or initiating a specific method	9
All family planning service users	9
Women undergoing abortion	11
Postpartum women	13
Non-family planning service attenders	4
Women living in specific area (community-based)	4
Non-users	*3*
**Outcomes** [a]	
Contraceptive uptake	15
Contraceptive use	32
Contraceptive continuation	23
Contraceptive switching	1
Satisfaction with contraceptive method	8
Satisfaction with services	13

[a]One multi-country study took place in 3 continents and studies may report multiple outcomes, therefore the sum of studies is larger than the total number of studies included.

Cavallaro FL, *et al. BMJ Sex Reprod Health* 2020; 46:254–269. doi: 10.1136/bmjsrh-2019-200377

Supplementary table 2. Description of studies included in systematic review (n=61)

Ref. no.	Study (country)	Population (sample size)	Study design	Intervention vs. comparison	Outcome definition	Results
	**INTERVENTIONS FOR CONTRACEPTIVE DECISION-MAKING (N=11)**					
	Digital decision-aids for contraceptive decision-making					
32	Chewning 1999 (USA)	Young women (<=20yo) attending FP clinics (n=949)	Non-randomised controlled study	Interaction with computer-based decision aid + printout + routine consultation vs. routine consultation only	• % non-uptake of pill<br>• Mean number of months of pill use	**Use**<br>Among majority of clients intending to use the pill, weak evidence of lower non-uptake of pills in intervention than control group in Chicago (3.4% vs. 8.8%, p=0.05), but no difference in Madison (1.2% vs. 2.3%, p>0.05).<br>**Continuation**<br>No difference in months of use between intervention and control group in Chicago (8.19 vs. 8.64, p>0.05) or Madison (9.38 vs. 9.50, p>0.05)
28	Dehlendorf 2017 (USA)	Women attending FP clinic (n=83)	Uncontrolled pre-post study	Interaction with interactive, tablet-based tool + printout of method preferences + routine consultation vs. routine consultation only	% "completely satisfied" with method choice	**Satisfaction**<br>Weak evidence, higher in intervention than control group (29% vs. 12%, p=0.06)
21 & 23	Garbers 2012 & 2012 (USA)	Women attending FP clinic (n=2,448)	Randomised controlled trial (three arms)	Intervention + tailored (I+T): interaction with computer-based algorithm + tailored printout with best fit methods vs. Intervention + generic (I+G): interaction with algorithm + generic printout on available methods vs. control (C): generic printout	• % choosing effective method (WHO effectiveness tier 1 and tier 2)<br>• % continuing chosen method at 4 month follow-up	**Use**<br>Effective method uptake higher in intervention groups than control group (I+T: 73% vs. I+G: 76% vs. C: 61%; I+T vs. C OR = 1.56, 95% CI: 1.23-1.98; I+G vs. C OR = 1.74, 95% CI: 1.35-2.25 adjusted for recruitment site).<br>**Continuation**<br>Higher in I+T than control group (95% vs. 77%, p=0.002; OR= 5.48, 95% CI: 1.72-17.42 adjusted for recruitment site), but no difference between I+G and control group (82% vs. 77%, p=0.507; OR=1.31, 95% CI: 0.58-2.98 adjusted for recruitment site).
33	Hebert 2018 (USA)	Young women (15-29yo) attending FP clinics (n=221)	Randomised controlled trial	Interaction with tablet app with information on all contraceptive methods (including videos of user experiences) + routine consultation vs. routine consultation only	% LARC uptake at baseline and use at 3 month follow-up	**Uptake**<br>No difference at baseline between intervention and control groups (3.8% vs. 0.97%, p=0.37).<br>**Use**<br>No difference at 3 months (8.0% vs. 3.8%, p=0.34)
29	Kofinas 2014 (USA)	Women attending gynaecological or postpartum clinic (n=143)	Randomised controlled trial	Interaction with Facebook page on contraceptive information with video, diagram and games + standardised counselling vs. interaction with ACOG pamphlets on contraception + standardised counselling	Median counselling satisfaction score (max: 10)	**Satisfaction**<br>Higher in intervention than control group (10 vs. 6, p<0.001)

INTERVENTIONS FOR CONTRACEPTIVE DECISION-MAKING (N=11)

Ref. no.	Study (country)	Population (sample size)	Study design	Intervention vs. comparison	Outcome definition	Results
30	Koo 2017 (USA)	Women attending clinic for contraception or annual exam (n=330)	Uncontrolled pre-post study	Interaction with interactive "Smart choices" computer-based tool on method options + printout for provider on client characteristics and questions + routine consultation<br>vs. routine consultation only	• % women choosing any method and LARC during consultation<br>• Predicted probability of choosing LARC method during consultation	**Uptake**<br>Choosing any method similar in intervention and control groups (100% vs. 97%, no p-value reported).<br>Weak evidence of lower LARC choice in intervention than control group (11% vs. 19, p<0.1 across all methods).<br>Lower predicted probability of choosing LARC in intervention than control group (0.09 vs. 0.20 adjusted for socio-economic characteristics, p<0.01).
31	Sridhar 2015 (USA)	Women attending gynaecology clinic (n=120)	Randomised controlled trial	Interaction with "Plan A Birth Control" mobile application on methods in order of effectiveness + routine consultation<br>vs. standardised counselling from health educator on methods in order of effectiveness + routine consultation	• % selecting very effective method (IUD, IUS, implant)<br>• % "very satisfied" with counselling	**Uptake**<br>No difference between intervention and control group (52% vs. 57%, p=0.753 across all method categories).<br>**Satisfaction**<br>Lower in intervention than control group (57% vs. 92%, p<0.001 across all satisfaction categories)
	Paper decision-aids for contraceptive method choice during in-person counselling					
34	Farrokh-Eslamlou 2014 (Iran)	Women attending facilities for new contraceptive method (n=448)	Uncontrolled pre-post study	Counselling by provider trained in WHO decision-making tool<br>vs. no training	• % of clients selecting a method by end of consultation<br>• % "client satisfaction with services"<br>• % "client would recommend services to others"	**Use**<br>Method selection increased from 90% pre-intervention to 95% post-intervention (p=0.04).<br>**Satisfaction**<br>Client satisfaction with services increased from 72% pre-intervention to 99% post-intervention (p=0.03).<br>Recommendation to others increased from 56% to 98% (p<0.01).
35	George 2015 (USA)	Women attending FP clinic (n=6,818)	Uncontrolled pre-post study	Counselling with shared decision-aid brochures on implants and IUDs<br>vs. counselling with one-page all-method summary	% women with LARC insertion	**Uptake**<br>Increased from 1.7% pre-intervention to 2.9% post-intervention (p<0.005).
36	León 2003 (Peru)	Women attending FP clinic choosing a method at recruitment appointment (sample size unspecified)	Cluster randomised controlled trial	Counselling by provider trained in Balanced Counselling Strategy with method cards and client pamphlets<br>vs. no training	• % contraceptive use at 12 months post-intervention<br>• % life table continuation at 12 months<br>• % cumulative life table quality-related discontinuation (excluding discontinuation due to reduced need for contraception) at 12 months	**Use**<br>No difference between intervention and control group (75% vs. 71%, p<0.32).<br>**Continuation**<br>No difference in all-method continuation between intervention and control group (48% vs. 44%, p=0.4).<br>No difference in quality-related discontinuation between intervention and control group (32% vs. 39%, no p-value reported).

Cavallaro FL, *et al. BMJ Sex Reprod Health* 2020; 46:254–269. doi: 10.1136/bmjsrh-2019-200377

Ref. no.	Study (country)	Population (sample size)	Study design	Intervention vs. comparison	Outcome definition	Results
25	Nawar 2004 (Egypt)	Women attending FP clinic choosing a method at recruitment appointment (n=600)	Quasi-experimental study (matched clinics randomised to intervention or control)	Counselling by provider trained in Balanced Counselling Strategy with method cards and client pamphlets vs. no training	• % contraceptive use at 7 and 13 months post-intervention<br>• % cumulative all-method continuation at 7 months<br>• % pill- and injectable-specific continuation at 13 months<br>• % "will recommend clinic to friends" at 7 and 13 months	**Use**<br>Similar at 7 months between intervention and control groups (89.7% vs. 91.3%, no p-value reported), and at 13 months (83.4% vs. 86.1%, no p-value reported).<br>**Continuation**<br>No difference in continuation at 13 months between intervention and control groups for all methods (66% vs. 68.1% at 13 months, p>0.05), or for pills or IUDs specifically (p>0.05 for both).<br>**Satisfaction**<br>No difference between intervention and control group at 7 months (97.0% vs. 97.0%, p>0.05), or at 13 months (95.9% vs. 93.2%, p>0.05).
	**INTERVENTIONS TARGETING WOMEN REQUESTING OR INITIATING A CHOSEN METHOD (N=9)**					
	Structured counselling on side-effects					
38	Canto de Cetina 2001 (Mexico)	New **DMPA injectable** (intramuscular) users (n=350)	Randomised controlled trial	Structured counselling on side-effects of DMPA + side effect diary review vs. routine information on side-effects	% cumulative discontinuation rate at 12 months	**Continuation**<br>Lower discontinuation in intervention than control group (17.1% vs. 43.4%, p<0.05)
39	Lei 1996 (China)	**DMPA injectable** users (n=421)	Non-randomised controlled study	Structured counselling on side-effects of DMPA vs. routine counselling	% cumulative discontinuation rate at 12 months	**Continuation**<br>Lower discontinuation in intervention than control group (11% vs. 42%, p<0.001)
40	Modesto 2014 (Brazil)	Women requesting **implant, IUS or IUD** (n=297)	Randomised controlled trial	Counselling with leaflet on chosen LARC method focusing on bleeding changes + reminder phone calls + routine counselling vs. routine counselling only	% continuation at 12 months for IUD, IUS, and implant users	**Continuation**<br>No difference in IUD continuation between intervention and control group (65.9% vs. 70.0%, p>0.05).<br>No difference in IUS or implant continuation between intervention and control group (p>0.05 for both).
41	Patel 2003 (India)	**IUD** users (n=119)	Uncontrolled pre-post study	Expanded counselling on location of IUD and initial side-effects + community information programme vs. routine limited counselling on side-effects and concerns	• % continuation at 24 months post-insertion<br>• % cumulative method-related discontinuation at 24 months post-insertion	**Continuation**<br>Continuation increased from 21% pre-intervention to 38% post-intervention (no-p-value reported; adjusted RR=0.58, 95% CI: 0.38-0.90, p<0.05 adjusted for caste, age, education, reproductive intentions).<br>Method-related discontinuation lower post-intervention than pre-intervention (adjusted RR=0.45, 95% CI: 0.25-0.81, adjusted for age, education, caste, and reproductive intentions).
	Tubal ligation scoring					
42	Demir 2006 (Turkey)	Women requesting	Non-randomised	Tubal ligation scoring to identify women who may regret sterilisation	% women requesting reversal of tubal ligation	**Continuation**<br>Requests for sterilisation reversal lower in intervention than control group (0% vs. 3.6%, p=0.001)

Cavallaro FL, *et al. BMJ Sex Reprod Health* 2020; 46:254–269. doi: 10.1136/bmjsrh-2019-200377

INTERVENTIONS TARGETING WOMEN REQUESTING OR INITIATING A CHOSEN METHOD (N=9)

Ref. no.	Study (country)	Population (sample size)	Study design	Intervention vs. comparison	Outcome definition	Results
		**sterilisation** (n=785)	controlled study	vs. routine counselling without tubal ligation scoring		
	Paper-based decision-making tool					
26	Chin-Quee 2007 (Nicaragua)	New **pill or injectable** adopters (n=1,633)	Quasi-experimental study (recruited control clinics for intervention clinics)	Counselling by provider trained in using WHO decision-making tool<br>vs. no training	• % all-method continuation at 5-8 months follow-up<br>• % pill-specific and injectable-specific continuation at 5-8 month follow-up	**Continuation**<br>No difference in all-method continuation between intervention and control group (80% vs. 86%, p=0.12).<br>No difference in pill-specific continuation between intervention and control group (57% vs. 62%, p=0.49).<br>No difference in injectable-specific continuation between intervention and control group (73% vs. 79%, p=0.14)
	Provider training (with telephone counselling in one arm)					
56	Berenson 2012 (USA)	Young women (16-24yo) requesting **pill** (n=1,155)	Randomised controlled trial (3 arms)	Clinic group (C): counselling based on health belief model geared at lower health literacy assistance developing a cue for pill taking in daily routine<br>vs. Clinic plus telephone (C+P): C+ phone counselling on correct use and side-effects<br>vs. standard care (S): standardised counselling	• % pill continuation at 3, 6 and 12 months<br>• % satisfied with method at 3, 6 and 12 months	**Continuation**<br>Weak evidence of lower continuation at 3 months in C group (49.9%) than S and C+P groups (55.2% and 58.3%, respectively, p=0.06).<br>Weak evidence of lower continuation at 6 months in C group (31.9%) than S and C+P groups (37.4% and 39.3%, respectively, p=0.08).<br>No difference in pill continuation at 12 months between S, C and C+P groups (S: 19.8%, C: 18.0%, C+P: 19.8% p=0.77).<br>**Satisfaction**<br>Satisfaction with method was lower among women in C group at 3 months (49.0%) than in S group (54.3%) or C+P group (59.7%, p=0.03). This pattern also occurred at 6 months (C=37.8%, S=45.5%, C+P=50.2%, p=0.01).<br>No difference in satisfaction between the three arms at 12 months (C=31.8%, S=35.7%, C+P=34.4%, p=0.69)
	Educational text messages					
61	Castaño 2012 (USA)	Young women (<25yo) using the **pill** (n=962)	Randomised controlled trial	Daily educational text messages for 6 months (including risks, benefits, side-effects, use) + routine face-to-face counselling<br>vs. routine counselling only	• % women reporting to have taken pill in last 7 days at 6 month follow-up<br>• % women reporting pill use at last intercourse at 6 month follow-up	**Continuation**<br>Higher pill use in last 7 days in intervention than control group (64% vs. 54%, p=0.005; adjusted OR=1.44, 95% CI: 1.03-2.00, covariates not specified).<br>Higher pill use at last intercourse in intervention than control group (69% vs. 60%, p=0.03).
	Husband counselling					

Ref. no.	Study (country)	Population (sample size)	Study design	Intervention vs. comparison	Outcome definition	Results
79	Amatya 1994 (Bangladesh)	Married women adopting **implant** (n=617)	Non-randomised controlled study	Husband counselling (with or without wife)<br>vs. individual woman counselling	• % life-table discontinuation at 36 months<br>• % women would recommend implants to friend/relative<br>• % women intending to use second implant	**Continuation**<br>Lower discontinuation in intervention than control group (32 per 100 women vs. 42 per 100, p=0.07, HR=0.81, 95% CI: 0.56-1.16 adjusted for sociodemographic variables and clinics).<br>**Satisfaction**<br>No difference in implant recommendation (97% vs. 97%, no p-value reported).<br>Lower intention to use implant in future in intervention than control group (32% vs. 38%, no p-value reported).
	**INTERVENTIONS TO IMPROVE FAMILY PLANNING SERVICE QUALITY FOR ALL USERS (N=9)**					
	Provider training in contraceptive counselling and/or clinical and logistics skills					
43	Gibbs 2016 (USA)	Young women (18-25yo) attending FP or abortion clinic (n=1,500)	Cluster randomised controlled trial	Training of providers on LARC indications and eligibility, client-centred counselling and IUD placement<br>vs. no training	% initiating LARC method within 12 months	**Uptake**<br>Among adolescents (18-19), higher in intervention than control group (23.1 vs. 14.0 per 100 person-years, no p-value reported).<br>Among young women (20-25), similar in intervention and control group (20.6 vs. 19.1 per 100 person-years, no p-value reported).<br>Among all women, higher in intervention group (HR=1.45, 95% CI: 1.03-2.03, adjusted for age group and parity).
24	Jain 2012 (Philippines)	New users (n=1,728)	Quasi-experimental study (matched clinic randomised to intervention or control)	Training of providers in client-provider interaction + training of supervisors in facilitative supervision<br>vs. no training	• % modern contraceptive use at 3 year follow-up<br>• % satisfied with services at initial consultation	**Use**<br>No difference between intervention and control group (58.1% vs. 56.4%, p non-significant, no value reported).<br>**Satisfaction**<br>No difference between intervention and control group (99% vs. 98%, OR=1.5, p>0.05, adjusted for background characteristics)
44	Kim 1992 (Nigeria)	Women attending FP services (n=480)	Quasi-experimental study (nurses self-selected into receiving training)	Training of nurses on contraceptive counselling based on GATHER approach<br>vs. no training	• % provided with contraceptives at follow-up visits<br>• % recommending clinic to friends	**Continuation**<br>Among clients attending follow-up visit, higher contraceptive provision in intervention than control group (84% vs. 60%, p<0.001).<br>**Satisfaction**<br>No difference between intervention and control groups (96% vs. 95%, no p-value reported)

INTERVENTIONS TO IMPROVE FAMILY PLANNING SERVICE QUALITY FOR ALL USERS (N=9)

Ref. no.	Study (country)	Population (sample size)	Study design	Intervention vs. comparison	Outcome definition	Results
45	León 2003 (Guatemala)	Women attending clinic postpartum, post-abortion or for FP (n=3,140)	Uncontrolled pre-post study	Training of providers on Balanced Counselling Strategy with method cards and client pamphlets<br>vs. no training	• % contraceptive in first 6 months post-consultation<br>• % survival curve all-method continuation at 6 months	**Use**<br>No difference in use for any method in any of the first six months between intervention and control group (p non-significant, no value reported).<br>**Continuation**<br>No difference between intervention and control group (approximate 73% vs. 70%, no p-value reported).
46	Madden 2013 (USA)	Women attending clinic for FP, abortion, gynaecological or primary care consultation (n=7,637)	Non-randomised controlled study	Standardised, comprehensive counselling using GATHER approach + no-cost contraceptives<br>vs. non-standardised routine counselling + no-cost contraceptives	% initiating LARC at enrolment consultation	**Uptake**<br>No difference between intervention and control group (72% vs. 78%, p<0.001; RR=0.98, 95% CI: 0.94-1.02 adjusted for socioeconomic characteristics and STI history)
47	Sanogo 2003 (Senegal)	Women attending facilities for FP (n=1,320)	Non-randomised controlled study	Provider training on counselling, contraceptives, supervision, logistics + additional counselling by contraceptive counsellor<br>vs. no training + single provider counselling	• % contraceptive use at 16 month follow-up<br>• % satisfied with visit	**Use**<br>No difference between intervention and control group ((59% vs. 54%, p-value not significant, no value reported; OR=1.3, p<0.10 adjusted for reproductive intention, age, parity, age of youngest child, education, employment, ethnicity, religion, polygamy, relationship status).<br>**Satisfaction**<br>Lower in intervention than control group (94% vs. 100%, p<0.01)
48	Sapkota 2017 (Nepal)	Married women attending FP or abortion clinic (n=7,806)	Uncontrolled pre-post study	Training of providers on personalised Balanced Counselling Strategy with method cards<br>vs. no training	% receiving LARC at enrolment consultation	**Uptake**<br>Increased from 15% pre-intervention to 40% post-intervention (no p-value reported)
49	Wu 2003 (China)	Married women attending FP clinic (n=1,367)	Non-randomised controlled pre-post study	Training of providers in contraceptive counselling based on GATHER approach and principles/topics to cover<br>vs. no training	% "satisfied" with counselling service	**Satisfaction**<br>Increased in intervention group from 49.1% at baseline to 58.9% post-intervention (p=0.0219 across three satisfaction categories), but no increase in control group (57.9% vs. 55.1%, p=0.2032).<br>No difference between intervention and control groups post-intervention (p=0.5396).
	Patient coaching intervention					
73	Kim 2003 (Indonesia)	Women attending clinics for FP (n=768)	Randomised controlled trial	Patient coaching in assertiveness and question preparation + Smart Patient pamphlet + routine consultation<br>vs. HIV/AIDS leaflet + routine consultation	• % discontinuation at 8 month follow-up<br>• % life table continuation	**Continuation**<br>Among new users, no difference in discontinuation between intervention and control group (3.9% vs. 7.8%, p>0.05).<br>Among new users, weak evidence of higher life table continuation in intervention than control group (89% vs. 85%, p=0.08).

Cavallaro FL, *et al. BMJ Sex Reprod Health* 2020; 46:254–269. doi: 10.1136/bmjsrh-2019-200377

Ref. no.	Study (country)	Population (sample size)	Study design	Intervention vs. comparison	Outcome definition	Results
	**INTERVENTIONS TARGETING WOMEN UNDERGOING ABORTION (N=11)**					
	Additional counselling					
57	Bender 2004 (Iceland)	Women requesting first-trimester abortion (n=420)	Randomised controlled trial	Interview with FP nurse on reasons for non-use and plans for future use + routine counselling<br>vs. routine counselling only	% modern contraceptive use 4-6 months post-abortion	**Use**<br>No difference between intervention and control groups (86.5% vs 85.2%, p=0.752)
60	Davidson 2015 (USA)	Young women (18-29yo) undergoing surgical abortion (n=191)	Randomised controlled trial	Video on LARC information and client narratives + routine counselling<br>vs. video on stress management + routine counselling (free contraceptive provision for all participants)	• % initiating LARC at abortion consultation<br>• % receiving no modern method at abortion consultation<br>• Mean satisfaction score with birth control decision (max: 5)<br>• Mean satisfaction score with counselling (max: 5)	**Uptake**<br>No difference in LARC initiation between intervention and control groups (59.4% vs. 51.6%, p=0.278).<br>**Use**<br>No difference in no modern method received between intervention and control groups (4.2% vs. 4.2%, p=0.988).<br>**Satisfaction**<br>No difference in satisfaction with birth control decision between intervention and control group (4.71 vs. 4.72, p=0.94).<br>No difference in satisfaction with counselling between intervention and control group (4.77 vs. 4.74, p=0.82)
58	Schunmann 2006 (UK)	Women requesting abortion (n=613)	Randomised controlled trial	Counselling by specialist physician + expanded contraceptive provision + routine counselling<br>vs. routine counselling + reduced contraceptive provision	• % women receiving no method or LARC during abortion consultation<br>• % LARC method use at 16 week follow-up<br>• % continuing same method at 16 weeks	**Uptake**<br>Higher implant receipt in intervention than control group (surgical abortion [SA]: 16.0% vs 2.0%, p=0.004; medical abortion [MA]: 22.1% vs. 5.5%, p<0.001).<br>No difference in IUD/IUS or sterilisation/vasectomy (p non-significant for all, no values reported).<br>**Use**<br>Lower receipt of no method in intervention than control group (SA: 4.3% vs. 15.3%, p<0.001; MA: 3.2% vs. 20.5%, p=0.031).<br>Higher use of implants at 16 weeks in intervention than control group (19% vs. 5%, p<0.001).<br>No difference in use of any method, IUD/IUS or sterilisation/vasectomy at 16 weeks (p non-significant for all, no values reported).<br>**Continuation**<br>No difference in continuation of method received (p non-significant for all methods, no values reported)
62	Smith 2015 (Cambodia)	Women seeking abortion (n=500)	Randomised controlled trial	Automated voice messages and phone counsellor support + routine counselling<br>vs. routine counselling	• % effective contraceptive use (modern methods <10% pregnancy rate) at 4 and 12 months<br>• % discontinuation among women who initiated	**Use**<br>Higher at 4 months in intervention than control group (64% vs. 46%, unadjusted RR=1.39; 95% CI: 1.17-1.66).<br>No difference at 12 months between intervention and control group (50% vs 43%; unadjusted RR=1.16; CI: 0.92-1.47).<br>**Continuation**

INTERVENTIONS TARGETING WOMEN UNDERGOING ABORTION (N=11)

Ref. no.	Study (country)	Population (sample size)	Study design	Intervention vs. comparison	Outcome definition	Results
					effective contraceptive use within 4 weeks of abortion	Weak evidence of lower discontinuation at 4 months in the intervention than control group (7% vs 16%; unadjusted HR=0.45; CI: 0.20-1.01), but no difference at 12 months (26% vs 30%; unadjusted HR=0.82; CI: 0.48-1.40).
59	Whitaker 2016 (USA)	Young women (15-29yo) requesting abortion (n=60)	Randomised controlled trial (pilot)	Motivational interviewing-based counselling + routine counselling vs. routine counselling only (non-standardised)	• % effective contraceptive and LARC initiation within 4 weeks post-abortion<br>• % effective contraceptive and LARC use at 3 months post-abortion<br>• % high satisfaction with method<br>• % satisfaction with counselling	**Uptake**<br>No difference in effective contraception initiation within 4 weeks between intervention and control groups (86.2% vs. 74.2%, p=0.34).<br>Higher LARC initiation in intervention than control group (65.5% vs. 32.3%, p=0.01).<br>**Use**<br>No difference in contraceptive use at 3 months (84.0% vs. 61.5%, p=0.12).<br>Weak evidence of higher LARC use at 3 months (60.0% vs. 30.8%, p=0.05).<br>**Satisfaction**<br>Among women using effective contraception at 3 months, no difference in high satisfaction with method between intervention and control group (90.5% vs. 68.8%, p=0.20).<br>Higher satisfaction with counselling in intervention than control group (92.0% vs. 65.4%, p=0.04).
66	Zhu 2009 (China)	Young women (<25yo) seeking abortion (n=2,336)	Cluster randomised controlled trial	Comprehensive package: individual counselling + free contraceptive provision + male partner involvement + essential package vs. essential package only (training of abortion providers + group education + referral to existing FP services)	% effective method use (condoms, pills, IUD, implants) at 6 months post-abortion	**Use**<br>Higher among women in hospitals with comprehensive than essential package (96.2% vs. 89.3%, no p-value given, OR=2.03, 95% CI: 1.04-3.98 adjusted for socio-economic and reproductive characteristics)
	Mode of counselling delivery					
20 & 22	Ferreira 2011 & 2015 (Brazil)	Women undergoing abortion (n=246)	Randomised controlled trial	Personalised and comprehensive individual counselling vs. group counselling (free contraceptive provision for all participants)	• % women receiving modern method<br>• % contraceptive use at 6 months and 2 years post-abortion<br>• % women "very satisfied" with method	**Use**<br>No difference in modern method receipt between intervention and control group (100% vs. 96.7%, p=0.122).<br>Higher contraceptive use at 6 months in intervention than control group (98.4% vs. 70.6%, p<0.001).<br>No difference at 2 years between intervention and control groups (75.4% vs. 81.5%, p=0.270).<br>**Satisfaction**<br>Higher in intervention than control group (80.5% vs. 33.3%, p<0.001)

Ref. no.	Study (country)	Population (sample size)	Study design	Intervention vs. comparison	Outcome definition	Results
63	Lohr 2018 (UK)	Women undergoing abortion and receiving contraception from British Pregnancy Advisory Service (n=18,573)	Non-randomised controlled study	Telephone counselling with trained nurse prior to abortion<br>vs. face-to-face counselling by trained nurse during abortion consultation	% women receiving tier 1 method	**Uptake**<br>Higher among women in intervention than control group 57.7% vs. 48.2%, p<0.001; OR=1.60, 95% CI: 1.42-1.71 adjusted for abortion type, weeks' gestation, age, relationship abortion, reproductive and socio-economic characteristics).
	Content					
37	Langston 2010 (USA)	Women seeking procedure for first trimester spontaneous or induced abortion (n=222)	Randomised controlled trial	Structured counselling using WHO decision-making tool flipchart<br>vs. routine non-standardised counselling	% continuing to use very effective (IUD, IUS, implant, sterilisation) and effective (injectable, ring, patch, pill) method at 3 months post-abortion	**Continuation**<br>Among women choosing very effective method, no difference in continuation between intervention and control group (85% vs. 77%, p=0.28; adjusted OR=1.24, 95% CI: 0.62-2.50, covariates unspecified).<br>Among women choosing effective method, no difference in continuation between intervention and control group (68% vs. 68%, p=0.96; adjusted OR=1.43, 95% C: 0.58-3.52, covariates unspecified).
50	Savelieva 2003 (Russia)	Women undergoing abortion (n=516)	Uncontrolled pre-post study	Pre-training of providers<br>vs. post-training model 1. training in interpersonal communication and counselling + counselling aids + client materials<br>vs. post-training model 2. training + contraceptive commodities	• % discharged with contraceptive method<br>• % modern method use at 12 months<br>• % satisfied with overall services<br>• % "would recommend facility to a friend"	**Use**<br>Lower receipt of method at discharge pre-intervention and model 1 groups than model 2 group (pre-intervention: 1.0% vs. model 1: 1.2%, vs. model 2: 61.1%, p<0.05 both comparing model 2 to model 1 and to control group).<br>Higher use at 12 months post-intervention than pre-intervention (pre-intervention: 53.3% vs. model 1: 62.0% vs. model 2: 66.7%, p<0.05 comparing model 1 and model 2 to pre-intervention).<br>**Satisfaction**<br>No difference in satisfaction with services (control: 91.3% vs. model 1: 92.9% vs. model 2: 94.0%, p>0.05).<br>Higher recommendations to friend pre-intervention and in model 2 group than model 1 group (pre-intervention: 86.8%, model 1: 75.8%, model 2: 83.0%, p<0.05 comparing model 1 to control group, and model 1 to model 2 group).
	Husband counselling					
80	Abdel-Tawab 1997 (Egypt)	Women seeking abortion (n=366)	Randomised controlled trial	Husband counselling + individual woman counselling<br>vs. individual woman counselling	% contraceptive use at 1 month post-abortion	**Use**<br>No difference between intervention and control group (14.0% vs. 15.3%, p non-significant, no values reported).
	**INTERVENTIONS FOR POSTPARTUM CONTRACEPTIVE INITIATION (N=13)**					
	Number/timing of sessions					

INTERVENTIONS FOR POSTPARTUM CONTRACEPTIVE INITIATION (N=13)

Ref. no.	Study (country)	Population (sample size)	Study design	Intervention vs. comparison	Outcome definition	Results
68	Adanikin 2013 (Nigeria)	Women receiving antenatal care from study hospital (n=216)	Randomised controlled trial	3 antenatal counselling sessions vs. routine 6 week postpartum counselling	% modern contraceptive use at 6 months postpartum	**Use**<br>Higher in intervention than control group (57.4% vs. 35.4%, p=0.002)
69	Bolam 1998 (Nepal)	Women delivering in study hospital (n=540)	Randomised controlled trial (four arms)	A: Immediate postpartum + 3 month health education vs. B: immediate postpartum health education vs. C: 3 month health education	% contraceptive use at 6 months postpartum	**Use**<br>Higher in intervention groups with immediate postpartum health education than without (A: 35%, B: 38%, C: 26%, no p-value for this comparison reported)
71	Gilliam 2004 (USA)	Young African-American women (≤25yo) receiving prenatal care at study facility, initiating the pill (n=33)	Randomised controlled trial	Pre-discharge nurse counselling, video and education materials + routine physician counselling vs. routine physician counselling	% pill and all-method continuation at 1 year postpartum	**Continuation**<br>Pill continuation was lower in intervention than control group (4/16 (25%) vs. 3/9 (33%), no p-value reported)<br>All method continuation was higher in the intervention than control group (12/16 (75%) vs. 6/9 (67%), no p-value reported).
72	Kaewkiattikun 2017 (Thailand)	Adolescent women (10-19yo) giving birth at study hospital (n=240)	Randomised controlled trial	Pre-discharge counselling + routine 4-6 week postpartum counselling vs. routine 4-6 week counselling	% modern contraceptive and LARC use at 4-6 weeks postpartum	**Use**<br>Higher modern contraceptive use in intervention than control group (100% vs. 92.2%, p<0.001 for all method distribution)<br>Higher LARC use in intervention than control group (73.7% vs. 42.6%, p<0.001; OR=3.67, 95% CI: 2.10-6.41 adjusted for education, pregnancy intention and parity)
70	Vural 2016 (Turkey)	Women receiving antenatal care and delivering at study hospital (n=200)	Non-randomised controlled study	6 week postpartum counselling + routine antenatal counselling vs. routine antenatal counselling	% contraceptive use at 6 month follow-up	**Use**<br>Weak evidence higher in intervention than control group (60.8% vs. 49%, p=0.093)
	Timing/mode of counselling delivery					
67	Smith 2002 (UK, China, South Africa)	Women receiving antenatal care in study facilities (n=604 in Edinburgh, n=527 in Shanghai, n=506 in Cape Town)	Randomised controlled trial	Tailored antenatal FP nurse counselling vs. routine pre-discharge and 6 week postpartum counselling (Edinburgh), group antenatal sessions (Shanghai), group antenatal sessions and pre-discharge counselling (Cape Town)	% modern contraceptive use at 16 weeks postpartum, contraceptive use at 1 year postpartum	**Use**<br>No difference in modern contraceptive use at 16 weeks between intervention and control group (Edinburgh: 88% vs. 86%, Shanghai: 48% vs. 46.4%, Cape Town: 96% vs. 94%, no p-values reported)<br>No difference in contraceptive use at 1 year between intervention and control group (Edinburgh: 83% vs. 79%, Shanghai: 90% vs. 87%, Cape Town: 87% vs. 84%, no p-values reported)

INTERVENTIONS FOR POSTPARTUM CONTRACEPTIVE INITIATION (N=13)

Ref. no.	Study (country)	Population (sample size)	Study design	Intervention vs. comparison	Outcome definition	Results
	Mode of counselling delivery					
64	Akman 2010 (Turkey)	Women attending study facilities antenatally (n=180)	Randomised controlled trial	Antenatal contraceptive counselling<br>vs. antenatal leaflet + questions answered	% modern contraceptive use 6-9 months postpartum	**Use**<br>No difference between intervention and control groups (74.0% vs. 66.0%, p>0.05)
65	Proctor 2006 (USA)	Women receiving antenatal care in study facilities (n=319)	Randomised controlled trial (three arms)	First day postpartum physician counselling<br>vs. video<br>vs. leaflet<br>(all groups receive antenatal physician counselling)	% satisfied with counselling	**Satisfaction**<br>Higher in physician counselling group (99.0%) than video and leaflet counselling groups (91.3% and 93.0%, respectively; p=0.0439)
	Content					
51	Fatima 2018 (Bangladesh)	Women giving birth in study hospitals (n=27,622)	Uncontrolled pre-post study	Pre-<br>vs. post-refresher training on postpartum contraception (including IUD)	• % postpartum IUD insertions<br>• % IUD removals at 4-6 week follow-up<br>• % "satisfied" or "very satisfied" with counselling	**Uptake**<br>No difference in IUD insertions pre- vs. post-intervention (9.5% vs. 9.8%, p=0.42)<br>**Continuation**<br>Among women attending 4-6 week follow-up, no difference in IUD removals pre- vs. post-intervention (2.8% vs. 1.8%, p=0.41)<br>**Satisfaction**<br>No difference pre- vs. post-intervention (9.9+69.7 = 79.6% vs. 13.6+60.3 = 73.9%, no p-values reported)
53	Hardy 1998 (Brazil)	Women giving birth in study hospital (n=1,998)	Uncontrolled pre-post study	Intensive counselling on lactational amenorrhea as contraception<br>vs. routine counselling on breastfeeding and contraception	% modern method use at 12 months postpartum	**Use**<br>Higher in the intervention than control group (77.7% vs. 58.5%, p<0.0001 across all different methods; coefficient estimated=1.11 adjusting for socio-economic and reproductive characteristics, p=0.0002)
54	Ndegwa 2014 (Kenya)	Women receiving antenatal care in study hospital (n=127)	Randomised controlled trial	Intensive<br>vs. routine antenatal counselling	• % postpartum IUD insertion<br>• % IUD removals at 6 week follow-up<br>• % "client satisfaction"	**Uptake**<br>No difference in IUD insertion between intervention and control groups (78% * 63.3% = 49% vs. 66% * 64.3% = 42%, p=0.129 for acceptance and p=0.232 for uptake among acceptors)<br>**Continuation**<br>No difference in IUD removals between intervention and control groups (92% vs. 89%, p=0.235)<br>**Satisfaction**<br>No difference between intervention and control group (92% vs. 93%, p=1.00)

Cavallaro FL, *et al. BMJ Sex Reprod Health* 2020; 46:254–269. doi: 10.1136/bmjsrh-2019-200377

Ref. no.	Study (country)	Population (sample size)	Study design	Intervention vs. comparison	Outcome definition	Results
52	Tomlin 2017 (USA)	Adolescent women (13-17yo) receiving antenatal care in study facilities (n=309)	Non-randomised controlled study	Motivational interviewing adolescent-focused antenatal counselling every 1-2 weeks vs. routine antenatal counselling	% LARC use and no method use at 13 weeks postpartum	**Use**<br>Higher LARC use in intervention than control group (40.9% vs. 15.2%, p<0.01; OR=2.8, 95% CI: 1.5-5.2, adjusted for socio-economic characteristics)<br>No difference in no method use between intervention and control group (8.2% vs. 8.7%, p=0.88)
55	Torres 2018 (USA)	Women giving birth to preterm baby in study hospital (n=134)	Randomised controlled trial	Structured postpartum counselling on all methods by effectiveness tier vs. ad hoc, unstructured counselling	• % LARC (tier 1) use at 3 months postpartum<br>• Mean visual analog score of contraceptive method satisfaction (max: 100)	**Uptake**<br>Higher in intervention than control group (51% vs. 31%, p<0.05)<br>**Satisfaction**<br>No difference between intervention and control group (67 vs. 75, p=0.18)
	**INTERVENTIONS TO SYSTEMATISE PROVISION OF CONTRACEPTIVE COUNSELLING (N=4)**					
75	Gillespie 2009 (Ethiopia)	Women attending HIV voluntary counselling and testing (VCT) in semi-urban facilities (n=8,046)	Uncontrolled pre-post study	Training of VCT providers in contraceptive counselling + systematic counselling based on registers vs. ad hoc counselling	% receiving contraception during consultation	**Use**<br>Increased from 0% pre-intervention to 6% post-intervention (no p-value reported)
78	Grubb 2018 (USA)	Adolescents (11-17yo) admitted to juvenile detention centre (n=306)	Uncontrolled pre-post study	Systematic, standardised counselling based on motivational interviewing principles + leaflet on contraceptive methods vs. ad hoc, non-standardised counselling at intake physical examination	% receiving contraception at intake physical examination	**Use**<br>Increased from 7% pre-intervention to 44-54% post-intervention (after/before requiring parental consent, p-value significant, no value reported)

Ref. no.	Study (country)	Population (sample size)	Study design	Intervention vs. comparison	Outcome definition	Results
76	Lee 2015 (USA)	Women seeking pregnancy test at walk-in clinic wishing to avoid pregnancy for 6 months (n=323)	Uncontrolled pre-post study	Systematic counselling based on checklist + offer of same-day emergency contraception (EC), IUD or implant placement<br>vs. ad hoc, non-standardised counselling + offer of emergency contraception only	• % receiving same-day EC, IUD and implant<br>• % initiating any method and highly effective reversible contraception (intrauterine, subdermal or injectable) within 3 months post-visit<br>• % switching to more effective method between pregnancy testing and 3 months post-visit	**Uptake**<br>EC receipt increased from 5.3% pre-intervention to 21.9% post-intervention p<0.001, OR=4.66, 95% CI: 1.76-12.35, adjusted for age, ethnicity, insurance, method use prior to visit).<br>Same-day IUD placement increased from 0% to 4.4% (p=0.04).<br>No difference in same-day implant placement (0% vs. 0.9%, p=0.99).<br>Among women not using contraception at pregnancy test, increase in contraceptive initiation by 3 months from 16.9% pre-intervention to 39.5% post-intervention (p<0.001).<br>**Use**<br>Increase in highly effective reversible contraception at 3 months from 18.3% pre-intervention to 32.4% post-intervention (p=0.03, OR=1.91, 95% CI: 0.94-3.90 adjusted for age, ethnicity, insurance, and method use prior to visit).<br>**Switching**<br>Increased from 26.8% to 49.7% (p=0.001, OR=2.02, 95% CI: 1.03-3.96 adjusted for age, ethnicity, insurance, and method use prior to visit).
77	Yassin 2005 (UK)	Women attending abortion clinic (n=522)	Uncontrolled pre-post study	Systematic counselling by FP nurses trained in counselling<br>vs. ad hoc counselling by abortion counsellor	• % women receiving contraception<br>• % women receiving less user-dependent method (IUD, IUS, implant, injection)	**Use**<br>Method receipt increased from 40% pre-intervention to 96% post-intervention (no p-value reported).<br>Less user-dependent method receipt increased from 12% pre-intervention to 73% post-intervention (no p-value reported)
	**COMMUNITY-BASED INTERVENTIONS (N=4)**					
	Peer counselling					
74	Ferguson 1998 (USA)	African-American adolescents (12-16yo) in low-income neighbourhood (n=63)	Randomised controlled trial	Peer counsellor-led group discussions on sexual health and contraception<br>vs. adult counsellor-led group discussions	% effective contraceptive use at last intercourse at 8 weeks and 3 months after enrolment	**Use**<br>Among participants reporting information on sexual activity, lower at 8 weeks in intervention than control group (38% vs. 58%, no p-value reported); lower at 3 months in intervention than control group (25% vs. 33%, no p-value reported)
	Couples counselling					
81	El-Khoury 2016 (Jordan)	Married women not using modern contraception living in catchment area (n=1,247)	Randomised controlled trial	Home-based couples counselling by trained female counsellor<br>vs. individual counselling	% modern contraceptive use at 6 months after intervention	**Use**<br>No difference between intervention and control group (absolute difference: 1.5 percentage points, unadjusted p=0.682, p=0.660 adjusted for socio-demographic and reproductive characteristics).

COMMUNITY-BASED INTERVENTIONS (N=4)

Ref. no.	Study (country)	Population (sample size)	Study design	Intervention vs. comparison	Outcome definition	Results
83	Lemani 2017 (Malawi)	Young (<30yo) never-users with male partner living in catchment area (n=808)	Cluster randomised controlled trial	Health Surveillance Assistants training in couples counselling vs. no training	• % initiating pill or injectable at initial home visit<br>• % receiving a method at 6 month follow-up home visit	**Uptake**<br>No difference between intervention and control groups (98% vs. 99%, p=0.22). **Use**<br>Weak evidence of higher method receipt at 6 months in intervention than control group (97.4% vs. 94.2%, p=0.064).
82	Terefe 1993 (Ethiopia)	Married women living in catchment area (n=527)	Randomised controlled trial	Home-based couples counselling by female health assistants vs. individual counselling	% modern contraceptive use at 2 months and 12 months after intervention	**Uptake**<br>Higher at 2 months in intervention than control group (24.7% vs. 15.3%, no p-value reported; unadjusted RR=1.61, 95% CI: 1.13-2.30).<br>**Use**<br>Higher at 12 months in intervention than control group (32.8% vs. 17.2%, no p-value reported; unadjusted RR=1.90, 95% CI: 1.36-2.66).

Supplementary table 3. Advantages and disadvantages of counselling interventions in included studies, and implications for implementation

Advantages	Disadvantages	Implications for implementation
All counselling interventions		
• May lengthen or shorten contraceptive consultation	• May lengthen or shorten contraceptive consultation<br>• Limitations of available staff and resources at the time and place of counselling affects uptake (e.g. postpartum visit, abortion consultation, or facilities where providers for LARC insertion are not always present)<br>• Method cost (especially for LARC) is an issue for women in many settings<br>• Counselling intervention alone may not have effect if lack of providers and key resources, or if quality of care is already high<br>• In some studies, intervention and control care not described in sufficient detail	• Importance of tailoring counselling to patient flow and services available within each facility<br>• Importance of embedding counselling improvements within broader quality of care improvements<br>• Importance of monitoring impact of counselling interventions on consultation length and patient volume
Structured counselling for specific method requesters/adopters		
• May avert costs related to discontinuation (LARC removal)	• Counselling on menstrual side-effects may not affect continuation in settings where main reasons for discontinuation are unrelated to side-effects (e.g. weight gain or IUD expulsion)	• Women should receive in-depth counselling (including on side-effects) prior to LARC insertion where access to removal services is poor<br>• In the majority of settings without access to reversal of tubal ligation, women should be assessed for likelihood of regret prior to sterilisation and counselled on other methods if ambivalent<br>• Women receiving tubal ligation during caesarean section should be counselled antenatally
Structured/standardised counselling (for new method users)		
• Ensures women receive information on all methods<br>• May use less provider time, if performed by a non-clinician counsellor	• Structured script may not allow tailoring to individual women and waste time describing methods that are not desired by women<br>• May lengthen consultations and use more provider time (if performed by provider)<br>• May lead to information overload<br>• Low provider adherence in some settings	• Importance of monitoring provider counselling performance to assess adherence over time, and of refresher trainings<br>• Need to consider implications for staffing and clinic flow of potential increased consultation duration<br>• Importance of providing client education materials (adapted to literacy level) to take home for reference
Provider training in counselling skills (including paper decision-making tools and client-provider interaction)		
• Training on decision-making tool use may improve client-provider interaction, verbal and non-verbal communication (eye contact)<br>• Positive reaction from clients and providers to shared decision-making brochures in some settings<br>• Training and continuation (e.g. printing) costs relatively low<br>• May or may not increase length of consultation (depending on prior practice)	• May not have an effect on contraceptive practice if other clinical skills are poor (e.g. LARC insertion)<br>• Training on decision-making tools may not have effect on other aspects of provider performance<br>• Motivational interviewing training very demanding, leading to shortfall in trained providers<br>• Training may be time-intensive for providers (e.g. Balanced Counselling training in some studies of 2 full days followed by 1 day retraining)<br>• May increase costs (e.g. if more LARC insertions occur as a result of training)<br>• Cost of paper tool development and supervision may also be high	• Importance of monitoring provider counselling performance to assess compliance over time, and assess performance under real world circumstances<br>• Provider training needs to include plans for refresher trainings and new staff training from staff turnover<br>• Importance of monitoring which clients consistently receive lower quality care and which providers consistently provide low quality care to target remedial trainings<br>• Importance of assessing provider clinical skill level for all methods offered as well as counselling skills<br>• Plan for additional resource needs related to potential changes in method mix

Cavallaro FL, *et al. BMJ Sex Reprod Health* 2020; 46:254–269. doi: 10.1136/bmjsrh-2019-200377

Advantages	Disadvantages	Implications for implementation
Digital decision-making tools		
• Interactive (easy to tailor to individual clients)<br>• Easy to update<br>• Tablet and phone-based apps more portable than computer-based tools<br>• Positive reaction from digitally literate populations (e.g. adolescents)<br>• May save provider time and make use of waiting time<br>• Printout of client characteristics, preferences and questions can be used as bridge between tool and consultation<br>• Consultation with client group can help design a client-centred tool incorporating desired information and format<br>• Can be placed in non-clinical/community settings for information and link to services	• May require additional support staff time (explanations and set up)<br>• Intervention at initiation only may not have effect on continuation<br>• May not be effective in populations with low digital literacy<br>• May be expensive to develop a tailored tool<br>• Printout used inconsistently in some settings because additional form not integrated into routine forms<br>• Satisfaction is lower when comparing digital tools only to face-to-face counselling	• Digital tools should be used in conjunction with face-to-face counselling, not as a replacement<br>• Interaction with tools should be integrated into clinic patient flow, and any printouts into the record flow
Video counselling		
• May save provider time and make use of waiting time<br>• Easily reproducible/standardised intervention<br>• Consultation with client group can help design a video tailored to client population concerns	• May require additional support staff time (set up)<br>• May be expensive to develop a tailored video for specific setting<br>• Difficult to tailor video to individual client	• Importance of integrating video counselling into clinic patient flow<br>• Assess suitability with client population, if using existing video
Telephone-based counselling (including messaging)		
• Automated text and voice messaging can reach large populations at low cost<br>• Adolescents and young women are active mobile phone users in some settings, making them easy to reach<br>• May improve access to services when no cost to end user<br>• Messages can be tailored to user (language, duration, voice/message mode, health literacy level, method used, concerns)<br>• Telephone call counselling can be scheduled at convenient time for client (both ongoing and for e.g. peri-abortion counselling)	• Mobile and young populations may change phone numbers frequently<br>• Cannot reach women without mobile phone (potentially those most in need)<br>• May represent access barrier if cost of messages/calls born by participants<br>• Effect tailed off rapidly after text messaging intervention, suggesting effects do not last<br>• Less interactive than other digital platforms<br>• Telephone counselling requires availability of phone counsellors<br>• Many attempts per successful phone call in some populations, making it time-consuming and expensive	• Suitability of phone-based counselling for target population should be assessed<br>• Effective strategies to minimise loss-to-follow up are needed<br>• Automated text and voice interventions should include contact information for providers or counsellors if want further discussion on contraception
Group counselling		
• More time efficient	• Does not allow much tailoring of counselling	
Patient coaching interventions		
• Takes advantage of client waiting time while saving clinician time (did not lengthen consultation in one setting)<br>• Intervention with women immediately prior to provider consultation to maximise impact	• Single coaching session may not have lasting effect on client-provider interactions	• May require repeated coaching sessions
Counselling for women undergoing an abortion		
• Counselling prior to abortion (e.g. telephone) may provide opportunity for fuller discussion on contraceptive methods, give women more time to think about method choice and more confidence to initiate chosen method at abortion (especially if surgical abortion under general anaesthesia)<br>• Pre-abortion counselling may also save time during abortion consultation	• Counselling at abortion consultation may be limited by time availability and information overload<br>• Some women may be reluctant to have immediate initiation<br>• All contraceptive methods not always available at abortion consultation<br>• Counselling before or after abortion relies on effective follow-up mechanism	• All abortion clients should receive counselling on contraception, including raising availability of emergency contraceptive methods and in-depth discussion of challenges with effective contraceptive use<br>• Importance of tailoring counselling to patient flow and contraceptive services available (including counselling before/after abortion)

Advantages	Disadvantages	Implications for implementation
• Women may also benefit from consultation shortly after abortion to facilitate informed choice and allow for tailored counselling		• Consider integration of contraception services at abortion consultation to ensure women facilitate immediate initiation<br>• Effective follow-up mechanisms are needed among women who do not want to initiate at the time of abortion
Counselling strategies for pregnant or postpartum women		
• Antenatal or immediate postpartum counselling can help capture women who do not return for routine postpartum care, as well as women who have resumed sexual activity before postpartum visit (often 4-6 weeks), and encourage women to attend routine postpartum visit<br>• Antenatal counselling may provide time to reflect on appropriate postpartum method (shown to have an effect on LARC uptake among women who did not plan to use LARC)<br>• Immediate postpartum counselling can make the most of hospitalisation if long enough (potentially by non-clinicians to save provider time)<br>• 6 week postpartum counselling women can be immediately referred for contraception<br>• Counselling on LAM does not push contraception on women not ready for it (may improve attendance at postpartum visit)	• Multiple sessions uses more provider time and more of a burden for women than single session<br>• Hospital stay may be too short to provide contraceptive counselling<br>• Immediate postpartum contraception is not always accepted by women<br>• Women with adverse birth outcomes may not prioritise contraceptive counselling (women with preterm birth have high refusal to participate)<br>• Follow-up of young mothers is a challenge in some settings<br>• Many factors affect postpartum contraceptive use, antenatal counselling may not effectively target them<br>• Provider resistance to counselling on LAM in one setting	• Women should be offered multiple sessions in antenatal and postpartum period, with possibility of initiation immediately after delivery or at routine postpartum visits<br>• Routine postpartum visit around 3-4 weeks (rather than 6) enables reaching women before resuming sexual activity<br>• Adequate follow-up mechanisms are required to maximise attendance at routine postpartum visit<br>• Antenatal and immediate postpartum counselling should include conditions under which LAM is effective
Systematic counselling in non-contraception outpatient services		
• Relatively low cost intervention<br>• Easy (usually modification of client record sheets as prompt with provider training)	• May not have effect on contraceptive practice if women require referral elsewhere to receive methods<br>• All women may not be sexually active or in need of contraception, may increase cost of intervention per woman receiving contraception<br>• May only concern few women (e.g. most women do not go to clinic for pregnancy testing)<br>• Needs careful consideration of process/flow of care to be successfully integrated	• Reproductive health needs of target population should be assessed prior to determining whether contraceptive counselling should be offered systematically<br>• Importance of voluntary consent in vulnerable populations (e.g. VCT or detained women)
Partner counselling		
• May target main contraceptive decision-maker in some settings	• Women may not want husbands to be counselled, with implications for women's safety<br>• Husband availability may limit feasibility (logistical challenges)<br>• In some settings, male partners not allowed in abortion consultation or lack of male waiting area or staff discouragement, limits male involvement	• Importance of strict consent procedures to ensure husband counselling only among women who provide informed consent (especially if some women using covertly)<br>• Flexibility in scheduling is required to find time suited to partner availability
Community-based interventions		
• May be cost-effective if piggy-backing off of existing programmes	• Repeated programmes demand substantial resources if not piggybacked onto existing community-based interventions<br>• In interventions targeting non-users, some women initiated >2 months after intervention	• Multiple contacts may be needed if women were not intending to use contraception

Cavallaro FL, *et al. BMJ Sex Reprod Health* 2020; 46:254–269. doi: 10.1136/bmjsrh-2019-200377
